# Early Last Interglacial ocean warming drove substantial ice mass loss from Antarctica

**DOI:** 10.1073/pnas.1902469117

**Published:** 2020-02-11

**Authors:** Chris S. M. Turney, Christopher J. Fogwill, Nicholas R. Golledge, Nicholas P. McKay, Erik van Sebille, Richard T. Jones, David Etheridge, Mauro Rubino, David P. Thornton, Siwan M. Davies, Christopher Bronk Ramsey, Zoë A. Thomas, Michael I. Bird, Niels C. Munksgaard, Mika Kohno, John Woodward, Kate Winter, Laura S. Weyrich, Camilla M. Rootes, Helen Millman, Paul G. Albert, Andres Rivera, Tas van Ommen, Mark Curran, Andrew Moy, Stefan Rahmstorf, Kenji Kawamura, Claus-Dieter Hillenbrand, Michael E. Weber, Christina J. Manning, Jennifer Young, Alan Cooper

**Affiliations:** ^a^Palaeontology, Geobiology and Earth Archives Research Centre, School of Biological, Earth and Environmental Sciences, University of New South Wales, Kensington NSW 2033, Australia;; ^b^Australian Research Council Centre of Excellence in Australian Biodiversity and Heritage, School of Biological, Earth and Environmental Sciences, University of New South Wales, Kensington NSW 2033, Australia;; ^c^Chronos ^14^Carbon-Cycle Facility, University of New South Wales, Sydney NSW 2052, Australia;; ^d^School of Geography, Geology and the Environment, Keele University, Staffordshire ST5 5BG, United Kingdom;; ^e^Antarctic Research Centre, Victoria University of Wellington, Wellington 6140, New Zealand;; ^f^Environment and Climate, GNS Science, Avalon, Lower Hutt 5011, New Zealand;; ^g^School of Earth and Sustainability, Northern Arizona University, Flagstaff, AZ 86011;; ^h^Grantham Institute, Imperial College London, London SW7 2AZ, United Kingdom;; ^i^Department of Physics, Imperial College London, London SW7 2AZ, United Kingdom;; ^j^Institute for Marine and Atmospheric Research Utrecht, Utrecht University, 3584 CS Utrecht, The Netherlands;; ^k^Department of Geography, Exeter University, Devon EX4 4RJ, United Kingdom;; ^l^Climate Science Centre, Commonwealth Scientific and Industrial Research Organisation Ocean and Atmosphere, Aspendale, VIC 3195 Australia;; ^m^Dipartimento di Matematica e Fisica, Università della Campania “Luigi Vanvitelli,” 81100 Caserta, Italy;; ^n^Department of Geography, Swansea University, Swansea SA2 8PP, United Kingdom;; ^o^Research Laboratory for Archaeology and the History of Art, University of Oxford, Oxford OX1 3TG, United Kingdom;; ^p^Centre for Tropical Environmental and Sustainability Science, College of Science and Engineering, James Cook University, Cairns, QLD 4870, Australia;; ^q^Australian Research Council Centre of Excellence in Australian Biodiversity and Heritage, James Cook University, Cairns, QLD 4870, Australia;; ^r^Research Institute for the Environment and Livelihoods, Charles Darwin University, Darwin NT 0909, Australia;; ^s^Department of Geochemistry, Geoscience Center, University of Göttingen, 37077 Göttingen, Germany;; ^t^Department of Geography and Environmental Sciences, Faculty of Engineering and Environment, Northumbria University, Newcastle upon Tyne NE1 8ST, United Kingdom;; ^u^Australian Centre for Ancient DNA, University of Adelaide, Adelaide SA 5005, Australia;; ^v^Australian Research Council Centre of Excellence in Australian Biodiversity and Heritage, University of Adelaide, Adelaide SA 5005, Australia;; ^w^Department of Geography, University of Sheffield, Sheffield S3 7ND, United Kingdom;; ^x^Departamento de Geografia, Universidad de Chile, 8331051 Santiago, Chile;; ^y^Department of the Environment and Energy, Australian Antarctic Division, Kingston, TAS 7050, Australia;; ^z^Antarctic Climate and Ecosystems Cooperative Research Centre, University of Tasmania, Hobart, TAS 7001, Australia;; ^aa^Earth System Analysis, Potsdam Institute for Climate Impact Research, D-14412 Potsdam, Germany;; ^bb^Institute of Physics and Astronomy, University of Potsdam, 14476 Potsdam, Germany;; ^cc^Research Organizations of Information and Systems, National Institute of Polar Research, Tachikawa, Tokyo 190-8518, Japan;; ^dd^Department of Polar Science, Graduate University for Advanced Studies, Tachikawa, Tokyo 190-8518, Japan;; ^ee^Institute of Biogeosciences, Japan Agency for Marine–Earth Science and Technology, Yokosuka 237-0061, Japan;; ^ff^Palaeo Environments, Ice Sheets and Climate Change, British Antarctic Survey, Cambridge CB3 0ET, United Kingdom;; ^gg^Steinmann Institute, University of Bonn, 53115 Bonn, Germany;; ^hh^Department of Earth Sciences, Royal Holloway University of London, Surrey TW20 OEX, United Kingdom;; ^ii^South Australian Museum, Adelaide, South Australia 5005, Australia

**Keywords:** Antarctic ice sheets, marine ice sheet instability (MISI), paleoclimatology, polar amplification, tipping element

## Abstract

The future response of the Antarctic ice sheet to rising temperatures remains highly uncertain. A useful period for assessing the sensitivity of Antarctica to warming is the Last Interglacial (LIG) (129 to 116 ky), which experienced warmer polar temperatures and higher global mean sea level (GMSL) (+6 to 9 m) relative to present day. LIG sea level cannot be fully explained by Greenland Ice Sheet melt (∼2 m), ocean thermal expansion, and melting mountain glaciers (∼1 m), suggesting substantial Antarctic mass loss was initiated by warming of Southern Ocean waters, resulting from a weakening Atlantic meridional overturning circulation in response to North Atlantic surface freshening. Here, we report a blue-ice record of ice sheet and environmental change from the Weddell Sea Embayment at the periphery of the marine-based West Antarctic Ice Sheet (WAIS), which is underlain by major methane hydrate reserves. Constrained by a widespread volcanic horizon and supported by ancient microbial DNA analyses, we provide evidence for substantial mass loss across the Weddell Sea Embayment during the LIG, most likely driven by ocean warming and associated with destabilization of subglacial hydrates. Ice sheet modeling supports this interpretation and suggests that millennial-scale warming of the Southern Ocean could have triggered a multimeter rise in global sea levels. Our data indicate that Antarctica is highly vulnerable to projected increases in ocean temperatures and may drive ice–climate feedbacks that further amplify warming.

## Significance

Fifty years ago, it was speculated that the marine-based West Antarctic Ice Sheet is vulnerable to warming and may have melted in the past. Testing this hypothesis has proved challenging due to the difficulty of developing in situ records of ice sheet and environmental change spanning warm periods. We present a multiproxy record that implies loss of the West Antarctic Ice Sheet during the Last Interglacial (129,000 to 116,000 y ago), associated with ocean warming and the release of greenhouse gas methane from marine sediments. Our ice sheet modeling predicts that Antarctica may have contributed several meters to global sea level at this time, suggesting that this ice sheet lies close to a “tipping point” under projected warming.

The projected contribution of the Antarctic ice sheet to 21st-century global mean sea level (GMSL) ranges from negligible (1) to several meters (2, 3). Valuable insights into the response of ice sheets to warming may be gained from the Last Interglacial (LIG) (or Marine Isotope Stage [MIS] 5e in marine sediment records; 129,000 to 116,000 y before present or 129 to 116 ky) (4–9). This period experienced warmer polar temperatures and higher GMSL (+6 to 9 m, possibly up to 11 m) (4, 10–13) relative to present day, and was the most geographically widespread expression of high sea level during a previous warm period (4, 10). LIG sea level cannot be fully explained by Greenland Ice Sheet melt (∼2 m) (8), ocean thermal expansion, and melting mountain glaciers (∼1 m) (4), implying substantial Antarctic mass loss (3, 4, 14, 15). Half a century ago, John Mercer was the first to propose that the marine-based West Antarctic Ice Sheet (WAIS) is vulnerable to a warming atmosphere through loss of buttressing ice shelves and may have made a significant contribution to global sea level during the LIG (5–7). Recent work has further demonstrated that extensive deep, marine-based sectors of the East Antarctic Ice Sheet (EAIS) may have accelerated melting, thus contributing to higher LIG sea levels (14). While an isotopic signature of a relatively cool LIG climate preserved in the Mount Moulton blue ice field (16) may be explained by substantial WAIS mass loss (17), no direct physical evidence has yet been identified (4, 18). Temperature estimates derived from climate model simulations provide an indirect measure of change but typically suggest ∼1 °C less warming than proxy-based reconstructions (4, 8, 19). When used to drive ice sheet models, these climate anomalies are not sufficient to remove the floating ice shelves that buttress ice flow from central Antarctica (20). In an attempt to bypass these problems, ice sheet models have been driven by a wide range of prescribed climate scenarios; however, these suggest widely different sensitivities dependent on model physics and parameterization (21, 22), with >2 °C (and in some instances >4 °C) ocean warming required for the loss of the WAIS, exceeding paleoclimate estimates (3, 9, 20, 23) and different sensitivities of Antarctic ice sheet sectors (18, 24, 25).

**Fig. 1.**
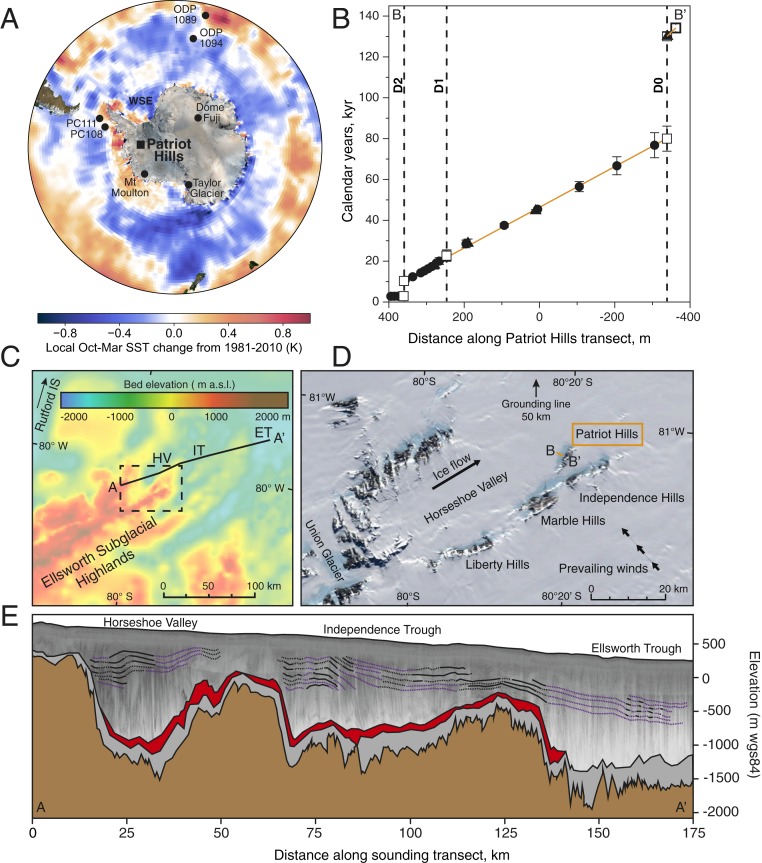
Location and age profile of the Patriot Hills BIA. (*A*) Location of Antarctic ice and marine records discussed in this study and austral spring–summer (October to March) SST trends (over the period 1981 to 2010; HadISST data). (*B*) Trace gas (circles), tephra (triangles), and boundary (square) age solutions for surface ice along transect B–B′ relative to an arbitrary datum along the transect (displayed in *D*). The dashed lines denote unconformities D0–D2 at their surface expression. (*C*) Basal topography of the Ellsworth Subglacial Highlands (West Antarctica) with the locations of airborne radio-echo sounding transect A–A′ (displayed in *E*) and Rutford Ice Stream (IS) (29). The Horseshoe Valley, Independence, and Ellsworth troughs are given by the initials HV, IT, and ET, respectively. (*D*) The location of Patriot Hills in Horseshoe Valley (LIMA background image) with the BIA climate line (marked by transect B–B′), dominant ice flow direction, and distance to grounding line. (*E*) Airborne radio-echo sounding cross-section of ice within Horseshoe Valley, Independence, and Ellsworth troughs (modified from ref. 29). Digitization highlights basal topography (brown), lower basal ice unit (gray), and upper basal ice unit (red) as well as internal stratigraphic features (black for observed, dashed for inferred, and purple for best estimate).

Here, we report a high-resolution record of environmental change and ice flow dynamics from the Patriot Hills Blue Ice Area (BIA), exposed in Horseshoe Valley (Ellsworth Mountains; *Methods*) (Fig. 1*A*). Horseshoe Valley is a locally sourced compound glacier system (i.e., with negligible inflow) that is buttressed by, but ultimately coalesces with, the Institute Ice Stream via the Horseshoe Valley Trough, making the area sensitive to dynamic ice sheet changes across the broader Weddell Sea Embayment (WSE) (26). Due to strong prevailing katabatic airflow, an extensive BIA (more than 1,150 m across) has formed to the leeward side of the Patriot Hills, where ancient ice is drawn up from depth within Horseshoe Valley (Fig. 1*E*). Regional airborne and detailed local ground-penetrating radar (GPR) surveys show a remarkably coherent series of dipping (24 to 45°) layers, broken by two discontinuities, which represent isochrons across the Patriot Hills BIA, extending thousands of meters into Horseshoe Valley. A “horizontal ice core” across the BIA spans the time intervals 0 to 80 ky and 130 to 134 ky (*Methods* and *SI Appendix*, Fig. S5) constrained by analysis of trace gases and geochemically identified volcanic layers exposed across the transect, which have been Bayesian age modeled against the recently compiled continuous 156-ky global greenhouse gas time series (CO_2_, CH_4_, and N_2_O) (27) on the AICC2012 age scale (28) (Fig. 1*B* and *Methods*). The record is located 50 km inland from the modern grounding line of the Filchner–Ronne Ice Shelf in the WSE (29) and close to the Rutford Ice Stream, one of the largest methane hydrate reserves identified in Antarctica [total organic carbon estimated to be 21,000 Gt (30), equivalent to ∼2,000 y of the current carbon emission rate of 10 GtC/year (https://www.co2.earth/global-co2-emissions)]. Today, precipitation at the site is delivered via storms originating from the South Atlantic or Weddell Sea (31). Crucially, the Ellsworth Mountains also lie in a sector of the continent that is highly responsive to isostatic rebound under a scenario of substantial WAIS mass loss, potentially preserving ice from around the time of the LIG in small valley glaciers and higher ground areas (32).

## The Patriot Hills Record

**Fig. 2.**
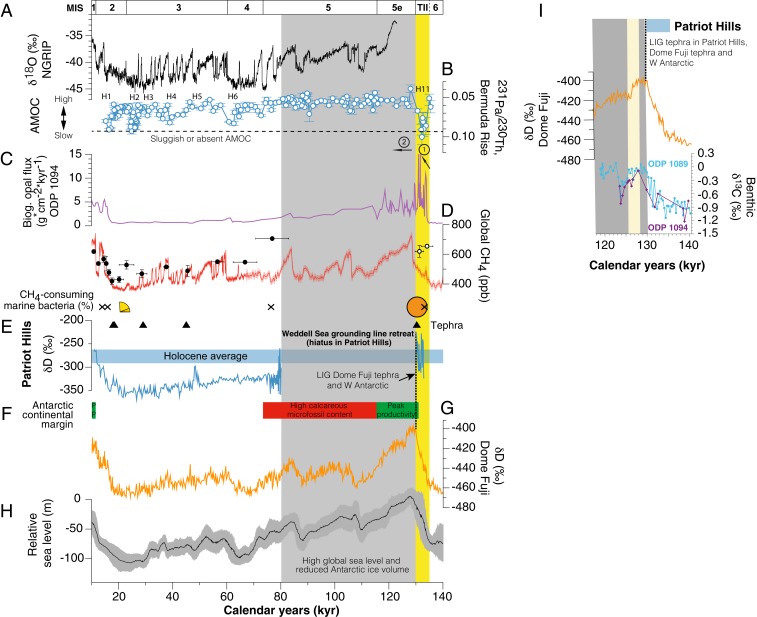
Climate, ocean circulation, and sea level changes over the past 140 ky. (*A*) δ^18^O record from the North Greenland (NGRIP) ice core (106, 107). (*B*) Bermuda Rise ^231^Pa/^230^Th data (reversed axis; 1σ uncertainty) with dashed horizontal line denoting production ratio of 0.093 marking sluggish/absent AMOC (42). Selected North Atlantic Heinrich (H) events and reduced AMOC shown. (*C*) Biogenic opal flux from ODP Site 1094 (53.2°S) as a measure of wind-driven upwelling in the Southern Ocean (45). (*D*) Comparison between the recently compiled global atmospheric methane time series (red line; 2σ envelope) (27) with the methane record from the West Antarctic Patriot Hills (black circles with 1σ uncertainty; open circles mark anomalously high-concentration data excluded from age model; *Methods*). (*E*) The Patriot Hills record. Pie chart representation (circle and segments) of percentage methane-utilizing bacteria in 16S rRNA samples from Patriot Hills; crosses denote absence of these bacteria (*Methods*). Triangles denote the presence of geochemically identified tephra layers in the Patriot Hills transect, with δD (and mean Holocene; blue envelope 1σ) values. The gray shading denotes the timing of the surface elevation change across the WSE as indicated by the hiatus in the Patriot Hills sequence and inferred substantial Antarctic ice mass loss, consistent with the reported divergence of the isotopic signal observed between the horizontal Mount Moulton ice core record from the WAIS and East Antarctic ice cores (16, 17, 33), and peak global sea level (10). (*F*) Temporal changes in ocean productivity with peak productivity (PP) (green shading) during interglacials and subsequent enhanced content of calcareous microfossils in Antarctic continental margin sediments (red shading) (34). The dashed black line shows position of tephra identified in the Patriot Hills (−340 m), Dome Fuji (1,785.14 m), and Tephra B in marine sediments from the West Antarctic continental margin. (*G*) East Antarctic Dome Fuji δ^18^O record (28, 33). (*H*) Reconstructed relative sea level curve with 2σ envelope (10). The yellow shading highlights the timing of iceberg-rafted Heinrich debris event 11 (H11), when large amounts of iceberg-rafted debris were deposited in the North Atlantic (43) and the ^231^Pa/^230^Th ratio on Bermuda Rise shifted toward the production ratio of 0.093, representative of sluggish or absent AMOC (42); the circled numbers 1 and 2 denote enhanced upwelling-induced warming in the Southern Ocean and Antarctic ice mass loss, respectively. (*I*) Close-up of Termination II and the onset of the LIG highlighting the high-precision correlation enabled by the Patriot Hills tephra (∼130 ky) and the carbon isotopic composition of benthic foraminifera from ODP Site 1089 and ODP Site 1094 (46) (Fig. 1*A*). The cream shading highlights the inferred collapse of the AABW reported from ODP 1094 (46). Dashed vertical line denotes LIG tephra in Patriot Hills, Dome Fuji, and West Antarctic continental margin.

**Fig. 3.**
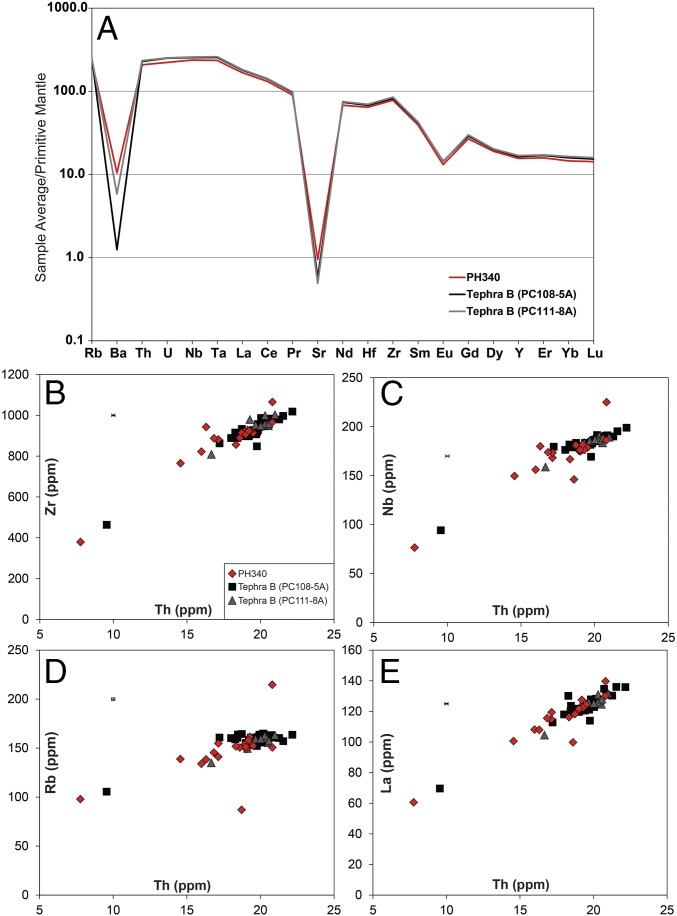
Average trace element concentrations of Patriot Hills tephra at −340 m and Tephra B from marine sediment cores PC108 (4.65-m depth) and PC111 (6.86-m depth) (34) normalized to Primitive Mantle (108). (*A*) Biplots show comparison between selected trace element concentrations of the tephra in the different sequences. Error bars on plots show 2σ of replicate analyses of MPI-DING StHs6/80-G (87), but errors are typically smaller than the data symbols (*B*–*E*).

The isotopic series of δD across the Patriot Hills BIA exhibits a coherent record of relatively low values between 18 and 80 ky, consistent with a glacial-age sequence (Fig. 2*E*). Below these layers and at the periphery of zones of higher ice flow (29), we find an older unit of ice exposed at the surface expressed by a step change to enriched (interglacial) isotopic values (Fig. 2*E* and *SI Appendix*, Fig. S7), implying proximal warmer conditions and reduced sea ice extent (33). Importantly, we identify a distinct tephra horizon near the boundary of this older unit of ice, which, based on major and trace element geochemical fingerprinting (Fig. 3 and *SI Appendix*, Fig. S11), is correlated to a volcanic ash from the penultimate deglaciation (Termination II) referred to as Tephra B in marine sediments on the West Antarctic continental margin (34) and identified at 1,785.14-m depth in the Dome Fuji ice core, where it is dated to 130.7 ± 1.8 ky (AICC2012 timescale) (28, 33, 34). The start of the oldest section of the sequence is dated here to 134.1 ± 2.2 ky, consistent with modeling studies, airborne radio-echo sounding lines, and GPR profiles, which imply older ice exists at depth in the Ellsworth Mountains (29, 32) (Fig. 1 *B*–*E*).

The combined tephra and trace gas analyses suggest a ∼50-ky hiatus after Termination II (130.1 ± 1.8 ky). Radio-echo sounding surveys across the WSE have identified a large subglacial basin comprising landforms reflecting restricted, dynamic, marine-proximal alpine glaciation, with hanging tributary valleys feeding an overdeepened Ellsworth Trough (35). The extensive nature of the subglacial features implies substantial and repeated mass loss of the marine sections of the WAIS (presumably through the Pleistocene), with the ice margin some 200 km inland of present day (35). However, the timing of most recent retreat is currently unknown. While previous surface exposure dating in the region has suggested that the WAIS contribution to global sea level rise during warmer periods was limited to 3.3 m above present (36), relatively short-duration interglacial periods may have resulted in near-complete deglaciation (35). Previous work has interpreted erosional features D1 and D2 in the Patriot Hills BIA to be a consequence of extensive ice surface lowering in Horseshoe Valley (up to ∼500 m since the Last Glacial Maximum, 21 ky) and more exposure of katabatic-enhancing nunataks, resulting in increased wind scour (26, 37). While this scenario may explain unconformity D0, previous work has demonstrated Horseshoe Valley and the wider WSE to be highly sensitive to periods of ice stream advance or retreat in the last glacial cycle and Holocene, with dramatic reductions in surface elevation (26, 37–39), changes that may result in more than just increased wind scour. Importantly, the head of Horseshoe Valley is an overdeepened trough (down to ∼2,000 m below sea level), while toward the mouth of the valley, a subglacial ridge is found at ∼200 m below current sea level with an ice thickness of some 750 m (*Methods* and *SI Appendix*, Fig. S3), allowing the isolation and stagnation of ice in Horseshoe Valley over multiple millennia. Furthermore, glaciological investigations assessing the impact of ice shelf loss on glaciers along the Antarctic Peninsula provide important insights into the preservation of ice, albeit on a smaller scale. The 2002 Larsen B ice shelf collapse led to many of the tributary glaciers abruptly changing from a convex to a concave profile (cross-section) (40), with relict ice left isolated on the upper flanks of the valleys (41). These scenarios are consistent with extensive grounding line retreat across the inner shelf of the Weddell Sea and associated substantial ice loss across the wider WSE (29).

The ice at Patriot Hills therefore appears to preserve a record of glacier flow in Horseshoe Valley up to the moment when the Filcher–Ronne Ice Shelf collapsed, after which the sequence remained isolated due to regional ice flow reconfiguration for multiple millennia; a situation that persisted until the ice surface had risen sufficiently to enable the regional ice flow to recover sometime during late MIS 5. We cannot, however, discount the possibility that there were one or more cycles of ice mass gain and loss through MIS 5. The presence of a discrete older ice unit along the flanks of the Ellsworth Mountains (29) (Fig. 1 and *SI Appendix*, Fig. S2) and the subsequent inferred highly variable climate and/or sea ice extent across the wider WSE (*SI Appendix*, Figs. S7 and S13) imply the preservation of ice from MIS 6/5 (Termination II) and 5/4 transitions in Horseshoe Valley. Our data provide evidence for substantial mass loss across the WSE during the LIG.

## Ocean Warming

**Fig. 4.**
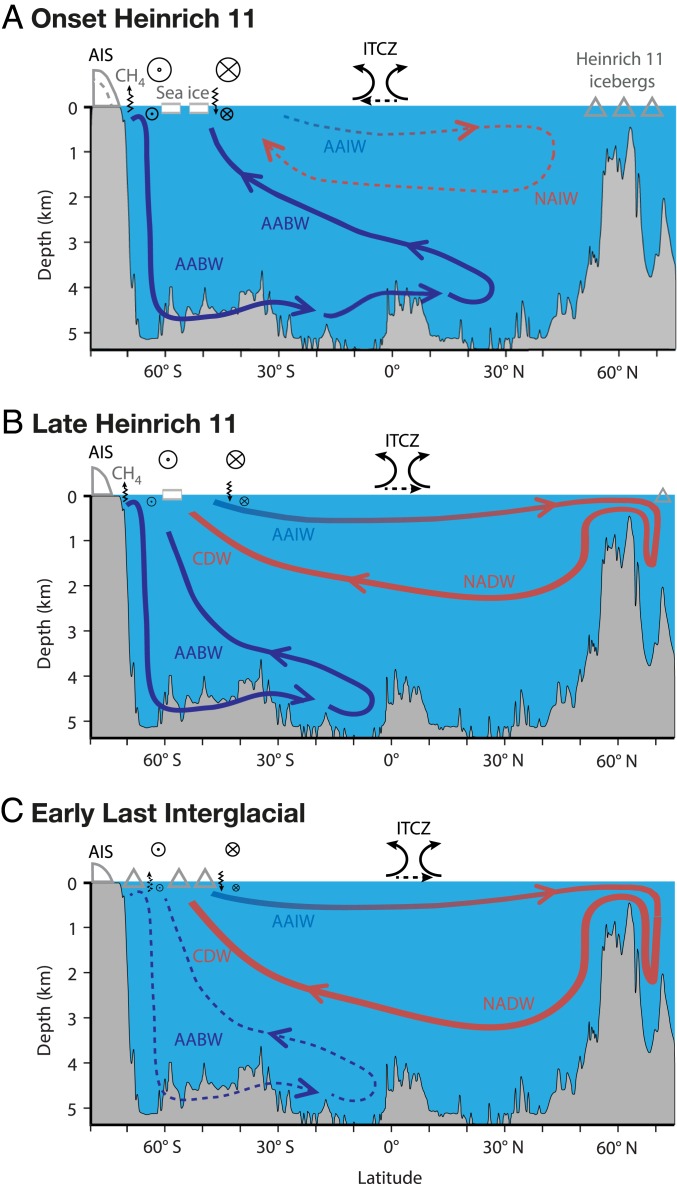
Ocean–atmospheric interactions during Termination II and the LIG. Panels show changing Atlantic meridional overturning circulation (AMOC) in response to iceberg discharge (*A* and *B*) in the North Atlantic (Heinrich event 11) during Termination II and (*C*) from the Antarctic Ice Sheet (AIS) during the LIG, with inferred shifts in atmospheric circulation including midlatitude Southern Hemisphere westerly (crossed circle) airflow and Intertropical Convergence Zone (ITCZ) (14, 43, 45, 46, 48). The vertical arrows denote CH_4_ and heat flux associated with Antarctic coastal easterly (dot in circle) and westerly (crossed circle) airflow (30, 47). AABW, AAIW, CDW, NAIW, and NADW define Antarctic Bottom Water, Antarctic Intermediate Water, Circumpolar Deep Water, North Atlantic Intermediate Water, and North Atlantic Deep Water, respectively.

What could be the cause of this ice loss in the South Atlantic sector of the Southern Ocean? Recent work has proposed that the iceberg-rafted Heinrich 11 event between 135 and 130 ky (during Termination II) may have significantly reduced North Atlantic Deep Water (NADW) formation and shut down the Atlantic meridional overturning circulation (AMOC) (42), resulting in net heat accumulation in the Southern Hemisphere (the bipolar seesaw pattern of northern cooling and southern warming) (43, 44) (Fig. 4*A*). Under this scenario, surface cooling during Heinrich 11 increased the northern latitudinal temperature gradient and caused a southward migration of the Intertropical Convergence Zone and midlatitude Southern Hemisphere westerly airflow (14, 45). Importantly, Heinrich 11 was probably one of the largest of the iceberg-rafting events over the last 140 ky (including H-1 and H-2) and during a time of likely weakened AMOC (42). In the Southern Ocean, the associated northward Ekman transport of cool surface waters (something akin to today; Fig. 1*A*) was likely compensated by increased delivery of relatively warm and nutrient-rich Circumpolar Deep Water (CDW) toward the Antarctic margin (14, 34, 43, 45, 46), potentially leading to enhanced thermal erosion of ice at exposed grounding lines (43, 47). This interpretation is supported by the enriched benthic foraminifera ^13^C values into the LIG (46), a proxy for the influence of NADW on CDW in the south, implying northern (warmer) waters were reaching far south for much of this period (and a cause of persistent loss of ice volume) (Fig. 2*I*). The unambiguous precise correlation between the Patriot Hills ice and West Antarctic marine records (34) afforded by the Termination II tephra demonstrates that the warming recorded in the BIA is coincident with a major, well-documented peak in marine temperatures and productivity around the Antarctic continent and in the Southern Ocean (34, 45, 46) (Fig. 2). The subsequent delivery of large volumes of associated freshwater into the Southern Ocean during the LIG would have reduced Antarctic Bottom Water (AABW) production (46), resulting in increased deepwater formation in the North Atlantic (43, 48, 49) (Fig. 4*C*). Recent modeling results suggest that increased heat transport beneath the ice shelves can drive extensive grounding-line retreat, triggering substantial drawdown of the Antarctic ice sheet (2, 14, 20) (Fig. 4*B*). Of concern, warming of the ocean cavity in the WSE is projected to increase during the 21st century (50).

With Southern Ocean warming and concurrent ice sheet retreat, the large methane reservoirs in Antarctic sedimentary basins (e.g., Rutford Ice Stream) could have become vulnerable to release (30) and may have contributed to elevated atmospheric levels through the LIG (8, 27) (Fig. 2*D*). High-latitude open water and sea ice are rich in microbial communities, components of which may be collected by passing storms and delivered onto the ice sheet (e.g., prokaryotes, DNA), offering insights into offshore environmental processes (51, 52). To investigate environmental changes prior to and after the ice sheet reconfiguration recorded in the Patriot Hills BIA, we applied an established ancient DNA methodology and sequencing to provide a description of ancient microbial species preserved within the ice (*Methods*). Methane-utilizing microorganisms were found in three samples along the Patriot Hills transect and were absent from other samples on the transect and laboratory controls. While such microbes are not obligate methylotrophs and can be present in nonmethane-dominated environments (53), they would be expected to be at very different abundances to what we find. The most striking feature of the Patriot Hills BIA genetic record was detected immediately prior to inferred ice loss, where *Methyloversatilis* microbes dominated the detectable microbial diversity (∼130 ky) (Fig. 2*E* and *SI Appendix*, Fig. S15). *Methyloversatilis* was only found in high abundance in this sample (with trace amounts identified at ∼22 ky). Crucially, *Methyloversatilis* are facultative methylotrophs and live on single and multicarbon sources (54), consistent with elevated levels of CH_4_ and active methane oxidation by *Methyloversatilis* or other methanotrophic taxa in marine sediments or in the water column during the end of Termination II (*SI Appendix*). More work is needed to explore the potential for microbial methane utilization in this unique environment.

## Antarctic Ice Sheet Modeling

**Fig. 5.**
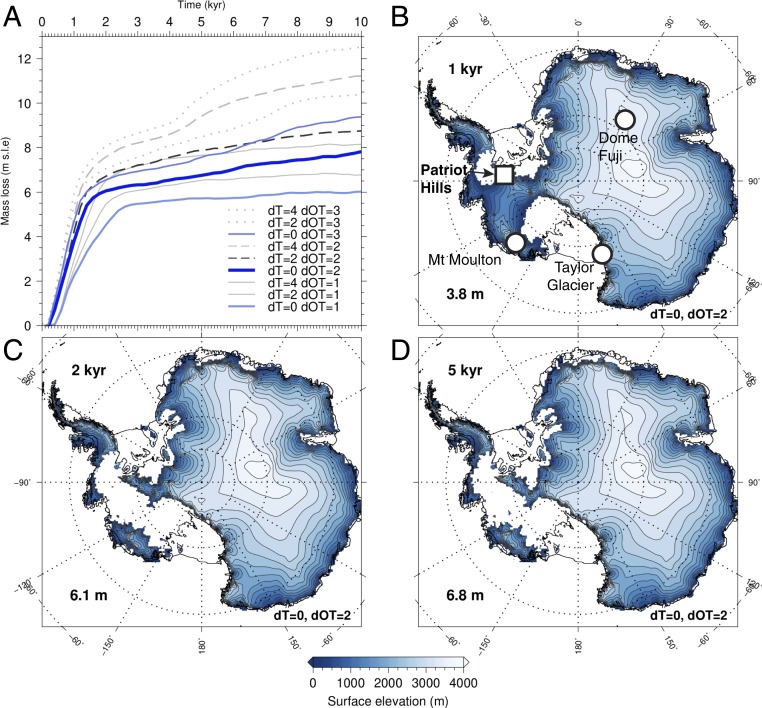
Modeled Antarctic ice sheet evolution under idealized forcing scenarios consistent with range of inferred LIG temperatures. (*A*) Sea level equivalent mass loss for ice sheet simulations forced by a range of air and ocean temperature anomalies relative to present day. “dT” and “dOT” describe atmospheric and ocean temperature anomalies, respectively. *B*–*D* show Antarctic Ice Sheet extent and elevation with 2 °C warmer ocean temperatures over time intervals of 1, 2, and 5 ky, respectively (with no atmospheric warming); equivalent sea level contribution is given in the *Bottom Left* corner of each panel. Locations of Patriot Hills (Ellsworth Mountains, WAIS) and ice core records discussed in this study are shown in *B*. *Inset* box in *B* outlines region shown in Fig. 6.

The inferred substantial mass loss across the WSE implies a major role for ocean warming during Termination II and the LIG. To provide a framework for interpreting ice sheet dynamics around the Patriot Hills and across Antarctica, we present a series of temperature sensitivity experiments using the Parallel Ice Sheet Model, version 0.6.3 (Fig. 5) (2). We report here nine different simulations that capture a range of ocean and atmospheric warming scenarios (0° to 3 °C). Importantly, the most comprehensive published high-latitude (≥40° S) network of quantified sea surface temperature (SST) estimates suggests an early LIG (∼130 ky) warming of 1.6 ± 0.9 °C relative to present day (9, 23), providing an upper limit on the sensitivity of the Antarctic ice sheet to ocean temperatures. The pattern of circum-Antarctic ocean warming during this time period is not well established so we assume a spatially uniform warming pattern relative to present day temperatures. Our model time series illustrates that the majority of ice loss takes place within the first two millennia, depending on the magnitude of the forcing (Fig. 5 and Table 1). This corresponds to the time period of inferred loss of marine-based sectors of the ice sheet (Fig. 2), primarily in West Antarctica. In contrast to some whole-continent models, our simulations do not include mechanisms by which a grounded ice cliff may collapse (3), a process that produces considerably faster and greater ice margin retreat than reported here.

**Table 1.** Sea level equivalent mass loss (meters) for Antarctic ice sheet simulations forced over 10,000 y by range of annual air and ocean temperature anomalies relative to present day

	1,000 y	2,000 y	5,000 y	10,000 y
1 °C SST warming				
0 °C air	2.2	4.5	5.7	6.0
2 °C air	2.5	5.5	6.5	6.8
4 °C air	2.9	6.5	7.7	8.2
2 °C SST warming				
0 °C air	3.8	6.1	6.8	7.8
2 °C air	4.2	6.7	7.9	8.8
4 °C air	4.8	7.6	9.4	11.2
3 °C SST warming				
0 °C air	4.7	6.6	7.6	9.4
2 °C air	5.4	7.1	8.5	10.4
4 °C air	5.9	8.1	10.3	12.5

Note: The temperatures applied were applied linearly over the first 1,000 y.

**Fig. 6.**
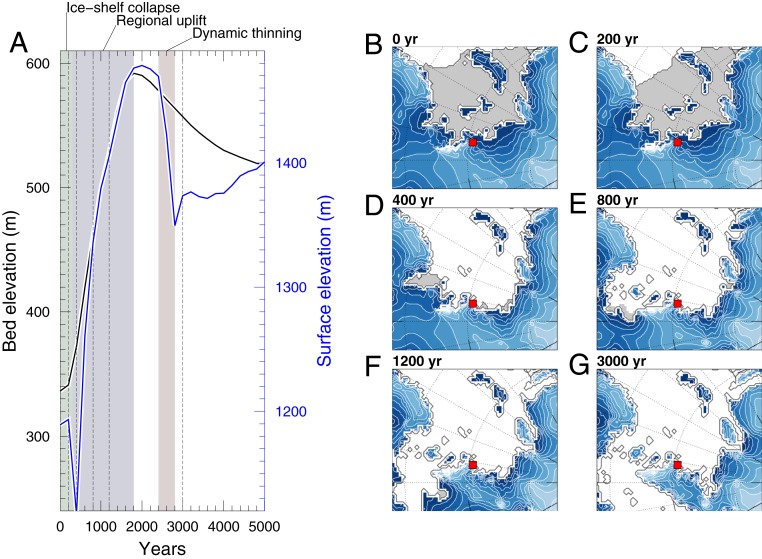
Bed (black line) and surface (blue) elevation changes at Patriot Hills (Ellsworth Mountains, WAIS) in response to 2 °C warmer ocean temperatures over a time interval of 5 ky (with no atmospheric warming) (*A*). Bed (black line) and surface (blue) elevation changes vs. time, with phases of the prevalence of particular processes, such as ice shelf collapse (mint shaded), regional uplift (gray shaded), and dynamic thinning (light-brown shaded), highlighted. (*B*–*G*) Selected time slices corresponding to dashed lines in *A* showing ice shelf extent and ice sheet elevation in the Weddell Sea Embayment (WSE) over the first 3 ky. Location of Patriot Hills is marked by the red square; the gray shaded areas are ice shelf covered, while the white areas are free of both grounded and floating glacial ice.

For the 2 °C warmer than present day ocean temperature scenario (comparable to reconstructed estimates) (9, 23), with no additional atmospheric warming, our model predicts a contribution to GMSL rise of 3.8 m in the first millennium of forcing (Fig. 5*B*). The loss of the Filchner–Ronne Ice Shelf within 200 y of warming triggers a nonlinear response by removing the buttressing force that stabilizes grounded ice across large parts of the WSE and the EAIS (most notably the Recovery Basin) (Fig. 6 and *SI Appendix*, Fig. S17). Ongoing slower ice loss subsequently occurs around the margins of East Antarctica, producing a sustained contribution to sea level rise. Even for relatively cool ocean-forced runs, we find the shelves collapse quickly between the 200-y intervals (*SI Appendix*, Fig. S18). Indeed, during the warmer ocean model runs, the shelves disappear too quickly to observe the relevant processes on the timescale covered by the snapshots. For instance, under the scenario of 2 °C linear warming, the ice shelves disappear within 600 y of forcing (when temperatures reached between +0.4 and +0.8 °C). Other modeling studies using a range of different setups have reported similar rapid losses of the ice shelves during the onset of the LIG (24, 25). Our results are therefore consistent with an increasing body of evidence that the stability of Antarctic ice shelves is vulnerable to a relatively low temperature threshold (2, 24, 25).

Recent work has suggested that the Ellsworth Mountains would have experienced a relatively large positive isostatic adjustment (>200 m) accompanying the loss of the WAIS (24, 25, 32), although the model outputs may be underestimated (25). To investigate how an evolving ice sheet geometry would manifest across the wider region, we extracted local ice surface and bed elevations for the WSE from the model simulation that uses a preindustrial ice sheet configuration with 2 °C ocean warming and no atmospheric warming. Fig. 6 *A*–*G* illustrates the sequence of events that take place as the ice sheet evolves. First, loss of the Filchner–Ronne Ice Shelf in the Weddell Sea triggers a nonlinear response, removing the buttressing force that stabilizes grounded ice across large parts of the WSE and the EAIS (most notably the Recovery Basin) (55). The loss of back-stress allows for an acceleration of grounded ice and a rapid but short-lived thinning episode (32). At the Patriot Hills, bedrock uplift of ∼30 m over this 0.2-ky period is outpaced by a surface lowering of ∼75 m, implying a net ice sheet thinning of around 105 m. Subsequently, regional-scale isostatic uplift elevates both the bed topography (∼250 m) and ice sheet surface (∼350 m) relative to the initial configuration. The difference between these two values reflects positive net mass balance of the ice sheet here (∼0.055 m/y). After around 2.5 ky, renewed dynamic thinning of the ice sheet in the Patriot Hills leads to a rapid thinning and lowering of the ice sheet surface, at a rate exceeding regional-scale bedrock subsidence (120 m over 0.4 ky, or 0.3 m/y, compared to ∼70 m over 3.2 ky, or 0.022 m/y, respectively) (Fig. 6). For the 1 and 3 °C warming scenarios, similar spatial losses are modeled, with GMSL rises of 2.2 and 4.7 m for the first millennium, respectively (Table 1). Atmospheric warming of the magnitude suggested by Antarctic cores (>4 °C) (16, 17, 56–58) adds an additional meter of equivalent global sea level within the first millennium (*SI Appendix*, Fig. S19).

Previous work has highlighted the sensitivities of the Ronne–Filchner and Ross ice shelves to warming under a range of model setups (3, 18). Recently published transient ice sheet model simulations covering the last glacial–interglacial cycle have investigated a range of scenarios encompassing different geothermal heat fluxes, ice shelf calving heights, mantle viscosity values, temperature and sea level forcing scenarios, etc. (24, 25). Importantly, these studies recognize the loss of the WAIS forced by warming across what is relatively narrow LIG temperature peak, with a maximum bedrock elevation of ∼400 to 650 m, and surface elevation changes of >1,500 m, larger than that reported here. However, it is important to note that these relatively large estimates are likely influenced by the glacial loading that was experienced during MIS 6.

The Patriot Hills record is consistent with basin-scale mass loss early in the LIG (15, 32) as a consequence of regional ice dynamic changes and isostatically driven isolation of Horseshoe Valley from sustained ocean forcing. While some modeling studies have argued the loss of the Filchner–Ronne Ice Shelf does not display a strong marine ice sheet instability feedback (59) and that isostatically driven rebound may halt ice retreat (18), our results suggest otherwise. Indeed, recent work has proposed that if mass loss comparable to recent decades is maintained for as little as 60 y, the WAIS could be irrevocably destabilized over subsequent millennia through the collapse in the Amundsen Sea sector, overcoming any isostatically driven rebound (60). Future work will be required to undertake large ensembles of high-resolution ice sheet model simulations that capture the full range of ice dynamics, ice–ocean–atmosphere coupling, MIS 6 ice sheet configuration, and spatial and temporal temperature evolution across this period to fully capture the uncertainty associated with LIG mass loss. However, we consider our ice sheet modeling simulations to be comparable to previous studies (24, 25) in the magnitude of rate of change and mass loss, and support the interpretation of the Patriot Hill BIA record. Our results suggest substantial ice sheet mass loss and flow reconfiguration in response to ocean warming, outpacing any bedrock rebound that might have stabilized the ice sheet (Fig. 6). Furthermore, marine-based ice sheets are particularly vulnerable to hysteresis effects (61), which could explain the 50-ky hiatus in the Patriot Hills blue ice record, particularly given the relatively low modeled temperature threshold (0.5 to 0.7 °C ocean warming) for ice shelf loss (*SI Appendix*, Fig. S17).

The evidence for substantial mass loss from Antarctica in the early LIG has important implications for the future (4, 62). Our field-based reconstruction and modeling results support a growing body of evidence that the Antarctic ice sheet is highly sensitive to ocean temperatures. Driven by enhanced basal melt through increased heat transport into cavities beneath the ice shelves (2, 47), this process is projected to increase with a weakening AMOC during the 21st century (50, 63–65), which may lead to other positive feedbacks such as destabilization of methane hydrate reserves (30).

## Methods

### Patriot Hills.

#### Site description and geomorphological context.

The Patriot Hills BIA (Horseshoe Valley, Ellsworth Mountains; 80°18′S, 81°21′W) is a slow flowing (<12 m⋅y^−1^) compound glacier system situated within an overdeepened catchment that coalesces with the Institute Ice Stream at the periphery of the WSE (29, 37, 66–68) (Fig. 1 and *SI Appendix*, Figs. S1–S4). Airborne radio-echo sounding surveys across the Ellsworth Mountains have revealed several wide (up to 34 km across) and long (260 km) subglacial troughs containing ice up to 2,620 m thick (Fig. 1) (29), along the side of which, two radar zones have been interpreted to indicate layers of ice with contrasting physical properties, consistent with snow deposited during previous glacial/interglacial transitions. In contrast to the other troughs across the Ellsworth Mountains, contemporary ice within the Horseshoe Valley Trough maintains the slowest average flow speeds of all, at 12 m⋅a^−1^ (cf. the main trunk of the Institute Ice Stream reaches speeds up to 415 m⋅a^−1^). This is in large part due to the configuration of the Horseshoe Valley Trough where the ice thickness measures in excess of 2,000 m at the head of the valley and reduces to ∼1,400 m downstream; toward the mouth of the valley, a subglacial ridge is found at ∼200 m below sea level with the ice thickness some 750 m thick (*SI Appendix*, Fig. S3) (69). The new Digital Elevation Model data for the WSE is available at https://data.bas.ac.uk/full-record.php?id=GB/NERC/BAS/PDC/00937. The configuration of the bed and resulting slow flow in Horseshoe Valley has two major benefits for our study. It allows 1) a long record of ice to accumulate, and 2) the isolation and preservation of ice during periods of regional and Antarctic-wide mass loss.

In the lee of a small mountain chain at the end of Horseshoe Valley called Patriot Hills, strong local katabatic winds descend into the valley from the polar plateau, ablating the ice sheet surface by up to 170 kg⋅m^−2^⋅y^−1^ (68). As a result, ancient ice is drawn up from depth in the Horseshoe Valley Trough to form an extensive BIA (more than 1,150 m across; *SI Appendix*, Fig. S4) (31, 37, 38). High-resolution analysis using GPR (37) and isotopes identifies three distinct unconformities [surface distances relative to an arbitrary transect datum (31) set at zero]: 247 m (D1), 360 m (D2), and −339 m (D0). Based on the trace gas, tephra, and isotopic values of the surface ice beyond D0 (closest to Patriot Hills), we interpret this section of the record to be Termination II in age (see below). No glaciomarine sediments have been identified at any of the boundaries.

Previous work has interpreted erosional features D1 and D2 in the Patriot Hills BIA to be a consequence of extensive ice surface lowering in Horseshoe Valley (up to ∼500 m since the Last Glacial Maximum, 21 ky) and more exposure of katabatic-enhancing nunataks, resulting in increased wind scour (26, 37). While this scenario may explain unconformity D0, other studies have demonstrated Horseshoe Valley and the wider WSE to be highly sensitive to periods of rapid ice stream advance or retreat in the last glacial cycle and Holocene with dramatic reductions in surface elevation (26, 37–39). Recent work investigating the impact of ice shelf loss on glaciers along the Antarctic Peninsula provides important insights, albeit on a smaller scale. The 2002 Larsen B ice shelf collapse led to many of the tributary glaciers abruptly changing from a convex to a concave profile (40), with relict ice left isolated on the upper flanks of the valleys (41). Under a scenario of extreme ice surface lowering arising from ocean warming during the early LIG, the ice at Patriot Hills preserves a record of glacier flow in the overdeepened Horseshoe Valley up to the moment when the Filcher–Ronne Ice Shelf collapsed, after which the sequence likely remained isolated for multiple millennia until the ice surface had risen sufficiently to reincorporate the isolated ice into the glacier sometime during late MIS 5. The relatively enriched deuterium and ^18^O stable isotope values, ancient DNA (notably the detection of *Methyloversatilis* microbes in the sample form −340 m in the Patriot Hills record), and ice sheet modeling are consistent with early offshore warming in the south Atlantic and substantial ice mass loss in the early LIG (34, 46, 62), preserving most (if not all) of the Termination II ice record during the period represented by the D0 unconformity (see below). We therefore consider D0 reflects a significant fall in surface elevation and change in flow direction due to isostatically driven isolation of the valley during a period of rapid drawdown of the ice streams across the WSE.

#### Chronology.

Chronological control across the transect is provided by a comprehensive suite of trace gas samples—carbon dioxide (CO_2_), methane (CH_4_) and nitrous oxides (N_2_O)—and volcanic tephra horizons. The trace gas measurements provide a range of possible age solutions against the recently published 156-ky smoothed global time series for these gas species (27), which together with the absolute constraints provided by the tephra horizons, allows the development of a robust chronological framework that can be tied directly to the isotopic series through high-resolution GPR (31, 37) (*SI Appendix*, Figs. S6 and S7). A Kovacs 9-cm-diameter ice corer was used to collect ice for gas and taken from >3-m depth to minimize modern air contamination and/or alteration (31). The samples were double bagged and sealed in the field, and transported frozen to the Commonwealth Scientific and Industrial Research Organisation (CSIRO) ICELAB facility in Melbourne for the extraction and measurement of trace gases using a modified dry extraction “cheese grater” and cryogenic trapping technique (70, 71). The trapped air samples were analyzed by gas chromatography, and the trace gas concentrations are reported against the calibration scales maintained by CSIRO GASLAB (72). Where sufficient material was available, duplicates were analyzed.

The presence of visible tephra layers (volcanic ash horizons) provides additional chronological control for the Patriot Hills BIA. Here, we report two new tephras from Patriot Hills at 10 and −340 m, both observed as ∼4-cm units of dispersed shards (*SI Appendix*, Fig. S9). Shards were extracted by centrifugation of the melted ice samples and put onto a glass slide for electron microprobe analysis. The slides were ground and polished using silica carbide paper and decreasing grades of diamond suspension to expose fresh sections of glass. Single-grain analyses of 10 oxides were performed on a Cameca SX-100 electron microprobe at the Tephrochronology Analytical Unit, University of Edinburgh. See *SI Appendix* for operating conditions (73); geochemical results are provided in *SI Appendix*, Table S1. The shards from 10 m are bimodal, with a basanitic and trachytic composition (*SI Appendix*, Fig. S10). The shards from −340 m are trachytic in composition and exhibit a tightly clustered population (*SI Appendix*, Fig. S11). Both were compared to published tephras from across Antarctica (34, 74–84). The 10-m tephra has the closest match to be the basanite Tephra C from the WAIS Divide at 3,149.12 m (Similarity Coefficient or SC = 0.98), equivalent to 44.9 ± 0.3 ky (84). The −340-m tephra revealed the closest match to a tephra layer in the Dome Fuji ice core at 1,785.14-m depth [SC = 0.966; equivalent to 130.7 ± 1.8 ky on the AICC2012 timescale (28, 77, 85); data previously unpublished].

A widespread tephra found in marine sedimentary records on the West Antarctic continental margin (Tephra B) has been proposed to correlate to the tephra at Dome Fuji 1,785.14 m, but the correlation has until now remained only tentative in the absence of any reported geochemistry from the latter (34). Here, we find the major oxides from Tephra B have a close match to Patriot Hills −340 m (SC = 0.948), consistent with this interpretation. To test this correlation, we undertook trace element analysis of the glass shards from Patriot Hills at −340 m. Unfortunately, the Dome Fuji shards were too thin for analysis. However, we were able to undertake trace element analyses on Tephra B samples from two marine sediment cores from the West Antarctic continental margin: PC108 (4.65-m depth) and PC111 (6.86-m depth) (34). Trace element analysis of volcanic glass shards were performed using an Agilent 8900 triple-quadrupole inductively coupled plasma mass spectrometry (ICP-MS) (ICP-QQQ) coupled to a Resonetics 193-nm ArF excimer laser ablation in the Department of Earth Sciences, Royal Holloway, University of London. See *SI Appendix* for operating conditions (86). Accuracies of laser ablation ICP-MS analyses of ATHO-G and reference StHs6/80-G MPI-DING (87) glass were typically ≤5%. Identical trace element glass chemistries (Fig. 2 and *SI Appendix*, Table S2) strongly support the correlation of Patriot Hills −340-m tephra horizon and the marine West Antarctic Tephra B (34), which is in turn correlated to Dome Fuji 1,785.14 m (33, 34, 77, 85), and probably originates from the Marie Byrd Land volcanic province (West Antarctica) (34). The recognition of a widespread tephra horizon across a large sector of the Antarctic at the very onset of the LIG provides a time-parallel marker horizon crucial for future studies investigating Antarctic ice sheet mass loss.

To develop an age model, we undertook Bayesian age modeling using a Poisson process deposition model (P_sequence) in the software package OxCal, version 4.2.4 (https://c14.arch.ox.ac.uk/) (*SI Appendix*, Tables S3 and S4) (88, 89). Using Bayes theorem, the algorithms employed sample possible solutions with a probability that is the product of the prior and likelihood probabilities (90, 91). “Calibration curves” with 20-y resolution were developed for the three trace gas species using the 156-ky time series (27). Taking into account the deposition model, the reported ages of the tephra layers, and the common age solutions offered by the trace gas measurements, the posterior probability densities quantify the most probable age distributions. The available constraints suggest the 1,156-m-long Patriot Hills BIA transect spans time intervals from ∼134.2 to ∼1.3 ky comprising four key zones: 4 (−362 to −339 m, equivalent to 134.2 ± 2.2 to 130.1 ± 1.8 ky), 3 (−326 to 240 m, equivalent to 80 ± 6.1 to 22.7 ± 2.8 ky), 2 (240 to 360 m, equivalent to 22.7 ± 2.8 to 10.3 ± 0.4 ky), and 1 (360 to 800 m, 10.3 ± 0.4 to 1.3 ± 0.6 ky). The Agreement Index (a measure of the agreement between the model—prior—and the observational data—likelihood) for the Patriot Hills age model was 101.6% (*A*_overall_ = 71.2%), exceeding the recommended rejection Agreement Index threshold of 60% (89) (*Methods*). Regardless of the relatively large uncertainty associated with the oldest section of ice (zone 4), the identification of the 130.7 ± 1.8 ky (AICC2012 timescale) Tephra B/Dome Fuji 1,785.14 m (28, 33, 34) within Patriot Hills at −340 m unambiguously demonstrates the presence of Termination II-age ice. Future age constraints will inevitably help improve the accuracy and precision of the age model.

#### Isotopes.

δD and δ^18^O isotopic measurements were performed between 1- and 3-m resolution at James Cook University using diffusion sampling–cavity ring-down spectrometry (International Atomic Energy WICO Laboratory ID 16139) (92). This system continuously converts liquid water into water vapor for real-time stable isotope analysis by laser spectroscopy (Picarro L2120-i). See *SI Appendix* for operating conditions. To ensure reproducibility, a subset of samples was rerun at University of New South Wales ICELAB for δD and δ^18^O using a Los Gatos Research Liquid Water Isotope Analyzer 24 d (International Atomic Energy WICO Laboratory ID 16117). Reported overall analytical precision on long-term ice core standards is <0.32‰ for δD and <0.13 for δ^18^O values. All isotopic values are expressed relative to the Vienna Standard Mean Ocean Water 2 (VSMOW2). The isotopic datasets generated in this study are available at the publicly accessible National Oceanic and Atmospheric Administration (NOAA) Paleoclimatology Database (93) and are available upon request.

#### Ancient DNA analysis.

BIAs offer the opportunity to process large-volume samples of continental Antarctic ice in the field (∼7 kg per temporal sample), creating the prospect of generating sufficient microbial concentrations to permit detailed genetic biodiversity surveys (51, 52) (Fig. 2). To obtain the samples, a Kovac corer was thoroughly cleaned with 1 to 3% bleach and wiped with 95% ethanol between core extractions to minimize cross-contamination. After coring, the top 1 m of ice was removed and discarded, before 1- to 2-m-long cores were collected in 50-cm sections and immediately placed into clean PFTE flexible plastic tubing. A heat sealer was used to close the tubing at the top and bottom of the core. The sealed core was then cut from the remaining tubing with a sterile blade, and the process was repeated to encase the core in a second layer of the plastic tubing for protection during transport. Within 1 to 6 h of extraction, the tubing-encased BIA cores were hung inside a large dome tent to melt via solar radiation over 12 to 24 h, using black plastic bin liners around the plastic tubing to speed up the process where necessary. The melted BIA sample was transferred from the inside layer of tubing directly into a hand-powered vacuum filtration system cleaned with 1 to 3% bleach and ethanol wipes between samples. For each sample, disposable, sterile, 0.45-µm nitrocellulose filters were used to filter and collect whole bacterial organisms trapped in the ice during its formation, and reduce noise caused by environmental DNA. Filters were stored in sterile plastic bags, frozen at −20 °C, and returned to the Australian Centre for Ancient DNA in Adelaide for ultraclean genetic analysis.

Strict ancient DNA methodologies designed to assess low-biomass microbial samples were applied (94) (see *SI Appendix* for detailed methodology and analysis). DNA from all ice samples as well as extensive sampling and laboratory controls were extracted using two methods to maximize species recovery, and 16S ribosomal RNA libraries were amplified in triplicate using published, universal bacterial and archaeal 16S ribosomal RNA (rRNA) primers. After DNA sequencing, all individually indexed 16S rRNA libraries were de-multiplexed, quality filtered, and imported into QIIME, version 1.8.0. Microbial taxa were identified by comparing sequences to the Geengenes, version 13, reference database and binning sequences with 97% similar to known species into operational taxonomic units using closed reference clustering in UCLUST. Sampling and laboratory contaminants were then filtered from ice samples, and an average of 30.8% of the reads for each sample were retained (*SI Appendix*, Table S5). Retained sequences were then pooled, and the resulting taxa present in each sample were explored as a proportion of the total filtered DNA sequencing reads. Alpha and beta diversity was explored in QIIME, and importantly, no statistically significant differences in diversity were detected across the samples. Ancient DNA sample data are available upon request. While the current sample numbers limit resolution, our study highlights the untapped potential of BIA genetic data to exploit cryosphere microbial communities to investigate glaciological and environmental change (52).

### Ice Sheet Modeling.

To investigate former ice sheet dynamics around the Patriot Hills and across Antarctica, we take a range of values for polar ocean warming (1 to 3 °C) (9, 11, 23) and employ the Parallel Ice Sheet Model (PISM), version 0.6.3 (2), an open source 3D, thermomechanical coupled ice sheet/ice shelf model. PISM employs a stress balance that superposes solutions of the shallow-ice and shallow-shelf equations, and incorporates a pseudoplastic basal substrate rheology to allow for realistic sliding over meltwater saturated sediments, a bed deformation model that simulates mantle dissipation and rebound arising from spatial changes in ice loading through time (95), and a subgrid basal traction and driving stress interpolation scheme to allow realistic grounding-line motion (96, 97). We prescribe a mantle viscosity of 1 × 10^20^ Pa⋅s, which is lower than the PISM default (1 × 10^21^) and intended to capture more accurately the weaker mantle of West Antarctica, where the majority of mass loss takes place. In the experiments presented here, we chose not to implement the subgrid scale interpolated ice shelf basal melt component of this scheme (2, 98). Calving is parameterized using horizontal strain rates and a minimum thickness criterion (220 m) (99, 100). Our experimental methodology is identical to that described in detail elsewhere (101, 102). Climate and ocean temperature perturbations are applied as spatially uniform linear increments added to boundary distributions representing present-day conditions. Linear increases take place between 2,000 and 3,000 model years. The first 2,000 y (no forcing) allow any transient behavior associated with model initialization to take place in the absence of environmental perturbations, whereas the subsequent 1,000 y force the ice sheet to evolve slowly to changes in air and ocean temperature and precipitation. All experiments are run at a spatial resolution of 20 km.

Reconstructed summer SST anomalies relative to present day (the 1998 World Ocean Atlas) (9) were used to inform on a range of warmer air and ocean LIG conditions and applied to a stable modern configuration of the Antarctic Ice Sheet to help interpret the Patriot Hills record (Table 1). A limitation of this approach is that the transient history from the preceding glacial state is not simulated. However, for the response of the ice shelves, this colder prehistory should not be critical, and the experiments as performed are directly relevant for the future of the ice sheets. From these simulations, we extract data from the first 10 ky. The ice sheet modeling outputs support the view that ocean (rather than atmosphere) warming was the primary driver of ice shelf collapse and substantial early LIG mass loss in Patriot Hills and across large parts of Antarctica (*SI Appendix*, Fig. S18). With a surface ocean warming of 2 °C, our simulations suggest isolation and stagnation of ice in Horseshoe Valley and the loss of the Bungenstock Ice Rise within 400 y of warming (equivalent to 0.8 °C of warming as a result of the linear temperature increase over 2,000 to 3,000 model years) (Figs. 5 and 6) and ultimately restricted ice across the wider WSE (*SI Appendix*, Fig. S20).

We caution that, for the LIG, subsurface ocean warming is poorly constrained. While recent work has suggested that sea surface warming may propagate to depths important for ice shelves (including embayments) within a few decades (103, 104), proxy SSTs could instead record “bottom-up” warming (i.e., as a consequence of circulatory change) and may underestimate the magnitude of the warming. We however, consider, that warming of +2 °C is likely to be at the upper end of potential LIG warming scenarios (14) and the forcing used here in our simulations to be conservative. Recent work using PISM showed that substantial collapse of WAIS is possible within only a few centuries even under modest warming (105). Those simulations used a much stiffer bed parameterization and were run at 5-km resolution. Other studies have suggested with 5-km resolved PISM simulations that, if mass loss comparable to recent decades is maintained for as little as 60 y, the WAIS could be irrevocably destabilized over subsequent millennia through the collapse in the Amundsen Sea sector (60), overcoming any isostatically driven rebound. On the basis of these comparisons, we can be confident that our inference of substantial mass loss from WAIS under modest ocean/atmosphere warming is not especially dependent on the model used, the way that the bed is parameterized, or the resolution of the simulations. Modeled Antarctic ice sheet contributions to global sea level are provided in Table 1. The ice sheet model data are available upon request.

## Supplementary Material

Supplementary File

Supplementary File
