# Supplementary material for: Early Last Interglacial ocean warming drove substantial ice mass loss from Antarctica
Source: Proc Natl Acad Sci U S A. 2020 Feb 11;117(8):3996–4006. doi: 10.1073/pnas.1902469117 (PMC7049167; doi:10.1073/pnas.1902469117)
Supplement: Supplementary File [file pnas.1902469117.sapp.pdf]

Supplementary Information Appendix for 'Early Last Interglacial ocean warming drove substantial ice mass loss from Antarctica' by Turney *et al.*

SI METHODS

<u>Patriot Hills</u>

Site Description

The Patriot Hills BIA (Horseshoe Valley, Ellsworth Mountains; 80°18′S, 81°21′W) is a slow flowing (<12 m yr^{-1}) compound glacier system situated within an over-deepened catchment that coalesces with the Institute Ice Stream at the periphery of the WSE (Figs S1-3) (1-5). Airborne radio-echo sounding (RES) surveys across the Ellsworth Mountains have revealed several wide (up to 34 km across) and long (260 km) subglacial troughs containing ice up to 2620 m thick (Fig. 1) (1). Airborne radio-echo sounding (RES) surveys across the Ellsworth Mountains have revealed several wide (up to 34 km across) and long (260 km) subglacial troughs containing ice up to 2620 m thick (Fig. 1) (1), along the side of which, two radar zones have been interpreted to indicate layers of ice with contrasting physical properties, consistent with snow deposited during previous glacial/interglacial transitions. In contrast to the other troughs across the Ellsworth Mountains, contemporary ice within the Horseshoe Valley Trough maintains the slowest average flow speeds of all, at 12 m a^{-1} (cf. the main trunk of the Institute Ice Stream reaches speeds up to 415 m a^{-1}). This is in large part due to the configuration of the Horseshoe Valley Trough where the ice thickness measures in excess of 2000 m at the head of the valley and reduces to approximately 1400 m downstream; towards the mouth of the valley, a subglacial ridge is found at approximately 200 m below sea level with the ice thickness some 750 m thick (Fig. S3) (6). The new Digital Elevation Model (DEM) data for the WSE is available at

https://data.bas.ac.uk/full-record.php?id=GB/NERC/BAS/PDC/00937. The configuration of the bed and resulting slow flow in Horseshoe Valley has two major benefits for our study. It allows: 1. a long record of ice to accumulate; and 2. the isolation and preservation of ice during periods of regional and Antarctic-wide mass loss.

In the lee of a small mountain chain at the end of Horseshoe Valley called Patriot Hills, strong local katabatic winds descend into the valley from the polar plateau, ablating the ice sheet surface by up to 170 kg m^{-2} yr^{-1} (5). As a result, ancient ice is drawn up from depth in the Horseshoe Valley Trough to form an extensive BIA (more than 1150m across; Fig. S4) (3, 7, 8). Whilst previous studies using cosmogenic nuclide data in the region cannot rule out the loss of the WAIS during relatively short interglacial periods (9), the airborne radar surveys across the Ellsworth Mountains have identified the presence of at least two ice units distinguishable from the bed and the upper ice between the major trough systems (1) (Fig. 1 and Fig. S2), implying punctuated periods of accumulation (and loss) during the late Pleistocene. High-resolution analysis of the BIA using ground-penetrating radar (GPR) and isotopes demonstrates a remarkably consistent pattern of layering and coherent trends out from Patriot Hills with three distinct unconformities (given as the distance on the surface along the transect relative to an arbitrary datum): at 247 m (D1), 360 m (D2) and -339 m (D0) (Fig. 1, and Figs S6 and S7). These unconformities are interpreted as either periods of BIA formation within Horseshoe Valley, or breaks in the record (3). Based on the abrupt shift in isotopic values over -335 and -339 m we suggest that unconformity D0 reflects the surface expression of the uppermost basal ice identified by airborne radar (1) preserved as a result of a glacial/interglacial paleoclimate switch recently recognised for the WSE (2, 10), a situation similar to that recorded in Greenland through ice fabric analysis in the NEEM ice core (11). Based on the trace gas, tephra and isotopic values of the surface ice beyond D0 (closest to

Patriot Hills) we interpret this section of the record to be Termination II in age (see below). No glaciomarine sediments have been identified at any of the boundaries.

Chronology

Chronological control across the profile is provided by a comprehensive suite of trace gas samples and volcanic tephra horizons along the BIA transect. The trace gas measurements provide a range of possible age solutions, which together with the absolute constraint provided by the tephra horizons, allows the development of a robust chronological framework that can be tied directly to the isotopic profile through high-resolution GPR survey (3). The available constraints suggest the 1156-m long Patriot Hills BIA transect spans time intervals from ~134.2 to ~1.3 kyr comprising four key zones: 4 (-362 to -339 m, equivalent to 134.2±2.2 to 130.1±1.8 kyr), 3 (-326 to 240 m, equivalent to 80±6.1 to 22.7±2.8 kyr), 2 (240 to 360 m, equivalent to 22.7±2.8 to 10.3±0.4 kyr) and 1 (360 to 800 m, 10.3±0.4 to 1.3±0.6 kyr).

A. Trace gas analyses. A comprehensive suite of trace gas samples – carbon dioxide (CO_2), methane (CH_4) and nitrous oxides (N_2O) – were taken along the Patriot Hills BIA transect. A Kovacs 9 cm diameter ice corer was used to collect ice at key sampling locations along the BIA transect with gas samples taken from >3 m depth to minimise the risk of modern air contamination and/or alteration identified in surface samples during a previous study (8) (https://www.youtube.com/watch?v=zcGArAlQ0u8&t=10s) (Fig. S8). The samples were double bagged and sealed in the field and transported frozen to CSIRO's ICELAB facility in Melbourne for the extraction and measurement of trace gases using a modified dry extraction 'cheese grater' and cryogenic trapping technique (12, 13). The trapped air samples were analysed by gas chromatography (GC) and the trace gas concentrations are reported against the calibration scales maintained by CSIRO GASLAB (14). Where sufficient material was

available, duplicates were analysed for reproducibility. To assess possible modern atmospheric contamination, we undertook analysis for the synthetic gas sulphur hexafluoride (SF_6) on a sample sub-set. The average concentration of eight samples analysed for SF_6 was approximately 5% of modern day atmospheric concentrations, and less than 2% for the two samples selected to develop the chronology (with values ranging from 0.01-0.14 ppt; molar parts per trillion in dry air).

The gas concentrations of different trace gases from the same samples provides a possible chronological framework for the Patriot Hills BIA. To explore the possible age solutions, we first compared the CH_4, CO_2 and N_2O measurements for each sample to determine whether they have a common age solution against the recently produced 156 kyr smoothed global time series for these gas species (15). The approach was similar to that reported previously (7). We attributed the gas samples to a likely age range based on the values and trends of the CH_4 measurements: Holocene, Termination, Glacial and Last Interglacial. We then produced a Gaussian distribution for each sample starting with the sample on the transect closest to the Patriot Hills and projecting the probability distribution onto the recently reported 156 kyr spline of trace gas measurements (15). The samples were ordered so that only the interceptions younger then the ages of the previous (older) sample were retained; note, we also reversed the procedure, starting along the transect furthest from the Patriot Hills, and found no appreciable difference in the results. An age distribution was calculated for CH_4 (1σ). The same approach was taken for CO_2. A combined distribution for CH_4 and CO_2 was then calculated based on a 'z'-like test:

$$z = (CH_4 - CO_2)/\sqrt{(\sigma_{CH4}^2 + \sigma_{CO2}^2)}$$

where CH_4 is the calculated mean methane age in years, CO_2 is the calculated mean carbon dioxide age in years, σ_{CH4} is the 1σ (68% confidence limit) of the methane age range, and σ_{CO2} is the 1σ (68% confidence limit) of the carbon dioxide age range. If the results did not

agree to within <0.5, the 'CH_4 only' measurement was retained. The above procedure was then repeated with N_2O, with the calculated age compared to the 'CH_4/CO_2' age distribution or those of the 'CH_4 only' age range. A combined distribution was calculated if they agreed using the above criteria. If the results did not agree, the 'CH_4 only' measurement was retained for final age calculation (Table S3). The guiding principle behind the dating procedure is that CH_4 is the most reliable gas, as it is hard to produce *in situ* and it is generally not altered by post-coring melting as much as CO_2 and N_2O. Importantly, the two samples closest to the Patriot Hills record relatively high CH_4 concentrations: 648±10 ppb at -362 m and 612±41 ppb at -346 m, implying the presence of LIG ice. However, as described in detail below, the detection of a tephra geochemically correlated (major oxides and trace elements) to tephra layers found in marine sediments from the West Antarctic continental margin and the Dome Fuji ice core, provides a chronological tie-point that identifies this ice as Termination II in age. Although the anomalously high CH_4 concentrations preserved in Patriot Hills could be interpreted as evidence for sustained discrete flux of methane via active venting (16), the relatively high accompanying CO_2 values (Table S3) may instead indicate that there has been some *in situ* production or secondary contamination, possibly a consequence of relatively warm conditions, as observed in the LIG section of the NEEM ice core record (17). Regardless of the cause, we excluded the anomalously high old gas values from our age model.

B. Tephra. The presence of visible tephras (volcanic ash marker horizons) provides additional chronological control for the Patriot Hills BIA. Previous work has identified high concentrations of colourless and light brown glass particles in dark bands outcropping at the surface along the profile at 282, 279 and 190 m (distances relative to datum) (7), correlated to the tephra horizon TD822a in the East Antarctic Talos Dome ice core (18), the Marie Byrd Land tephra WCM93-25 (19), and the SDMA-5951c tephra set in the West Antarctic Siple

Dome ice core (20), respectively. Here we report two new dark bands on the surface of the Patriot Hills at 10 m and -340 m, both observed at the surface as ~4 cm thick units of dispersed shards that can be traced parallel to the Patriot Hills (Fig. S9). The 10 m sample contained a high concentration of light brown volcanic glass shards with a blocky morphology; the shard size (longest axis) ranged between 12.5 and 70 µm with a mean value of 33 µm. A minor population of colourless glass shards was also found in the same sample. The ratio between the light brown and colourless glass shards is 16:1. The -340 m layer was found to contain a high concentration of colourless and fluted volcanic glass particles; particle size ranged between 20 and 80 µm with a mean of 34 µm. Shards were extracted by centrifugation of the melted ice samples and put onto a glass slide for electron microprobe analysis. The slides were ground and polished using silica carbide paper and decreasing grades of diamond suspension to expose fresh sections of glass. Single-grain analyses of ten oxides were performed on a Cameca SX-100 electron microprobe at the Tephrochronology Analytical Unit, University of Edinburgh. The instrument is equipped with 5 wavelength dispersive spectrometers and the operating conditions followed that of ref. (21): accelerating voltage of 15kv, a beam current of 2nA for Na_2O, K_2O, SiO_2, Al_2O_3, MgO, FeO (total) and CaO and 80 nA beam current for P_2O5, TiO_2 and MnO. The beam diameter was 5 µm. Secondary standards (Lipari and BCR2G) were analysed at regular intervals. Geochemical results are provided in Table S1.

The shards from 10 m reveal a dominant basanitic population (brown shards) with a minor trachytic population (colourless shards) (Fig. S10 and Table S1). We focus on the basanitic component for establishing a correlation given the dominance of the brown shards in this sample. The shards from -340 m are trachytic in composition and exhibit a tightly-clustered population (Fig. S11). The populations were compared to tephras of all ages reported from EPICA Dome C (EDC) (22-24), Dome Fuji(25), Mount Moulton (26), Siple

Dome (20), Talos Dome (27-29), Vostok (23, 30), the West Antarctic continental margin (31), and the WAIS Divide (32).

Similarity coefficient (SC) analysis (33) for the 10 m layer reveals the closest match to be the basanite Tephra C from the WAIS Divide at 3149.12 m (SC = 0.98), equivalent to 44.9±0.3 kyr (32). The -340 m layer revealed that the closest match to be Dome Fuji 1785.14 m (SC = 0.966; data previously unpublished) (Table S1 and Fig. S11), with lower correlations for Dome Fuji 1361.89 m (SC = 0.932) (25), EDC 1265.1 m (SC = 0.957) (22, 24) and Vostok 1995 m (SC = 0.942) (30). Whilst the plots reveal partial compositional similarities between the different populations, the highest SC and relatively high MgO and TiO_2 values strongly suggests the Patriot Hills tephra at -340 m is the same horizon as that identified in Dome Fuji at 1785.14 m. This tephra is dated to 132.7±0.8 kyr according to the Dome Fuji timescale (34) and 130.7±1.8 kyr on the AICC2012 timescale (35, 36). Importantly, a widespread tephra found in marine sedimentary records on the West Antarctic continental margin (Tephra B) has been proposed to correlate to Dome Fuji 1785.14 m but the correlation has until now remained only tentative in the absence of any reported geochemistry from the latter (31). A further two tephras (Tephra A and C) are also found in some marine cores. Tephra A (~80 kyr) can be discriminated by geochemistry from the Patriot Hills tephra but Tephra C (~132 kyr BP) is indistinguishable from Tephra B (Fig. S11); Tephra C, however, is only identified in a few cores and is found stratigraphically below Tephra B. A range of stratigraphical constraints indicate Tephra B equates to Termination II or early Marine Isotope Stage 5e, coincident with the onset of high biogenic opal deposition representing the maximum in biological productivity during peak warm times on both the West and East Antarctic margin and in the Southern Ocean (Fig. 2) (37, 38). A subsequent decrease in productivity results in a deepening of the Calcite Compensation Depth (CCD) and preservation of calcareous foraminifera in continental margin sediments,

reflected by high $CaCO_3$ contents (31, 39) (Fig. S12). Here we find the major oxides from Tephra B have a close match to the Patriot Hills tephra horizon at -340 m (SC = 0.948), consistent with this interpretation.

To confirm the above correlation, we undertook trace element analysis of the glass shards from Patriot Hills at -340 m. Unfortunately, the shards from Dome Fuji were too thin for analysis. However, we were able to undertake trace element analyses on Tephra B samples from two marine sediment cores from the western Antarctic Peninsula continental margin: PC108 (4.65 m depth) and PC111 (6.86 m depth) (31). Trace element analysis of volcanic glass shards were performed using an Agilent 8900 triple quadrupole ICP-MS (ICP-QQQ) coupled to a Resonetics 193nm ArF excimer laser-ablation in the Department of Earth Sciences, Royal Holloway, University of London (40). Full analytical procedures for volcanic glass analysis are reported in ref. (41). Spot sizes 20 and 25 µm were used depending on the vesicularity and/or size of glass surfaces. The repetition rate was 5 Hz, with a count time of 40 s on the sample, and 40 s on the gas blank to allow the subtraction of the background signal. Blocks of eight or nine glass shards and one MPI-DING reference glass were bracketed by the NIST612 glass calibration standard (GeoREM 11/2006). In addition, MPI-DING reference glasses were used to monitor analytical accuracy (42). The internal standard applied was ^{29}Si (determined by EPMA analysis). LA-ICP-MS data reduction was performed in Microsoft Excel (41). Accuracies of LA-ICP-MS analyses of ATHO-G and StHs6/80-G MPI-DING glass were typically ≤ 5%. Identical trace element glass chemistries (Fig. 3 and Table S2) strongly support the correlation of the Patriot Hills -340 m tephra horizon and the West Antarctic marine Tephra B(31) which is in turn correlated to the Dome Fuji tephra 1785.14 m (25, 43), the source of which was probably an eruption in the Marie Byrd Land volcanic province (West Antarctica) (31). The recognition of a widespread tephra

horizon across a large sector of the Antarctic at the very onset of the LIG provides a time-parallel marker horizon crucial for future studies investigating Antarctic ice-sheet mass loss.

C. Development of a Bayesian age model. To fully exploit the volcanic (tephra) horizons and trace gas samples we develop a Bayesian age model for the Patriot Hills BIA. The trace gas measurements provide a range of possible age solutions, which together with the absolute constraints provided by the tephra horizons, allows the development of a robust chronological framework that can be tied directly to the isotopic profile through high-resolution GPR survey (3, 8). We undertook Bayesian age modelling using a Poisson process deposition model (P_sequence) (44, 45) in the software package OxCal v.4.2.4 (https://c14.arch.ox.ac.uk/oxcal.html) to integrate the above accepted gas measurements with the tephra ages and develop an age model with uncertainty estimates (44, 46) (Tables S3 and S4). Using Bayes theorem, the algorithms employed sample possible solutions with a probability that is the product of the prior and likelihood probabilities. Crucially, Bayesian modelling enables the relative stratigraphic information from the BIA transect to be incorporated along with the ‘calibrated likelihoods’ or ‘calibrated probability distributions’. Bayes’ theorem is given as:

$$p(\theta|y) \propto p(y|\theta)\, p(\theta)$$

Here the term $p(y|\theta)$ describes the ‘likelihood’, where p is a probability function (the symbol | representing ‘given’). The ‘calibrated’ probability distribution therefore represents the likelihood. The other term $p(\theta)$ defines the ‘prior’ which describes the possible values of unknown parameters before the observation of the data we collect. Thus, the ‘posterior’ – shown here as $p(\theta|y)$ – is a probability function that quantifies the level of confidence associated with the unknown parameters after the observation of the data (47, 48). The posterior distributions thus allow us to determine probability distribution functions for the periods of ice accumulation.

'Calibration curves' with 20 year resolution were developed for the three trace gas species using the 156 kyr time series (15). Each gas species was given a Delta_R term by determining the standard deviation of the trace gas datasets reported by Kohler *et al.* over the full time range to allow for uncertainty between the measurements at Patriot Hills and those reported in these calibration curves. The Delta_R term was calculated using the 'f' and 't' terms. The 'f' variable sets the default error on the measurements as a factor of the standard deviation in reported calibration curves; the 't' value sets how tight the tails are on the Delta_R (i.e. how close or far the mean is to a Normal distribution). Our age model used a 'f' value of 0.3 and a 't' value of 2 degrees of freedom. Taking into account the deposition model, the actual age measurements provided by the tephras, and the common age solutions offered by the trace gas measurements, the posterior probability densities quantify the most probable age distributions.

We used boundaries for the hiatuses (D0-D2), effectively allowing OxCal to model the ages within each section independent to one another. If associated chronological/climate information or tephra was able to provide a first-order age estimate of the event, we used a *Top Hat* boundary. For instance, based on the isotopic profile, D2 at 360 m suggests the transition to the Holocene (49) and we therefore used a *Top Hat* boundary of 11.65±2 kyr. For the oldest part of the sequence beyond -339 m (i.e. that part of the BIA transect closest to the Patriot Hills), the presence of a Termination II tephra indicates the relatively high trace gas measurements in the oldest part of the transect are anomalous and there is no gas age solution using the criteria above. We determined a first-order chronology back to -362 m by using a *Top Hat* boundary of 133±4 ka at D0 informed by the age of the Dome Fuji 1785.14 m tephra at 130.7±1.8 kyr and the slowest rate of 'accumulation' through the rest of the dated sequence. To account for the gas-ice age difference (Δage), we used a Delta_R term for the different sections of the BIA, guided by recently reported values from the WAIS Divide ice

core (50), the closest site to Patriot Hills with reported values. For the glacial-age ice we used the prior U (-500,-200), and for the Holocene U (-300,-150). For the glacial-age ice, we found a mean Δage of 350±87 years; for the Holocene we determined a Δage of 225±43 years. It is important to note that if the Δage is outside the range reported from the WAIS Divide ice core (50) it would not substantially alter our chronology (i.e. the change in our timescale would be on the order of centuries). In OxCal, commands or parameters are written in a C++ CQL (Command Query Language). The code used to calibrate the trace gas measurements and tephra ages in OxCal is provided in Table S4. The calculated Agreement Index is extent to which the final (posterior) distribution overlaps with the original distribution; an unaltered distribution will have an index of 100% but it is possible for the value to rise above this if the final distribution only overlaps with the very highest part of the prior distribution. For the Patriot Hill age model we calculated an Agreement Index of 101.6% ($A_{overall}$=71.2%) i.e. exceeding the recommended rejection Agreement Index threshold of 60% (ref. (46)). The age model failed to run with the inclusion of the two oldest gas samples. With no tephras so far identified in the Holocene part of the BIA, we consider the chronology for this part of the sequence to be preliminary only.

Isotopes

δD and $\delta^{18}O$ isotopic measurements were performed at either 1 or 3 m resolution along the Patriot Hills BIA transect at James Cook University (JCU) using Diffusion Sampling - Cavity Ring-down Spectrometry (DS-CRDS) (International Atomic Energy WICO Lab ID. 16139) (51) (Fig. 2, Figs. S7 and S13). This system continuously converts liquid water into water vapour for real-time stable isotope analysis by laser spectroscopy (Picarro L2120-i, Sunnyvale, CA, USA). Ice samples were thawed overnight and 5 mL transferred to auto-sampler vials covered with foil caps. An automated sampling system was used to pump each

sample to the diffusion cell for isotopic measurement for 15 min. Each analytical run consisted of 12 standards interspersed with 44 unknown samples. Data processing was performed using a customised Excel™ template and included correction for between-sample memory, instrumental drift and normalization to the VSMOW scale. The correction for memory effect was determined in a series of experiment with standard waters of known composition. It was found that 94±1% of the changes in $\delta^{18}O$ and δD values from the previous sample were recorded in the first replicate of a subsequent sample. This percentage remained constant irrespective of the size or direction of change. Due to the volume requirement of the DS-CRDS instrument and small sample size (5 to10 mL) only one measurement could be made of most ice samples. Six vials of each of two working standards were analysed in each run: Casey Snow Melt ($\delta^{18}O$ = -18.02‰; $\delta^{2}H$ = -140.4‰) and Blue Ice ($\delta^{18}O$ = -39.14‰; $\delta^{2}H$ = -311.3‰). The isotopic compositions of the working standards on the VSMOW scale were determined relative to IAEA standards VSMOW2, VSLAP2 and GISP by laser spectroscopy (Picarro L2120-i CRDS with injection/vapourization attachment) and by IRMS (IAEA Stable Isotope Laboratory, Vienna). Replicate standard analyses yielded the following long-term precision (1σ, 10 runs): Casey Snow Melt: = 0.20‰, δD = 1.28 ‰, δ*d* (excess) = 0.84 ‰ (n=43) and Blue Ice: $\delta^{18}O$ = 0.16 ‰, $\delta^{2}H$ = 1.06‰, δ*d* (excess) = 0.96‰ (n=46). Finally, to ensure reproducibility a subset of samples were rerun at UNSW ICELAB for δD and $\delta^{18}O$ using a Las Gatos Research Liquid Water Isotope Analyser 24d (International Atomic Energy WICO Lab ID. 16117). Reported overall analytical precision on long term ice core standards are <0.32‰ for δD and <0.13 for $\delta^{18}O$ values. All isotopic values are expressed relative to the Vienna Standard Mean Ocean Water 2 (VSMOW2).

Ancient DNA analysis

Previous reports have confirmed the presence of prokaryotes in West Antarctic ice and utilised the cellular abundance of these species to infer biological signals of offshore environmental change (52, 53). Here, we take a step further and develop a novel strategy to obtain ancient DNA from large discrete ice core samples. To obtain the samples, a Kovac corer was thoroughly cleaned with 1-3% bleach and wiped with 95% ethanol between core extractions to minimise cross contamination. After coring, the top 1 m of ice was removed and discarded, and the remaining 1-2 m of the cores were collected in 50 cm sections and immediately placed into new PFTE flexible plastic tubing. The tubing at the top and bottom of the core was sealed with a heat sealer, and the process was repeated to encase the core in a second layer of the plastic tubing for protection during transport. Within 6 hours of extraction, the tubing-encased BIA cores were transported to a dome tent and hung inside to melt via solar radiation over 24 hours, using black plastic bin liners around the plastic tubing to speed up the process. Once the BIA sample was melted, the liquid was directly transferred from the inside layer of tubing into a hand-powered vacuum filtration system cleaned with 1-3% bleach and ethanol wipe between samples. Water from each sample was filtered through disposable, sterile 0.45 μm nitrocellulose filters to collect whole bacterial organisms trapped in the ice during its formation, and reduce noise caused by environmental DNA. Filters were stored in sterile plastic bags, frozen at -20°C, and returned to the Australian Centre for Ancient DNA (ACAD) in Adelaide, Australia, for ultra-clean genetic analysis within a specialised ancient DNA facility. The novel ability to process very large volume samples of continental Antarctic ice in the field (i.e. ~7 kg per temporal sample) creates a powerful new opportunity to generate sufficient concentration to permit detailed genetic biodiversity surveys.

Strict ancient DNA methodologies designed to assess low-biomass microbial samples were applied at all times (54). Within the ancient DNA laboratory, all work was conducted within a bleach and UV-treated hood in a still air room. Each 0.45 µm nitrocellulose filter was cut in half using a sterile scalpel blade. Using sterilised tweezers, one half of the filter was extracted using the PowerLyzer Soil DNA Isolation kit (MOBIO, Carlsbad, CA, USA), following the manufacturer's instructions, and the second half was extracted using a method with cetyl trimethylammonium bromide (CTAB) (55). Both extraction methods were employed to examine microorganisms with different cellular wall structures. Extraction blank controls (EBCs; an extraction that contains no sample), control samples from the field (i.e. swabs of cleaned coring and filtering equipment prior to use), and blank filters were processed in parallel to monitor background DNA levels from the field and laboratory. All DNA extracts and controls were amplified using published, universal bacterial 16S ribosomal RNA (rRNA) primers that are modified to include Illumina sequencing adapters and a unique sample specific 12 bp Golay barcode (56): forward primer 515F (AATGATACGGCGACCA CCGAGATCTACACTATGGTAATTGTGTGCCAGCM GCCGCGGTAA) and barcoded reverse primer 806R (CAAGCAGAAGACGGCATACGAGATnnnnnnnnnnnnAGTCAGTCAGCCGGA CTACHVGTWTCTAAT). Similar approaches have been previously undertaken to estimate bacterial content of ice and permafrost samples (57, 58). PCR amplifications of the 291-target region were performed in a 25 µL reaction mix containing: 2.5 mM $MgCl_2$, 0.24 mM dNTPs, 0.24 µm of each primer, Invitrogen Platinum HiFi Taq polymerase in 10x reaction buffer (Applied Biosystems, Melbourne Australia), and 2 µL DNA extract. The PCR protocol included the following parameters: 6 mins at 95°C, followed by 35 cycles of 95°C for 30 sec, 50°C for 30 sec, and 72°C for 30 sec, and a final extension at 60°C for 10 mins. PCR amplifications were performed in triplicate for each sample and control to minimise PCR

bias, and a no-template PCR amplification control was included to monitor background DNA levels in PCR reagents. Pooled PCR products were purified using an Agencourt AMPure XP PCR Purification kit (Beckman Coulter Genomics, NSW) and quantified using the HS dsDNA Qubit Assay on a Qubit 2.0 Fluorometer (Life Technologies, Carlsbad, CA, USA). Purified PCR products from all samples were then pooled at equimolar concentrations, and the final pool was diluted to 2nM for sequencing using a 300 cycle, 2x150bp Illumina MiSeq kit.

After DNA sequencing, all individually indexed 16S rRNA libraries were demultiplexed from raw bcl files using CASAVA (version 1.8.2; Illumina), allowing for one mismatch. Sequencing adapters were removed from reads using Cutadapt v.1.1, and sequences were quality filtered (i.e. reads >100 bp and >Q20 for 90% of each sequence) using fastx toolkit v.0.0.14 (https://github.com/agordon/fastx_toolkit.git). Resulting sequences were imported into QIIME v.1.8.0 (59) for all downstream analyses. Using UCLUST, sequences with greater than 97% similarity to the Geengenes v13 reference database (60) were binned into Operational Taxonomic Units (OTUs) by closed reference clustering. A representative sequence was selected by determining the most abundant sequence in each bin. An average of 98,411 sequences per sample were obtained (minimum of 18,730 and maximum 252,580 sequences per sample; Table S5). To attribute background bacterial DNA levels appropriately, a conservative approach was taken to filter background DNA signals from the blue ice samples. All OTUs detected in any control sample (e.g. laboratory and sampling controls) and human genera (identified by homology to species within the Human Oral Microbiome Database (HOMD) (61) were removed from the experimental samples before downstream analysis (62). Taxa within controls were dominated by genera known to contain contaminant taxa (an average of 75% of taxa within all controls; ranging 38% to 97%; Table S6) and included *Bradyrhizobium*, *Ralstonia*, *Comamonas*,

Acinetobacter, and *Pseudomonas* taxa (62) or human-associated taxa (*e.g. Streptococcus, Porphyromonas,* and *Fusobacterium*). After filtering, an average of 30.8% of the reads for each sample were retained (Table S5). Retained sequences from each extraction method were then pooled, and the resulting taxa present in each sample were explored as a proportion of the total filtered DNA sequencing reads.

Several of the taxa identified within the samples have been previously reported in frozen environments. For example, in the +250 m sample (equivalent to ~22 ka), the taxon *Hydrogenophilus* was detected which has also been found in accreted ice derived from Lake Vostok subglacial water in East Antarctica (57), while the taxon *Cryocola* has been reported from Arctic permafrost (58). To check for bias in the representation of taxa, the alpha diversity (representing within a sample diversity) was compared across the transect using Simpson's, Chao 1, and observed species indexes in QIIME with the following parameters (min: 20; max: 200; step: 10) (Fig. S14). Importantly, no statistically significant differences in alpha diversity were detected across the samples, suggesting that microbial deposition may have been similar through time. Therefore, this analysis suggests a marked ecological switch observed towards the end of Termination II in the Patriot Hills BIA (-340 m) which is characterised by the dominance of the microorganism *Methyloversatilis* (Fig. S15) which may reflect methane metabolism. While most methylotrophs are also methanotrophs, this is not always the case and further work is needed to explore the potential for methanotrophy in the *Methyloversatilis* genus. As an alternative scenario, the presence of methylotrophs may still be connected to methane release; for example, methylotrophs may be feeding from by-products of methane oxidation, such as methanol produced by microbes in the presence of methane (63). In this scenario, non-methanotrophic methylotrophs would be present in higher numbers alongside or after the release of methane, congruent with our data. Existing literature supports this hypothesis. Non-methanotrophic methylotrophs that use methanol and

other partially oxidized methane metabolites are found commonly alongside methanotrophs and support actively support methane oxidation (64). In fact, other non-methanotrophic methylotrophs (reliant on methanol and not methane) have been found at higher abundance alongside methanotrophs near a marine hydrocarbon seep (65). This would mean that the methanotrophs responsible for methane oxidation went undescribed in our amplicon analysis. This is not entirely unexpected; while our amplicon can detect some archaea, it certainly cannot detect them all, potentially leaving many potential methanotrophs in this dataset. We therefore consider our results to be consistent with the rapid destabilization of hydrate reserves in Antarctic sedimentary basins (16, 66). While the current sample numbers limit resolution, our study highlights the untapped potential of BIA genetic data to exploit cryosphere microbial communities to investigate glaciological and environmental change (53).

Ice sheet modelling

We employed the Parallel Ice Sheet model (PISM), version 0.6.3, an open source three-dimensional, thermomechanical coupled ice-sheet/ice-shelf model. PISM employs a stress balance that superposes solutions of the shallow-ice and shallow-shelf equations, and incorporates a pseudo-plastic basal substrate rheology to allow for realistic sliding over meltwater saturated sediments, a bed deformation model that simulates mantle dissipation and rebound arising from spatial changes in ice loading through time (67), and a sub-grid basal traction and driving stress interpolation scheme to allow realistic grounding-line motion (68, 69). A summary of key parameters and values are provided in Table S7. We prescribe a mantle viscosity of 1×10^{20} Pa s which is lower than the PISM default (1×10^{21}) and intended to capture more accurately the weaker mantle of West Antarctica, where the majority of mass loss takes place. The amount of water that saturates basal till varies spatially according to the enthalpy field but is limited in our implementation so that no more than a 2 m

thickness of saturated substrate accumulates. Here we use a scheme that allows the first grounded cell upstream of the grounding line to saturate to this maximum allowable depth (2 m), which smooths the velocity transition across the grounded-floating junction. We do NOT, however, use the sub-grid grounding line interpolation that increases grounding line sensitivity to external forcing. In the experiments presented here we chose not to implement the sub-grid scale interpolated ice shelf basal melt component of this scheme (15, 91). Calving is parameterised using horizontal strain rates and a minimum thickness criterion (220 m) (70, 71).

Surface mass balance depends on monthly climatological data and a positive degree-day model that tracks snow thickness and allows for melting of snow and ice at 3 and 8 mm ˚C^{-1} day^{-1} respectively. We incorporate a white noise signal (normally-distributed, mean zero random temperature increment) into the calculation of daily temperature variations. The standard deviation of daily temperature variability is set at 2°C, somewhat lower than the commonly employed value of 5˚C, on the basis that the later has a tendency to overestimate melt (72, 73). Surface temperatures are adjusted for elevation according to an altitudinal lapse rate of -8˚K km^{-1}, and a refreezing coefficient of 0.6 is used to mimic meltwater capture within the snowpack. Precipitation varies with temperature according to a model-based relationship (74) that is consistent with empirical data (75).

Using ocean temperatures from a present-day simulation of global climate (76) we implement a sub-ice shelf melt calculation that follows the boundary layer thermodynamics approach of refs. (77, 78) (Fig. S16). This scheme calculates the freezing point in the boundary layer from pressure (i.e., depth) and salinity, and thus derives melt rates from energy and salt flux gradients.

Different approximations of the temperature at the ice shelf base are used depending on whether there is melt, freeze on, or neither. Although this 'three-equation' model represents a simplification of ice-shelf/ocean interactions, the approach has been used effectively in other Antarctic ice sheet simulations (74, 79, 80).

Our experimental methodology is identical to that described in detail elsewhere (79, 81), but in summary we first collate datasets representing the physiography and climatology of the modern Antarctic ice sheet (82-84) and follow a sequence of simulations intended to produce a thermally evolved and dynamically stable present-day ice sheet configuration. This sequence uses an initial smoothing run of 20 years in which flow by internal deformation but with no basal sliding relaxes the ice sheet surface. The geometry is then held fixed for 150 kyr whilst the three-dimensional thermal field evolves. Finally, a 25 kyr run using full model physics is undertaken, at the end of which the final configuration is compared to empirical constraints (ice thickness, surface velocity, grounding and calving line positions). Tuning parameters (stress balance, basal resistance prescription, calving) are iteratively modified and the 25 kyr run repeated until an optimum fit is achieved. We use the above process to establish a stable starting geometry for our perturbation experiments. These experiments run for 20,000 years so that the modelled ice sheet is at (or close to) equilibrium. Climate and ocean temperature perturbations are applied as spatially-uniform linear increments added to boundary distributions representing present-day conditions. Linear increases take place between 2000 and 3000 model years. The first 2000 years (no forcing) allow any transient behaviour associated with model initialisation to take place in the absence of environmental perturbations, whereas the subsequent 1000 years force the ice sheet to evolve slowly to changes in air and ocean temperature and precipitation. The remaining 17 kyr allow a steady-state to be reached. All experiments were run at a spatial resolution of 20 km (Figs 5, 6, Fig. S19, and Table 1).

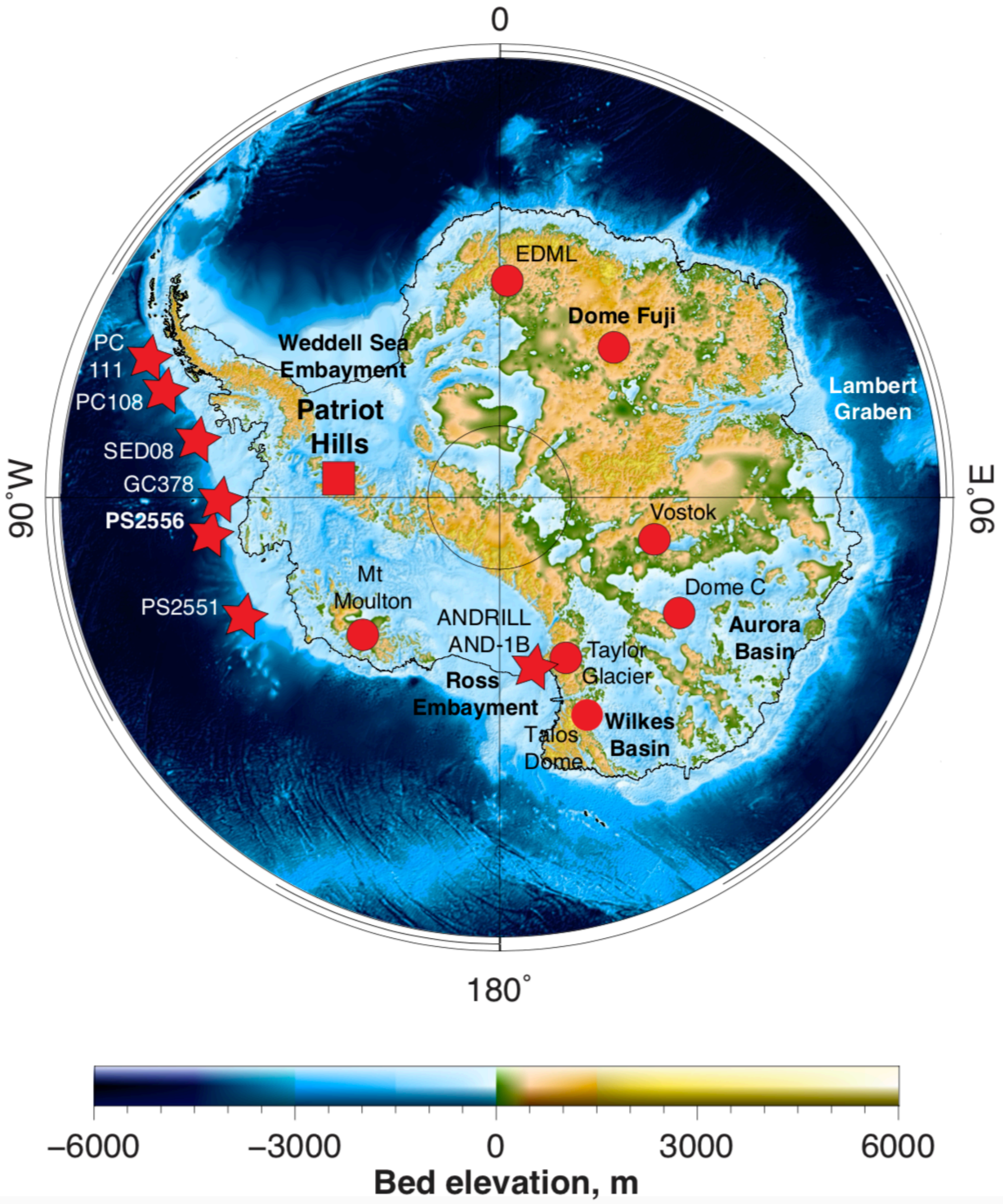

Fig. S1. Antarctic bathymetry with location of Patriot Hills, Ellsworth Mountains (square), ice cores with confirmed Last Interglacial (circles), key marine sediment cores (stars), and major basins described text. Figure generated using data from Bedmap 2 (82) and GMT (85).

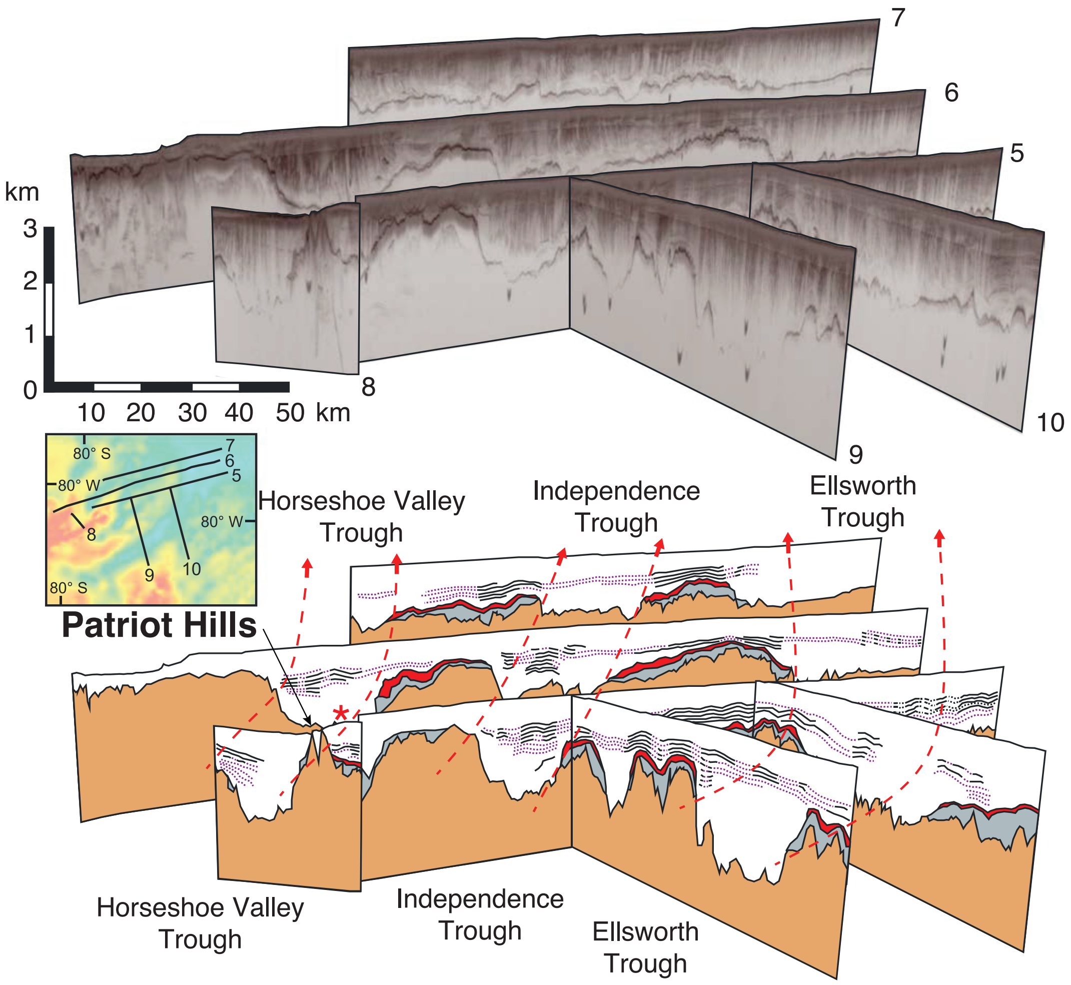

Fig. S2. Radar flight lines (inset) and three-dimensional schematic of the morphology of Horseshoe Valley, Independence and Ellsworth troughs in the upper Institute Ice Stream catchment (marked by the red dashed lines) and internal downstream stratigraphy (modified from ref. (1)); the location of the Patriot Hills BIA transect is shown by a red asterisk.

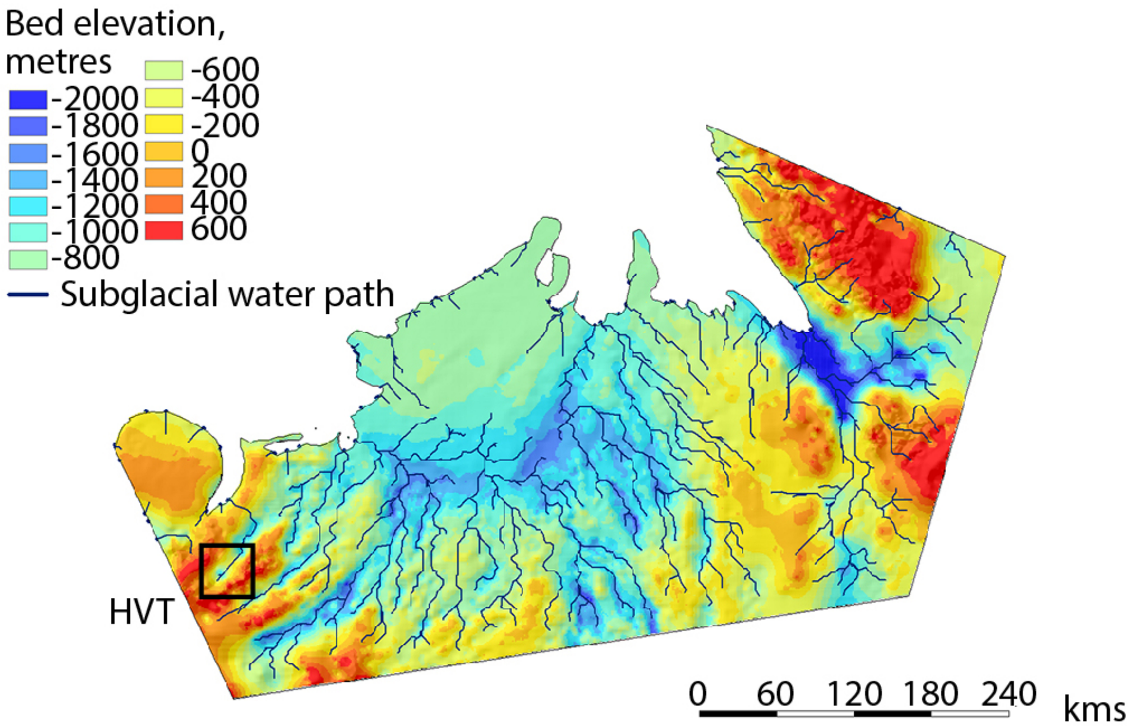

Fig. S3. Modified version of the new bed Digital Elevation Model for the Weddell Sea Embayment (6) highlighting the overdeepened trough and subglacial ridge in the Horseshoe Valley Trough (HVT).

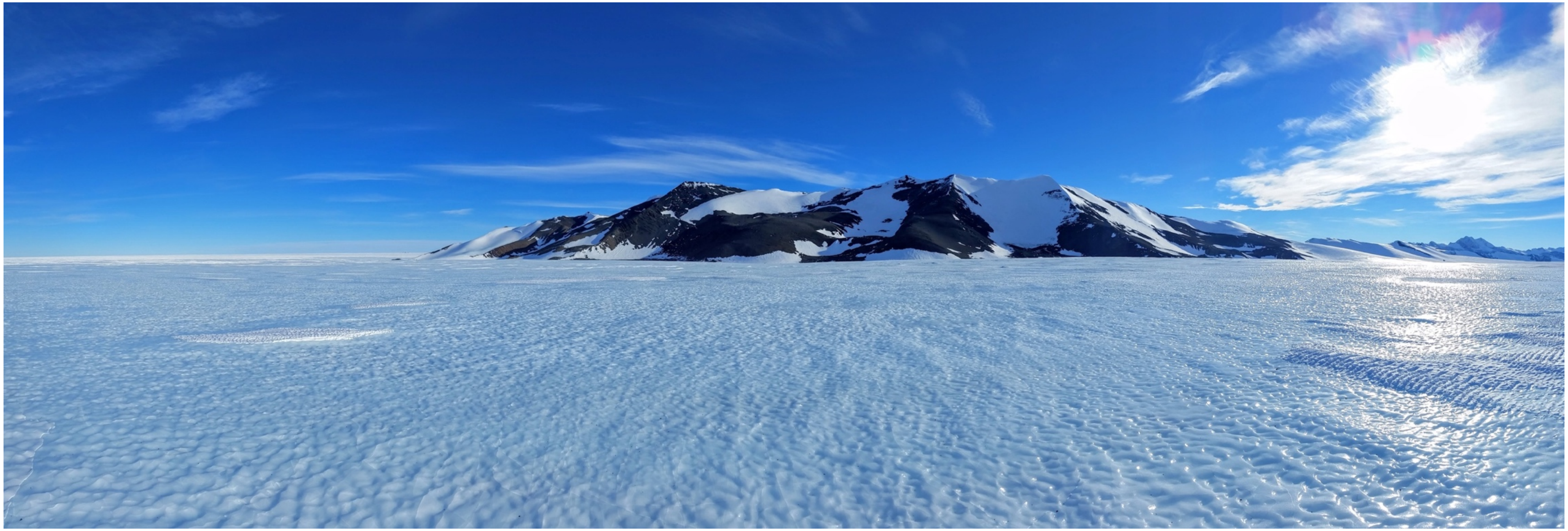

Fig. S4. The extensive Patriot Hills Blue Ice Area.

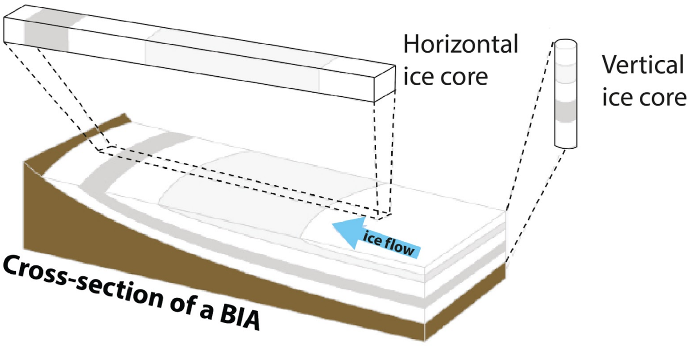

Fig. S5. Schematic cross-section of a Blue Ice Area (BIA) showing horizontal and vertical ice core records.

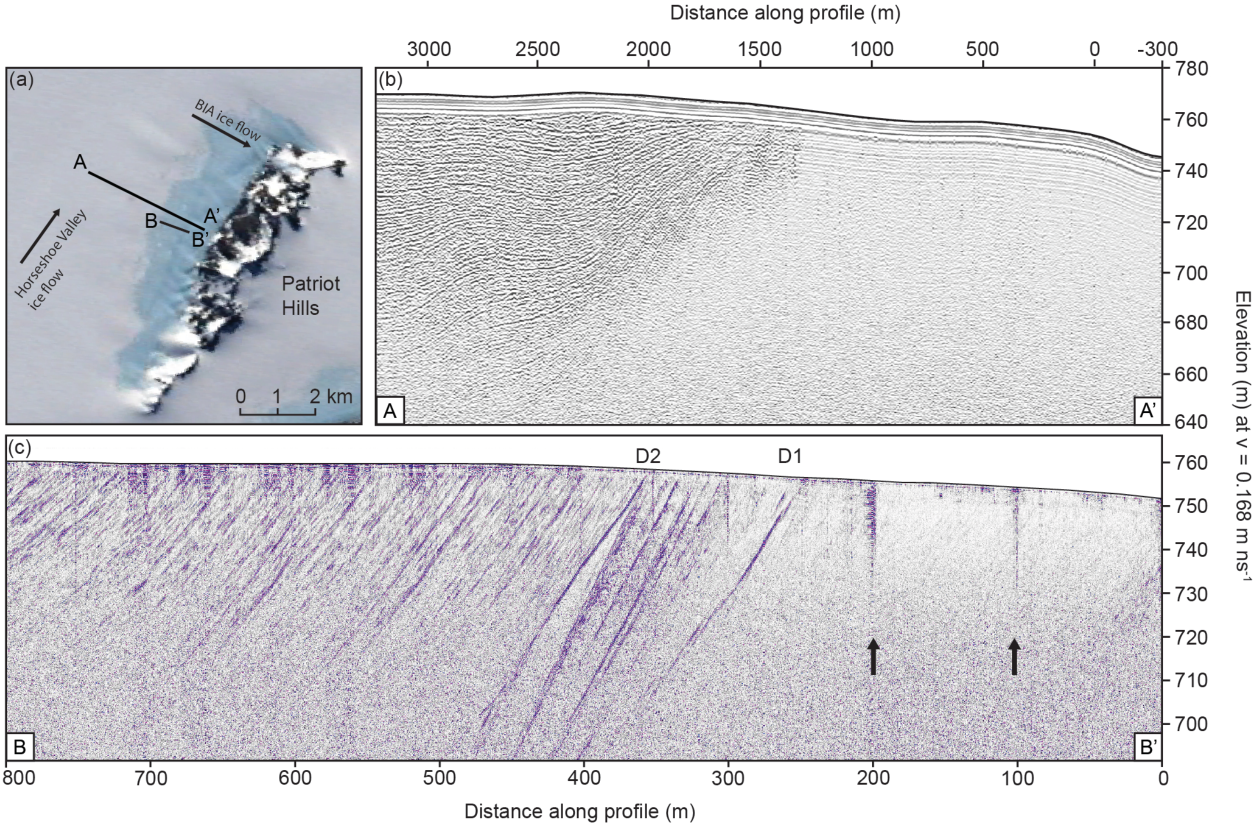

Fig. S6. Location of Patriot Hills (Horseshoe Valley, Ellsworth Mountains) and dominant ice flow locations (LIMA background image) (a). Snowmobile-towed ground penetrating radar (GPR) Transect A shows isochrone sequences in front of Patriot Hills (b), while step-and-collect mode GPR Transect B shows two discontinuities in an otherwise conformable stratigraphic record (arrows indicate vertical noise from boreholes) (c).

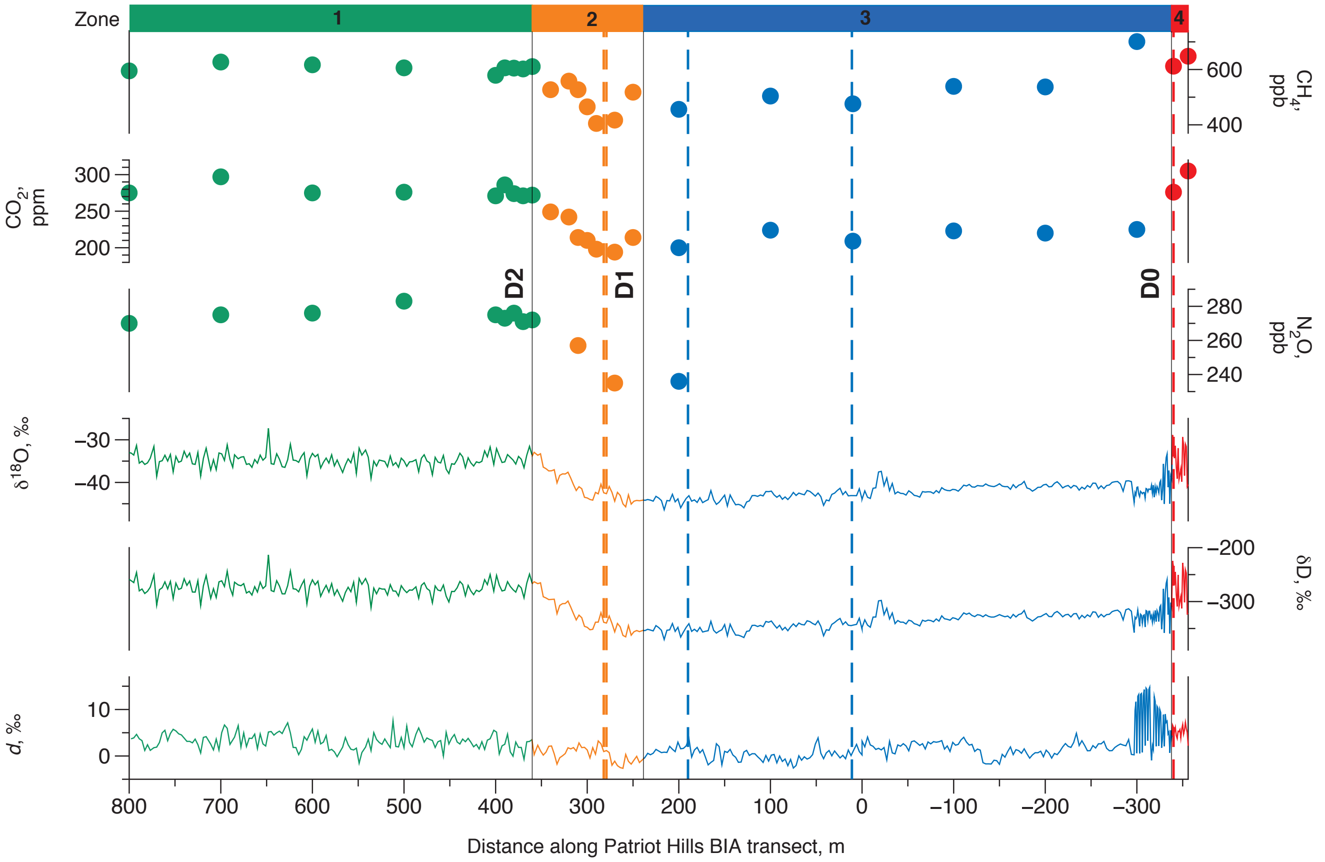

Fig. S7. Measured trace gases (CH_4, N_2O and CO_2) and stable isotopes ($\delta^{18}O$, δD and deuterium excess, *d*) plotted against distance along transect relative to an arbitrary datum. Ice transect zones 1-4 (green, 800 to 360 m; orange, 360 to 240 m; blue, 240 to -339m; red, -339 to -359 m) denote the sections representing the Holocene, Termination I, the last glacial period, and Termination II, respectively (note, these are not marine isotope stages). Solid black lines denote unconformities D0-2 identified by GPR and/or abrupt isotopic shifts (3). Dashed lines show the positions of geochemically characterised tephra horizons in the Patriot Hills sequence.

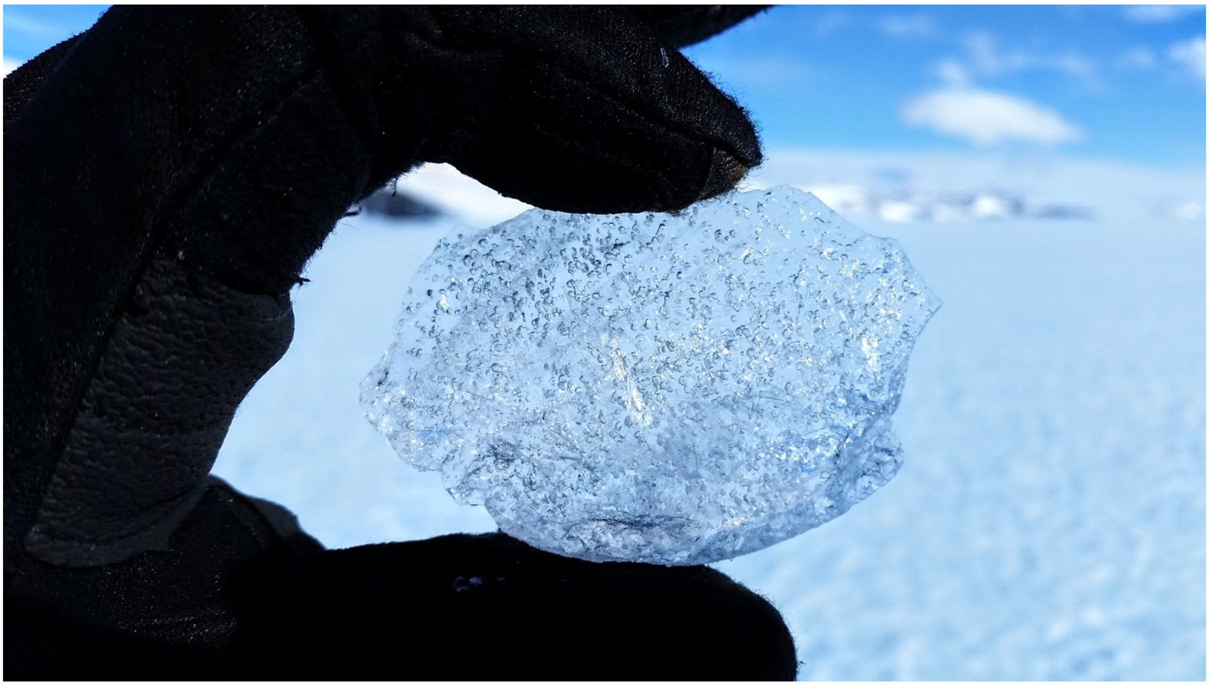

Fig. S8. Gas bubbles preserved in surface ice from -340 m along the Patriot Hills transect (Termination II).

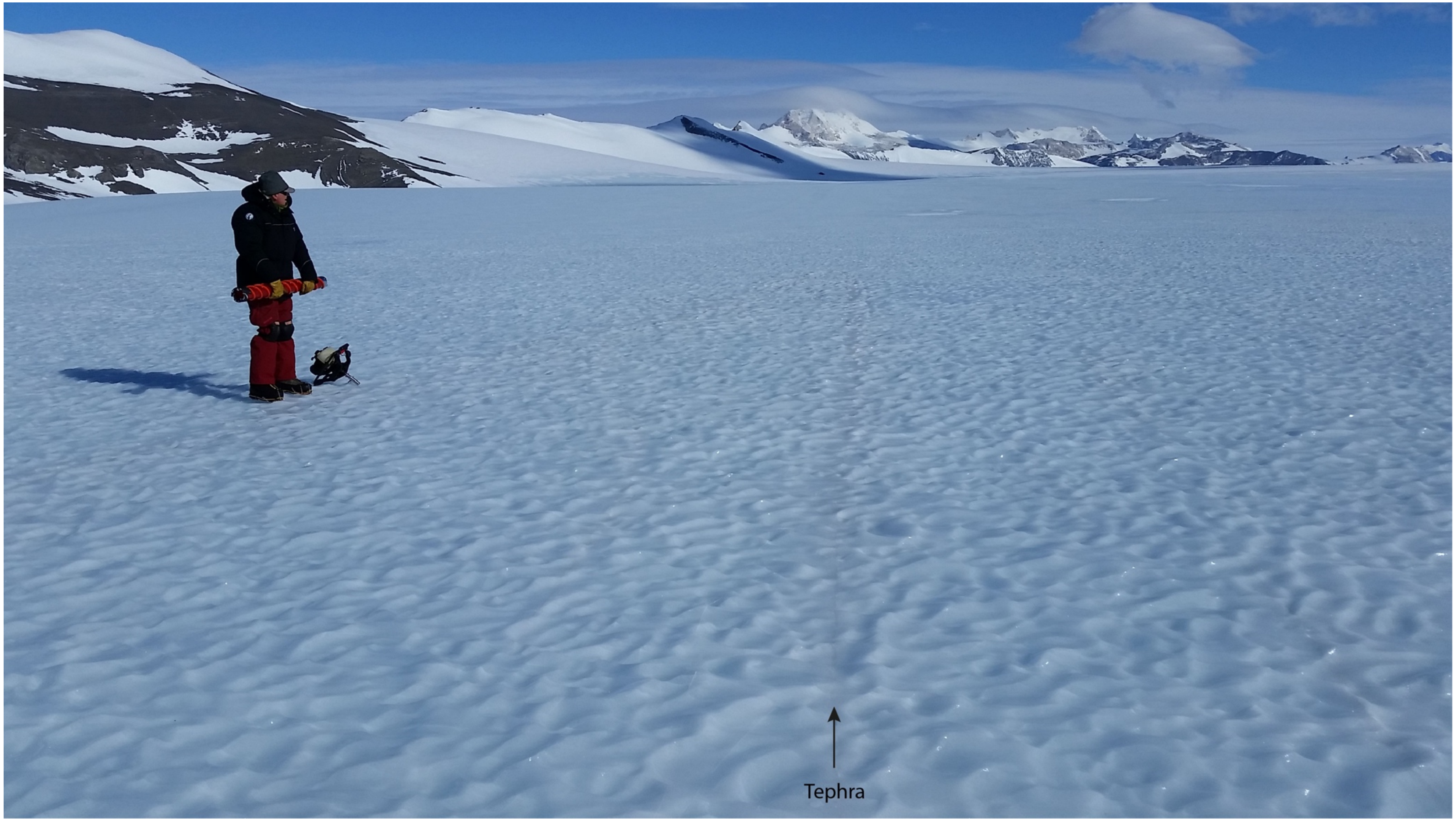

Fig. S9. Surface expression of the -340 m tephra at Patriot Hills (mid-picture). Note, the tephra can be followed parallel to the Patriot Hills for>400 m.

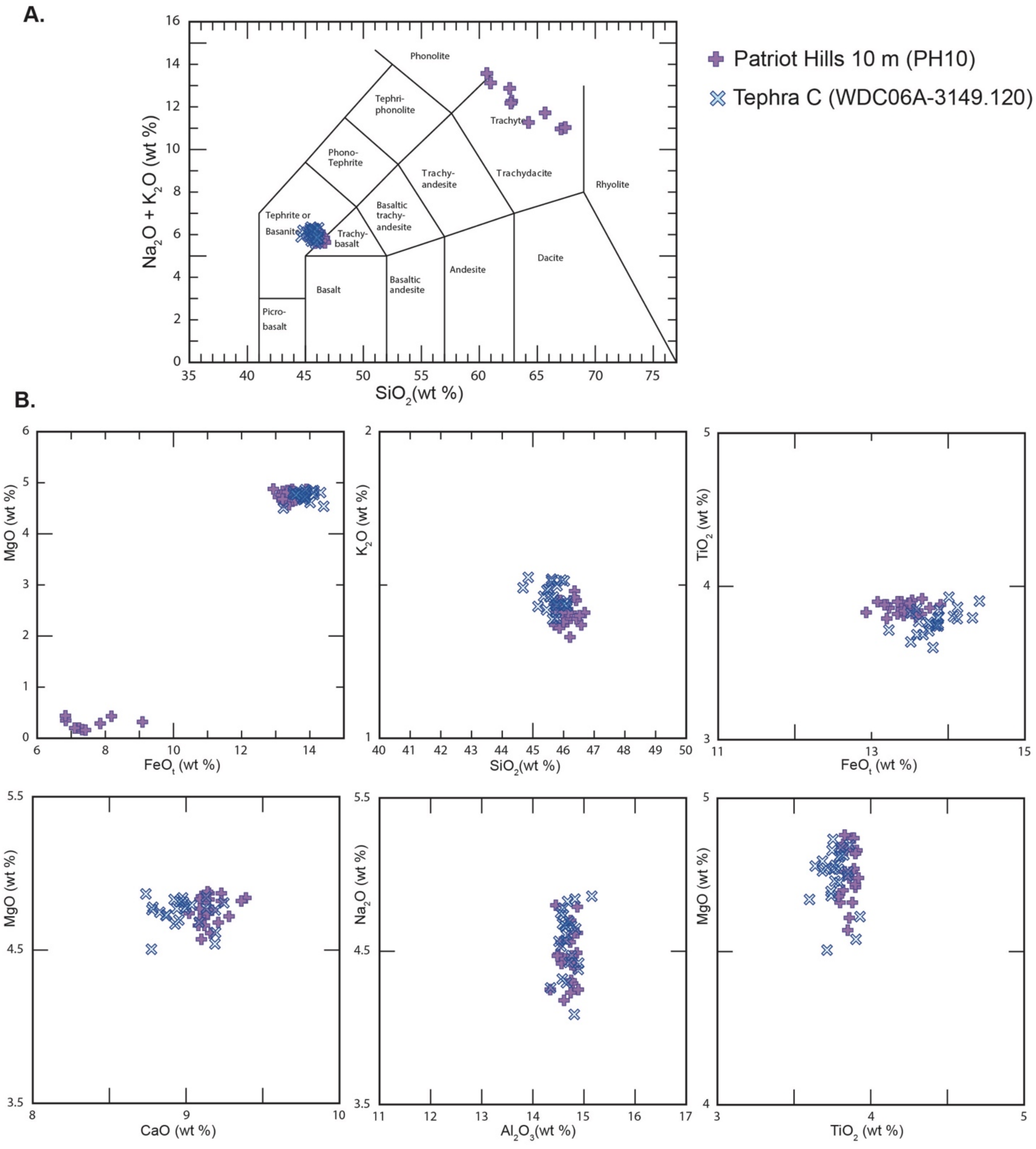

Fig. S10. Bivariate plots of Patriot Hills tephra 10 m and Tephra C identified in the WAIS Divide core (32). Panel A. Total alkali silica plot after Le Maitre (86). In the bivariate plots shown in Panel B, the axis is curtailed to illustrate the relationship between population 1 of PH 10 and Tephra C. All data are normalised to 100%; uncertainties on analyses are smaller than symbols.

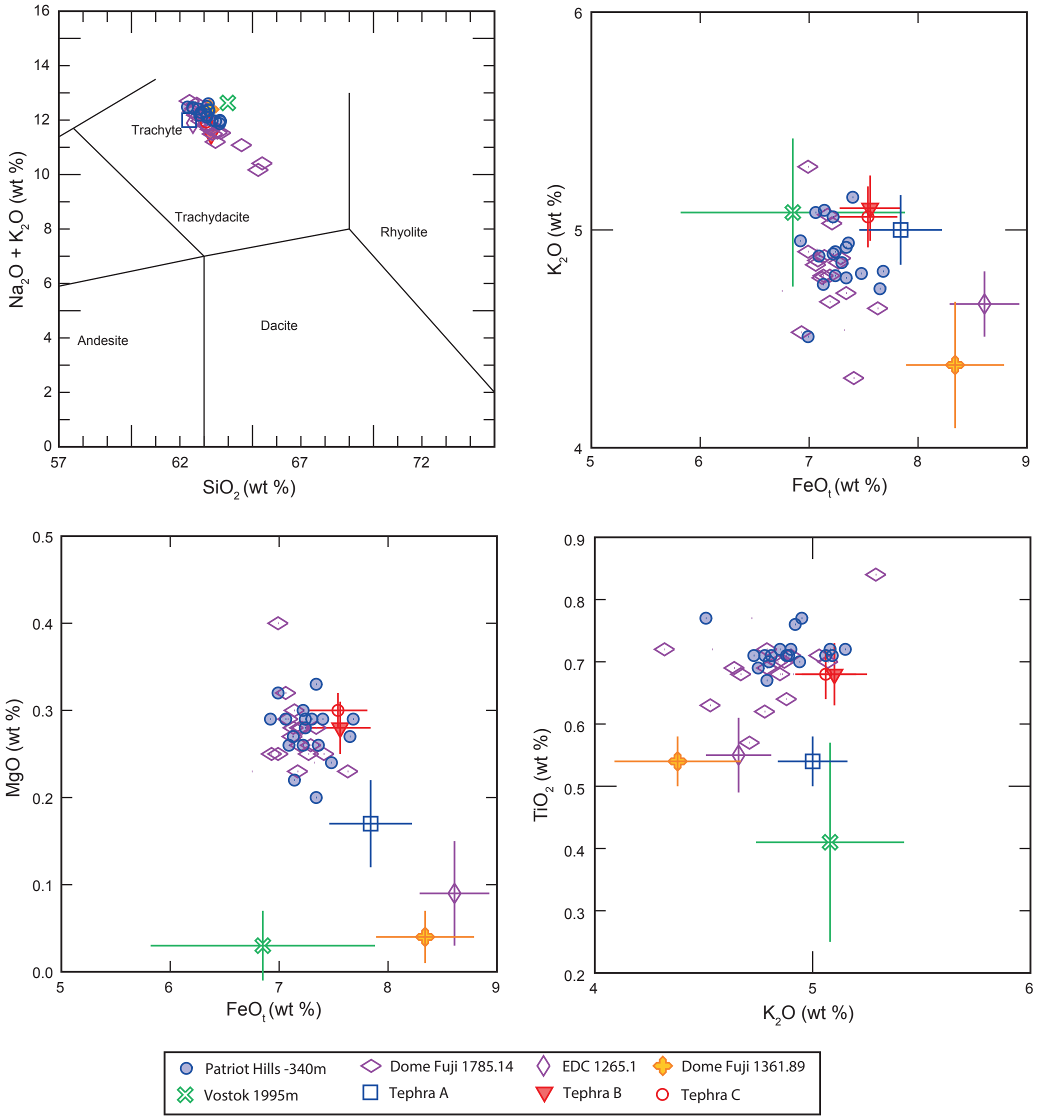

Fig. S11. Major element results for Patriot Hills tephra at -340 m. Single-shard analyses are plotted alongside the following ice-core tephras: Dome Fuji 1785.14 and 1361.89 (25), EDC 1265.1 (22, 24), Vostok 1995 m (30) and tephras in marine sediment cores from the West Antarctic continental margin: Tephra A, B and C (31). Mean and 1σ data are plotted for all populations, with the exception of the Patriot Hills -340 m tephra and the tephra from Dome Fuji at 1785.14 m depth. Single-shard analyses for the Dome Fuji 1785.14 m tephra are presented for the first time and microprobe operating conditions followed that of ref. (25). All data are normalised to 100%; uncertainties on individual analyses are smaller than symbols.

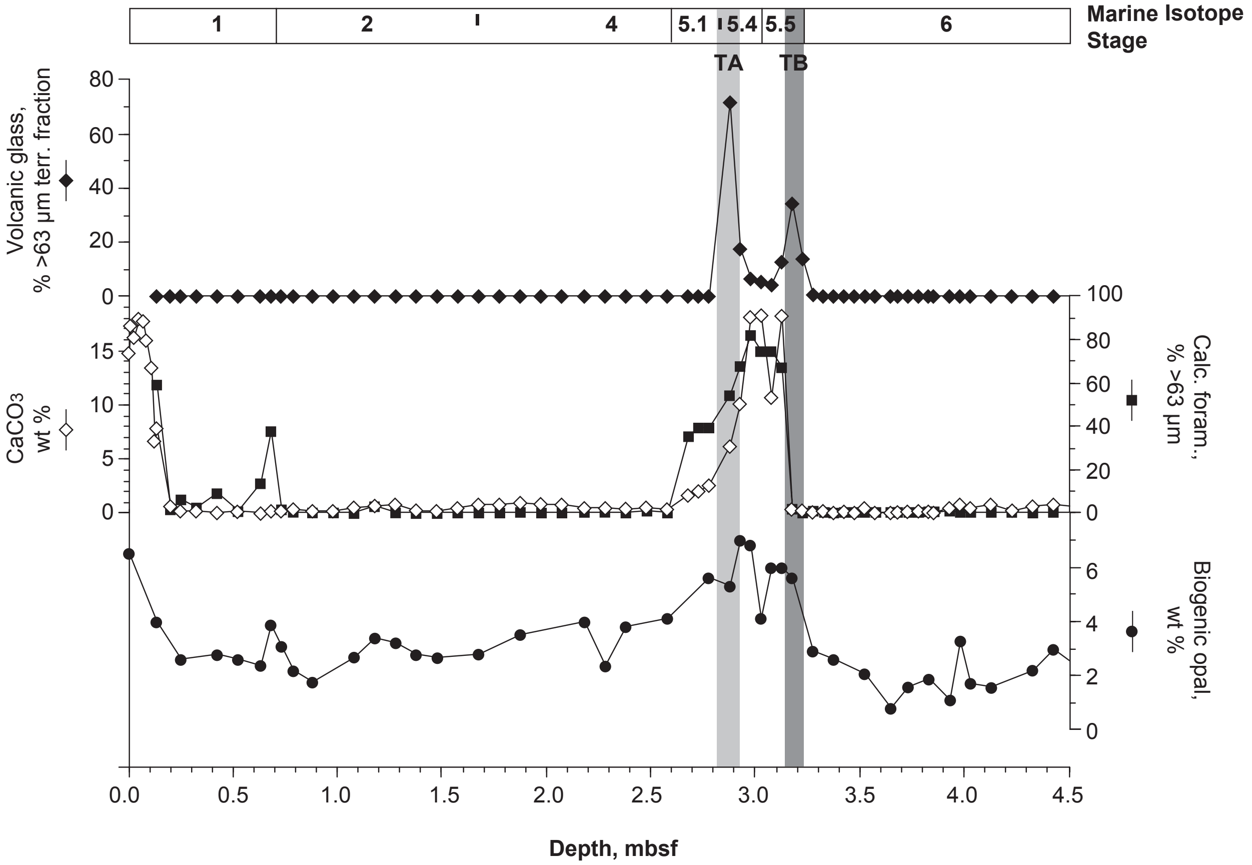

Fig. S12. Summary sedimentological record for marine sediment core PS2556 from the West Antarctic continental margin showing contents of biogenic opal (weight %), calcareous foraminifera (% of >63 µm), $CaCO_3$ (weight %), and volcanic glass (% of >63 µm in the terrigenous fraction) (modified from ref. (31)). 'TA' and 'TB' denote Tephras A (light grey column) and B (dark grey column) respectively.

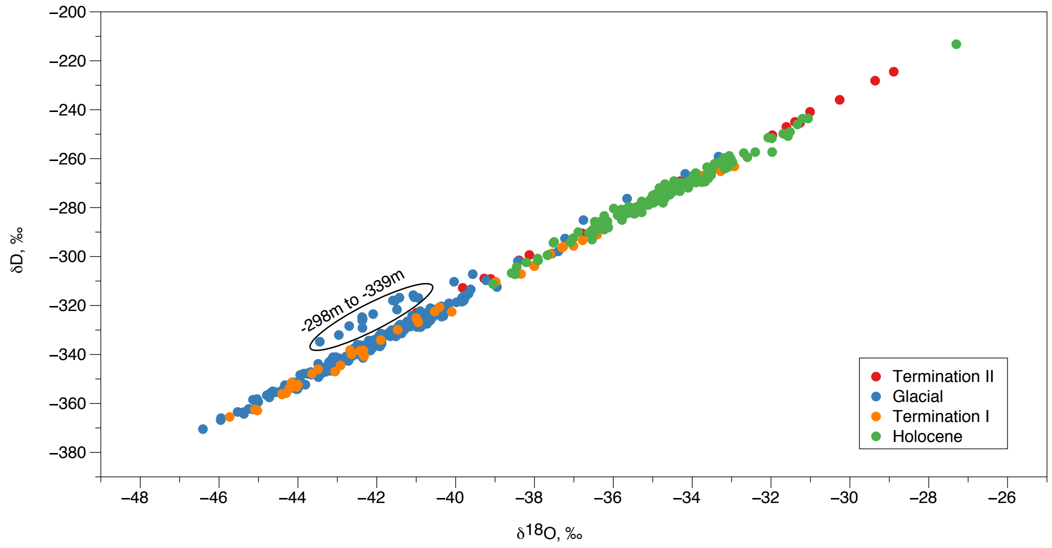

Fig. S13. Plot showing relationship between $\delta^{18}O$ and δD across the Patriot Hills blue ice area transect. Samples colour coded to their respective periods: Holocene, Termination I, the last glacial period, and Termination II (Fig. S5). Envelope denotes samples during the inferred resumption of ice accumulation at Patriot Hills (-298 to -339 m).

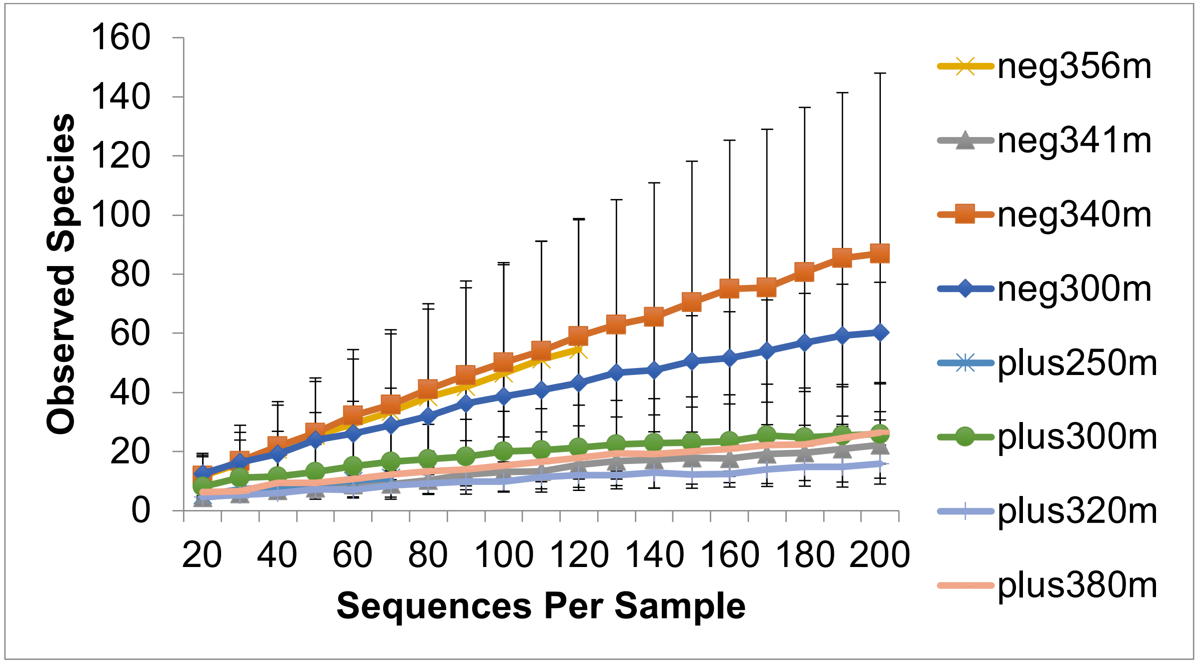

Fig. S14. Alpha rarefaction (observed species) calculated for 200 sequences within each filtered sample, Patriot Hills. Error bars represent the standard error calculated for each sampling depth. Note, the samples -356 and +250 m did not contain 200 filtered sequences, and therefore, all of their possible sequences were included.

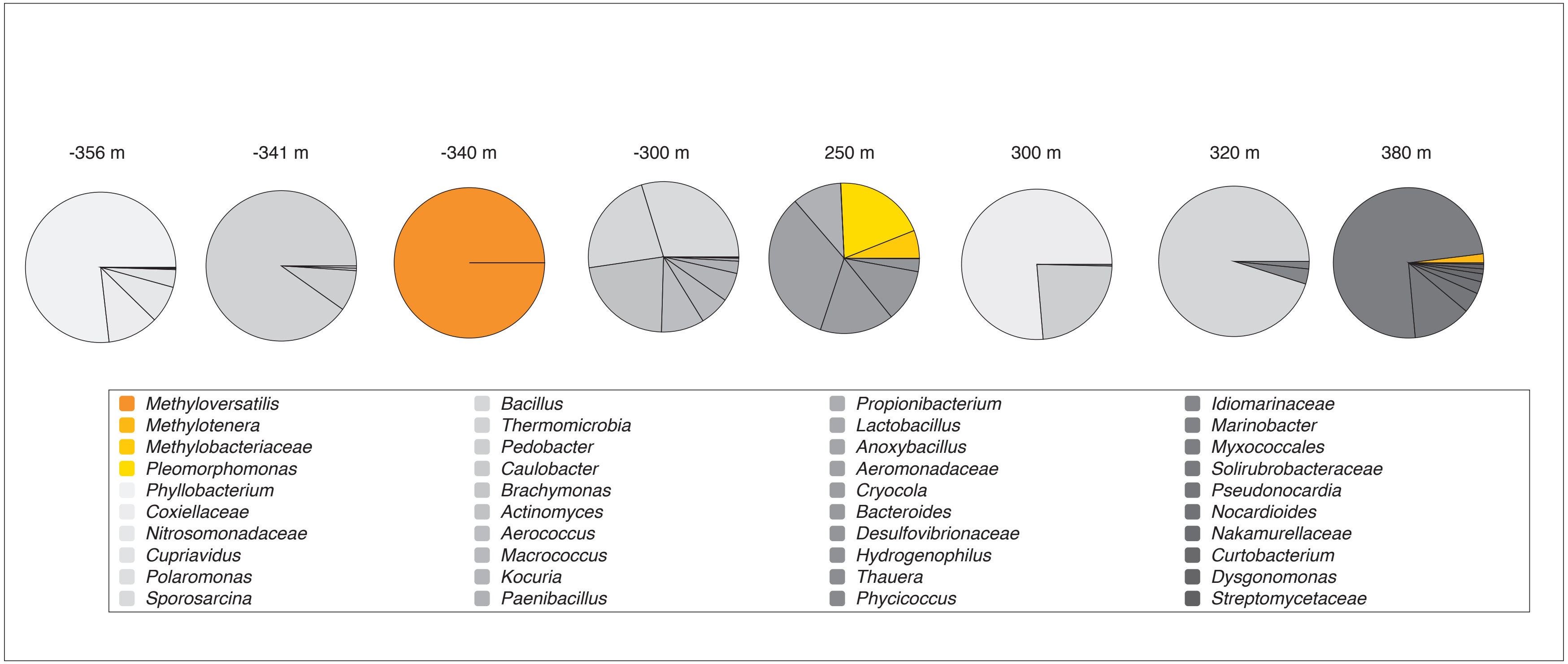

Fig. S15. Microbial genera detected in Patriot Hills ice using 16S displayed as a proportion of the total non-contaminant sequences in each Patriot Hills sample. Taxa not detected in sampling or laboratory controls. Organisms capable of utilizing single-carbon sources (*e.g.* methane) are highlighted in orange.

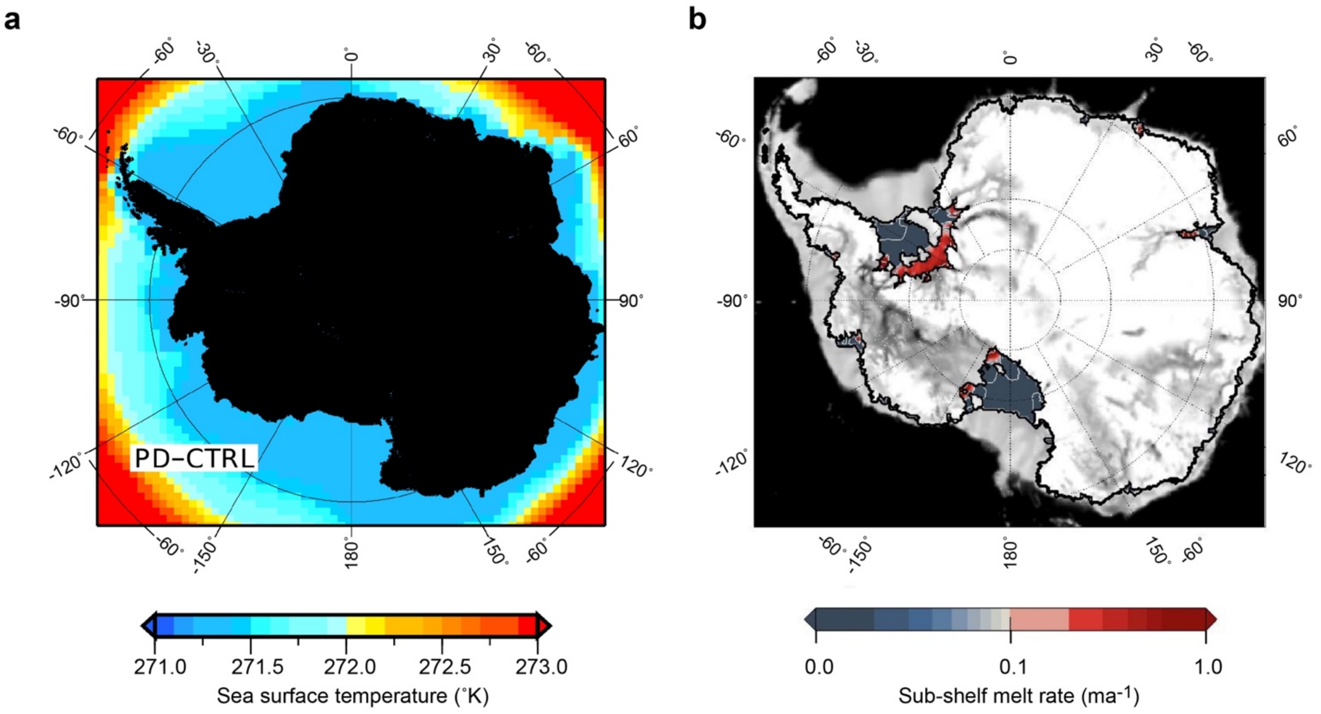

Fig. S16. Reference temperature and basal melt prior to modelling the impact of different ocean and atmospheric temperature scenarios. Sea surface temperature used for the "control" (CTRL) scenario, employing gridded data from observations and modelling of present-day conditions (modified from ref. (87)) (a). Modeled ice shelf melt rates under present-day conditions, used as the starting point for the model ensemble (b). Note: simulated melt rates are highest in the major embayments at deep grounding lines (modified from ref. (79)). The white contour line denotes boundary between zones of melt and zones of refreezing; grey shading over the Antarctic ice sheets defines areas of basal topography below present sea level.

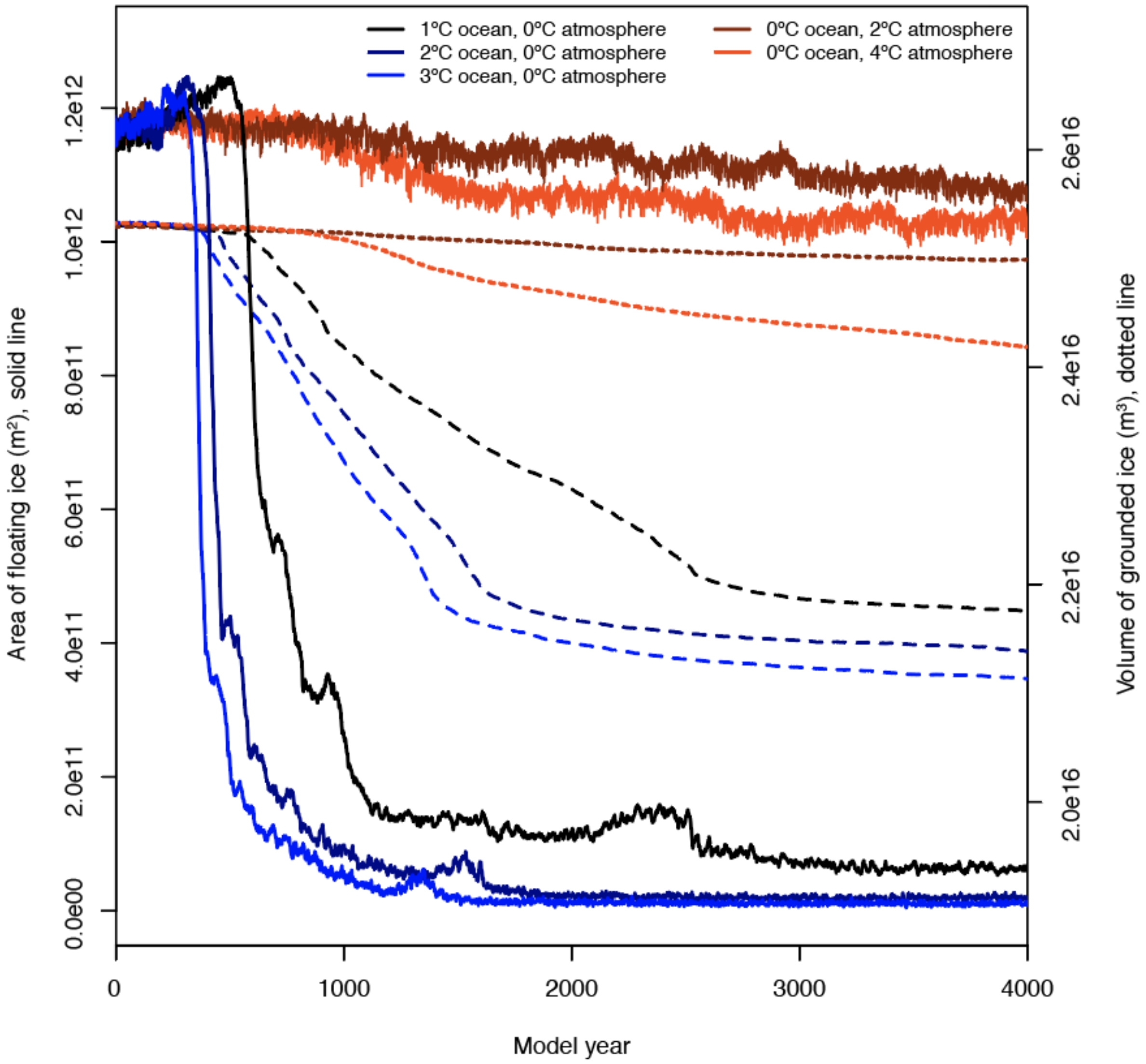

Fig. S17 Area of floating (solid line) and grounded (dashed line) ice for a combination of ocean and atmospheric warming scenarios comparable to that which may have been experienced during the Last Interglacial. The temperature increases are linearly scaled over the first millennium (0-1000 model years). Note, ocean warming has a considerably greater impact (both magnitude and rate of change) on Antarctic ice mass loss than atmosphere, even with only a 1˚C ocean temperature increase, and that grounded ice starts to decline within years to decades of the loss of the ice shelves. We consider the linear warming over a millennium to be conservative estimates of the rate of temperature increase during the LIG.

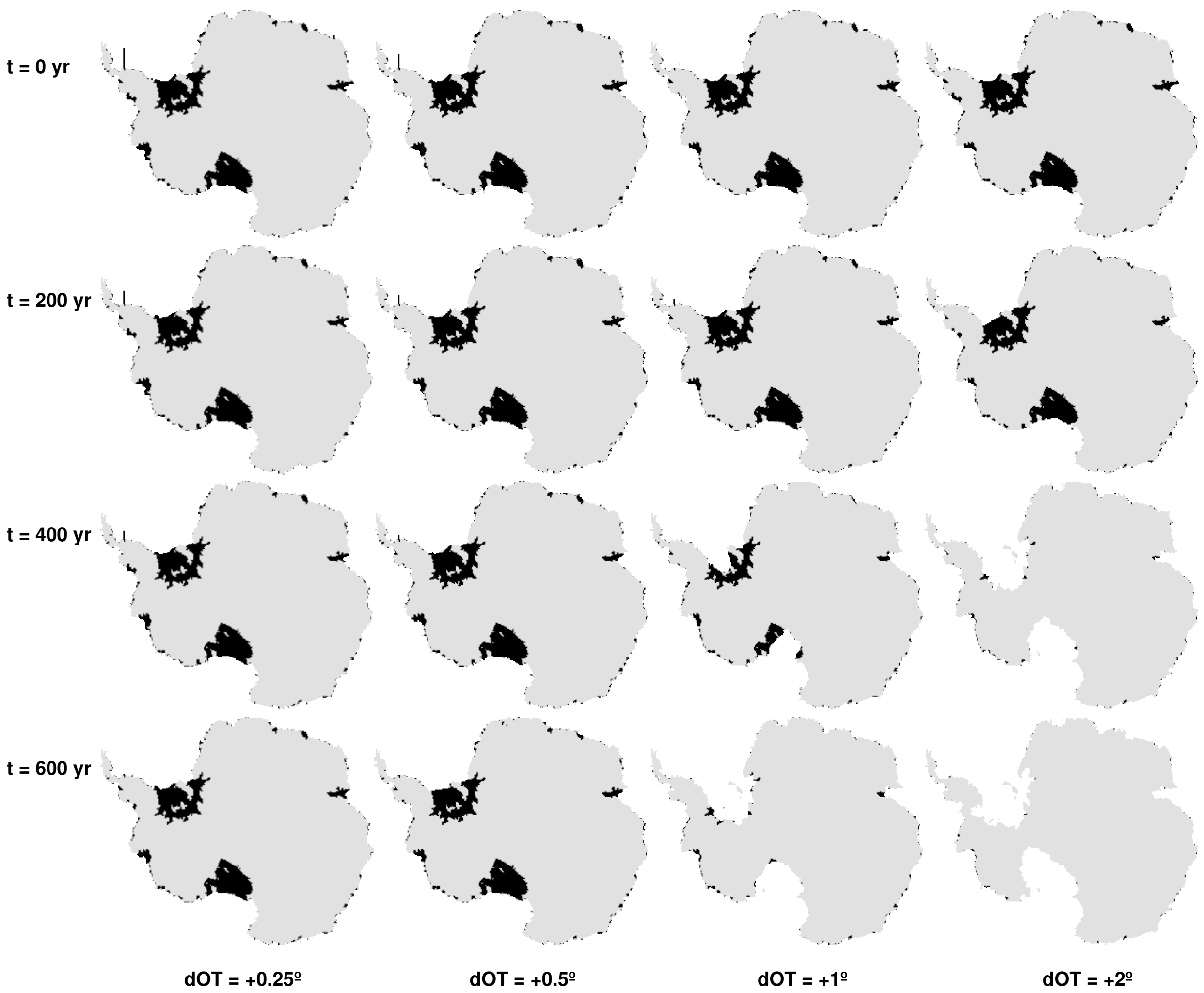

Fig. S18 Ice sheet (grey) and ice shelf (black) extent at 200-year intervals through the first 600 years of four simulations that use linearly increasing ocean forcing. No atmospheric warming above present is applied. Ocean temperature anomalies with respect to present are 0.25˚C, 0.5˚C, 1˚C, and 2˚C. Note that ice shelves retreat from their outer margins landward as ocean-driven thinning enhances calving at the ice edge.

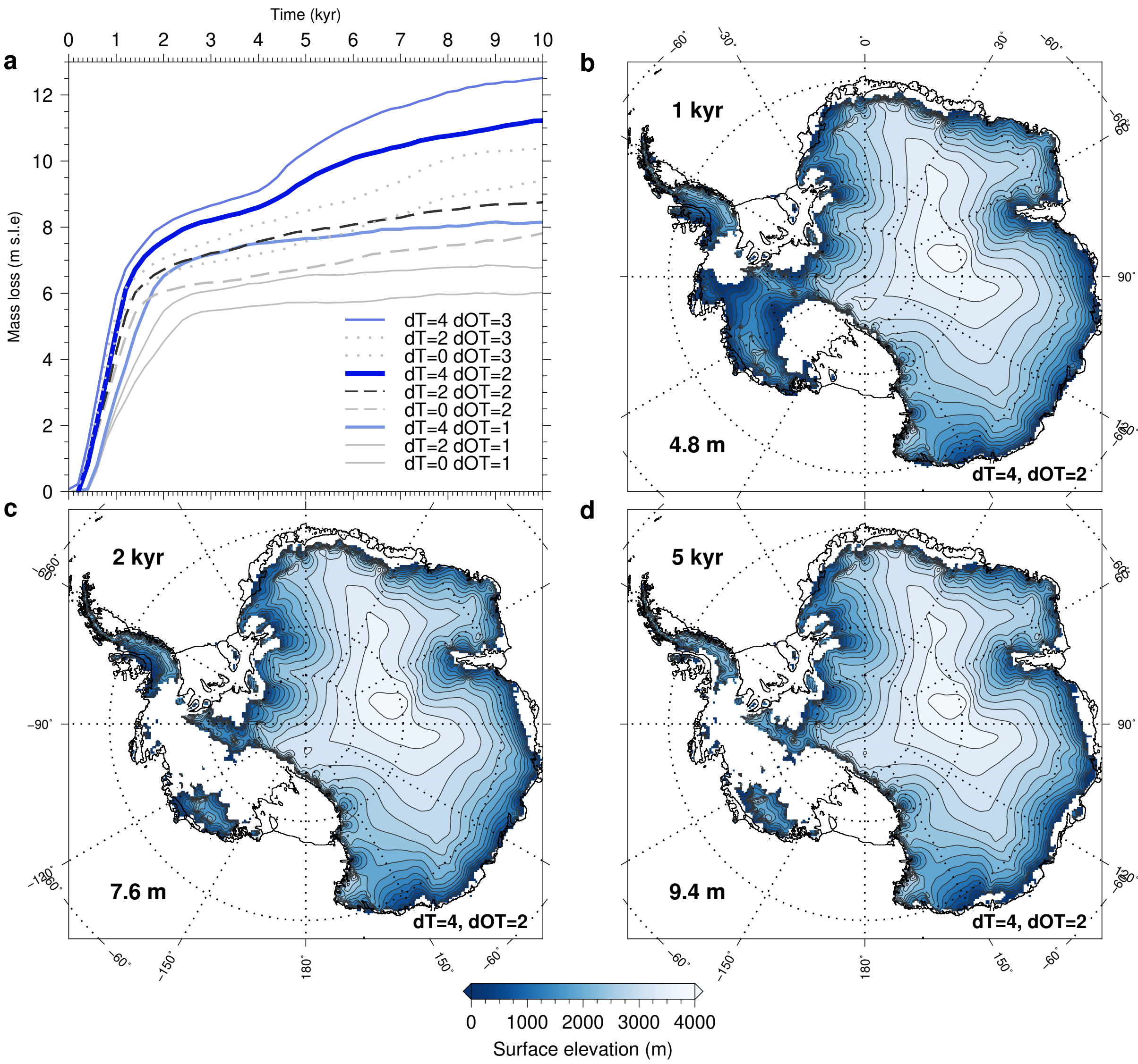

Fig. S19. Modelled Antarctic ice-sheet evolution under idealised forcing scenarios consistent with range of inferred Last Interglacial temperatures. (a). 'dT' and 'dOT' describes atmospheric and ocean temperature anomalies respectively. Antarctic Ice Sheet extent and elevation changes with 4˚C air and 2˚C ocean warming over 1, 2 and 5 kyr, respectively (b-d); equivalent sea-level contribution given in the bottom left corner of each panel.

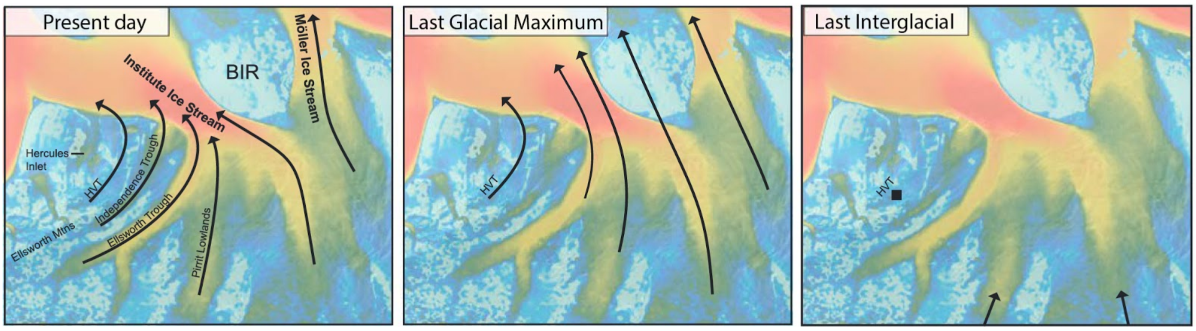

Fig. S20. MODIS satellite imagery (88) showing satellite-derived (present day) surface ice-flow velocities from MEaSUREs (89) and dominant ice streams. Last Glacial Maximum flow direction derived from Golledge *et al.* (90) and Last Interglacial inferred from present study. Horseshoe Valley Trough (HVT) and Bungenstock Ice Rise (BIR) abbreviated. Modified from Winter *et al.* (1).

Table S1 Single-shard analyses for Patriot Hills tephra deposits at -340 m and 10 m and Dome Fuji ice core at 1785.14 m depth. Data (weight %) are normalised to 100% and original totals are shown. n = number of tephra shards analysed from each sample. Secondary standard data for Lipari and BCR2G are shown and were analysed in relation to PH-340 m and PH10 during two analytical periods. Secondary standard data for Dome Fuji 1785.14 m can be found in (25). Recommended values used for Lipari (91) and BCR2G (92).

Sample	SiO_2	TiO_2	Al_2O_3	FeO_t	MnO	MgO	CaO	Na_2O	K_2O	P_2O_5	Total
PH 10 m	45.60	3.79	14.62	13.42	0.26	4.71	9.05	4.25	1.37	1.81	98.86
(brown	45.39	3.78	14.70	13.32	0.27	4.80	9.31	4.36	1.42	1.85	99.21
shards;	44.81	3.78	14.01	12.95	0.26	4.57	8.84	4.65	1.29	1.81	96.99
n=23)	45.13	3.82	14.38	13.68	0.26	4.80	9.09	4.12	1.43	1.76	98.46
	45.11	3.81	14.46	13.22	0.28	4.78	8.97	4.23	1.38	1.82	98.06
	45.43	3.75	14.35	12.87	0.26	4.49	8.91	4.11	1.37	1.77	97.31
	45.05	3.79	14.08	12.70	0.27	4.58	9.01	4.34	1.41	1.84	97.07
	45.38	3.75	14.28	13.09	0.27	4.76	8.93	4.47	1.38	1.82	98.12
	45.83	3.80	14.71	13.23	0.28	4.52	9.00	4.20	1.46	1.82	98.85
	44.55	3.76	14.36	13.41	0.26	4.62	8.79	4.58	1.33	1.79	97.46
	45.52	3.79	14.15	12.90	0.27	4.72	8.92	4.37	1.34	1.75	97.72
	44.85	3.75	14.54	13.13	0.25	4.72	8.89	4.52	1.33	1.78	97.77
	44.50	3.80	14.33	13.15	0.27	4.69	8.88	4.32	1.40	1.80	97.13
	44.51	3.78	14.25	12.99	0.27	4.67	8.95	4.44	1.37	1.78	97.03
	45.73	3.77	14.51	12.74	0.26	4.81	9.01	4.49	1.37	1.81	98.51
	45.63	3.80	14.28	12.92	0.27	4.67	9.00	4.34	1.38	1.79	98.08
	45.40	3.76	14.57	13.44	0.25	4.62	8.95	4.34	1.37	1.77	98.48
	45.04	3.80	14.53	13.26	0.26	4.57	8.95	4.53	1.42	1.77	98.14
	44.96	3.79	14.25	13.15	0.27	4.65	8.86	4.34	1.37	1.78	97.41
	45.42	3.84	14.05	13.39	0.28	4.65	8.96	4.17	1.43	1.82	98.00
	45.61	3.77	14.73	13.46	0.27	4.62	9.00	4.45	1.37	1.85	99.14
	44.60	3.81	14.38	13.00	0.26	4.70	9.13	4.36	1.38	1.84	97.46
	45.22	3.73	14.63	12.99	0.26	4.61	9.07	4.72	1.39	1.80	98.42
Mean	**45.19**	**3.78**	**14.40**	**13.15**	**0.27**	**4.67**	**8.98**	**4.38**	**1.38**	**1.80**	**97.99**
**stdev**	**0.41**	**0.03**	**0.21**	**0.25**	**0.01**	**0.09**	**0.11**	**0.16**	**0.04**	**0.03**	**0.68**
PH 10 m	58.99	0.81	13.21	8.79	0.41	0.31	1.42	7.91	4.79	0.10	96.75
(colour-	57.56	0.80	13.26	9.18	0.41	0.26	1.39	7.91	4.33	0.10	95.21
	61.44	0.92	12.61	7.82	0.33	0.41	1.19	6.50	4.27	0.13	95.64

less											
shards;											
n=10)	61.90	0.56	14.78	7.15	0.26	0.20	1.22	7.81	4.91	0.06	98.84
	60.29	0.60	16.23	6.80	0.26	0.34	1.29	8.54	4.94	0.09	99.39
	62.98	0.69	11.65	6.88	0.36	0.16	0.85	6.11	4.20	0.06	93.95
	63.82	0.68	11.33	7.02	0.35	0.15	0.83	6.14	4.31	0.04	94.68
	62.53	0.71	14.42	7.82	0.28	0.29	1.23	7.49	4.73	0.09	99.60
	62.94	0.73	12.61	6.81	0.34	0.19	0.93	6.48	4.76	0.06	95.84
	59.75	0.80	14.38	6.50	0.32	0.42	1.36	6.94	4.67	0.13	95.27
Mean	**61.22**	**0.73**	**13.45**	**7.48**	**0.33**	**0.27**	**1.17**	**7.18**	**4.59**	**0.09**	**96.52**
**stdev**	**2.01**	**0.11**	**1.51**	**0.91**	**0.05**	**0.10**	**0.22**	**0.86**	**0.28**	**0.03**	**2.05**
PH -340	62.32	0.71	14.80	7.68	0.29	0.29	1.36	7.67	4.81	0.08	100.18
m (n=18)	63.11	0.71	14.65	7.22	0.26	0.26	1.22	7.44	5.06	0.07	99.48
	63.48	0.70	14.51	7.48	0.30	0.24	1.27	7.16	4.80	0.07	100.53
	63.12	0.71	14.70	7.22	0.27	0.30	1.43	7.29	4.89	0.07	98.05
	63.66	0.72	14.54	7.24	0.27	0.28	1.23	7.09	4.90	0.08	92.86
	62.55	0.76	14.83	7.34	0.29	0.33	1.40	7.51	4.92	0.07	100.48
	62.82	0.71	14.56	7.65	0.29	0.27	1.44	7.45	4.73	0.08	100.34
	63.67	0.77	14.79	6.92	0.25	0.29	1.27	7.00	4.95	0.08	100.52
	63.33	0.69	14.85	7.13	0.28	0.27	1.37	7.24	4.75	0.08	99.68
	63.02	0.67	14.61	7.24	0.29	0.29	1.50	7.53	4.79	0.07	99.55
	63.19	0.71	14.72	7.09	0.26	0.26	1.33	7.50	4.88	0.07	99.44
	62.53	0.72	14.95	7.40	0.29	0.29	1.30	7.31	5.15	0.06	99.52
	63.65	0.70	14.46	7.36	0.28	0.26	1.37	6.93	4.94	0.06	99.46
	63.11	0.77	14.80	6.99	0.26	0.32	1.56	7.60	4.51	0.08	99.84
	63.21	0.72	14.73	7.06	0.26	0.29	1.33	7.27	5.08	0.06	99.59
	63.17	0.71	14.52	7.14	0.29	0.22	1.27	7.51	5.09	0.07	98.30
	62.77	0.72	14.80	7.30	0.30	0.29	1.35	7.56	4.85	0.07	100.25
	62.88	0.71	14.94	7.34	0.28	0.20	1.30	7.50	4.78	0.08	100.02
Mean	**63.09**	**0.72**	**14.71**	**7.27**	**0.28**	**0.28**	**1.35**	**7.36**	**4.88**	**0.07**	**99.34**
**stdev**	**0.39**	**0.02**	**0.15**	**0.20**	**0.01**	**0.03**	**0.09**	**0.21**	**0.16**	**0.01**	**1.80**
Dome	64.55	0.72	14.70	7.19	0.23	0.26	1.27	6.29	4.79	-	100.2
Fuji	65.40	0.63	14.91	6.93	0.30	0.25	1.17	5.88	4.53	-	99.75
1785.14	65.23	0.72	14.74	7.41	0.17	0.25	1.30	5.85	4.32	-	99.31
(n=17)	64.18	0.72	15.32	7.32	0.21	0.24	1.37	6.11	4.54	-	100.2
	63.55	0.70	15.38	7.06	0.07	0.32	1.38	6.70	4.84	-	100.1

	63.27	0.69	14.89	7.63	0.27	0.23	1.36	7.02	4.64	-	99.35
	62.39	0.70	15.36	7.17	0.24	0.23	1.20	7.63	5.07	-	100.6
	62.71	0.57	15.34	7.34	0.23	0.28	1.36	7.47	4.71	-	100.1
	63.68	0.71	15.31	6.99	0.26	0.25	1.21	6.63	4.90	0.05	100.6
	63.35	0.68	15.31	7.27	0.26	0.25	1.27	6.64	4.85	0.12	100.4
	62.63	0.70	15.09	7.29	0.29	0.26	1.24	7.56	4.87	0.06	100.7
	63.47	0.68	15.51	7.19	0.27	0.28	1.34	6.53	4.67	0.06	99.75
	62.89	0.69	15.38	7.13	0.27	0.28	1.30	7.18	4.79	0.11	100.2
	63.02	0.64	15.20	7.14	0.28	0.30	1.21	7.24	4.88	0.09	100.5
	63.08	0.71	15.27	7.21	0.29	0.28	1.24	6.79	5.03	0.09	99.80
	63.23	0.62	15.38	7.12	0.29	0.27	1.19	7.05	4.78	0.08	100.8
	62.53	0.84	16.03	6.99	0.25	0.40	0.50	7.03	5.29	0.12	100.7
Mean	**63.37**	**0.69**	**15.19**	**7.16**	**0.26**	**0.27**	**1.24**	**6.97**	**4.80**	**0.09**	**100.5**
Stdev	***0.94***	***0.06***	***0.31***	***0.19***	***0.06***	***0.04***	***0.18***	***0.68***	***0.21***	***0.02***	

Secondary standard data

Lipari (n=16)	74.16	0.08	13.04	1.57	0.07	0.05	0.75	4.14	5.22	0.00	99.08
st.dev	0.64	0.00	0.16	0.08	0.01	0.02	0.03	0.12	0.09	0.01	0.75
Recomm. range	*73.14-75.06*	*0.05-0.10*	*12.77-13.45*	*1.49-1.61*	*0.04-0.1*	*0.02-0.06*	*0.68-0.79*	*3.78-4.34*	*4.87-5.39*	*0.00-0.02*	
BCR2G (n=20)	54.37	2.28	13.36	12.44	0.20	3.60	7.22	3.19	1.82	0.32	98.80
st.dev	0.52	0.01	0.18	0.32	0.01	0.11	0.15	0.26	0.06	0.03	0.53
Recomm.	*54.1*	*2.26*	*13.5*	*12.42*	*n.d.*	*3.59*	*7.12*	*3.16*	*1.79*	*0.35*	
st.dev	*0.8*	*0.05*	*0.2*	*0.2*		*0.05*	*0.11*	*0.11*	*0.05*	*0.02*	

591

Table S2 Average trace element concentrations of volcanic glass shards from the Patriot Hills tephra at -340 m, and Tephra B from West Antarctic continental margin marine sediment cores PC108 (4.65 m depth) and PC111 (6.86 m depth) (31). MPI-DING reference glasses (42) were used to monitor analytical accuracy and provided in Data S1. The individual grain-specific concentrations of Patriot Hills tephra at -340 m and Tephra B are also reported.

**Tephra**	**PH340**		**Tephra B**		**Tephra B**	
**Locality**	**Patriot Hills**		**PC108-5A**		**PC111-8A**	
(ppm)	**Average**	**1σ**	**Average**	**1σ**	**Average**	**1σ**
Rb	146.4	25.3	157.4	9.8	156.9	7.8
Sr	20.0	11.9	12.1	9.8	10.4	0.6
Y	71.2	9.8	74.6	6.5	76.8	4.4
Zr	878.2	134.8	917.8	88.5	950.5	49.4
Nb	169.7	27.8	181.1	16.4	183.9	8.7
Ba	72.8	46.0	51.3	54.0	40.7	2.0
La	115.4	16.4	122.9	10.8	125.0	6.9
Ce	233.5	32.0	249.1	20.9	251.4	13.9
Pr	24.9	3.3	26.6	2.1	27.1	1.5
Nd	92.4	12.4	99.9	7.8	101.7	5.6
Sm	17.5	2.4	18.7	1.5	18.9	1.3
Eu	2.2	0.2	2.4	0.2	2.4	0.2
Gd	16.0	2.5	17.2	1.5	17.8	1.0
Dy	14.1	2.1	14.8	1.2	15.0	0.9
Er	7.7	1.2	8.1	0.8	8.2	0.4
Yb	7.2	1.2	7.8	0.7	8.1	0.6
Lu	1.1	0.2	1.1	0.1	1.2	0.1
Hf	19.9	3.2	20.9	2.2	21.5	1.2
Ta	9.7	1.6	10.4	1.0	10.7	0.6
Pb	18.1	2.8	19.6	3.2	19.3	1.5
Th	17.8	2.9	19.4	2.0	19.9	1.1
U	4.7	0.8	5.3	0.5	5.3	0.4
n	*19*		*34*		*12*	

Table S3 Chronology for the Patriot Hills Blue Ice Area (BIA). Estimated range using only trace gas measurements (CH_4, CO_2 and N_2O) and final Bayesian age modelled solutions using both trace gas data and geochemically-correlated tephra layers.

Dip-corrected distance, m (surface expression, m)	Tephra (kyr±1σ)	CH_4, ppb	1σ, ppb	CO_2 ppm	1σ, ppb	N_2O ppb	1σ, ppb	CO, ppb	1σ, ppb	*Gas age range and inferred best-fit in parentheses, yrs*	Bayesian age, yrs±1σ
1192.9 (1200)		676	30	283	0	264	8	77	2	*1 (930) 5000*	1270±540
992.8 (1000)		637	35	274	35	250	6	46	51	*1 (1180) 5000*	1650±410
792.8 (800)		595	38	275	1	270	1	74	14	*1 (2810) 5000*	2040±350
692.4 (700)		627	43	297	16	275	4	110	87	*No age solution*	2230±370
593.9 (600)		617	1	275	3	276	4	34	15	*No age solution*	2420±400
495.2 (500)		606	15	276	0	283	2	46	14	*5000 (5810) 11,000*	2610±460
394.8 (400)		579	11	271	1	275	1	80	4	*5000 (6030) 18,000*	2800±540

385.8 (390)		606	18	286	23	273	1	66	22	*5000 (8070) 18,000*	2820±550
376.1 (380)		605	22	274	21	276	2	40	4	*5000 (8170) 18,000*	2840±550
366 (370)		602	58	271	7	271	1	52	8	*5000 (9390) 18,000*	2860±560
356.8 (360)		610.6	11	272	0	272	0	64	4	*11,000 (11,540) 18,000*	10,620±370
336.8 (340)		527	11	249	1			61	4	*11,000 (11,780) 18,000*	12,350±370
315.5 (320)		558	22	242	1			170	4	*No age solution*	14,280±350
306 (310)		527	51	214	2	257	2	177	4	*13,000 (14,170) 18,000*	15,220±420
296 (300)		465	22	210	6					*13,000 (16,190) 18,000*	16,270±420
286 (290)		405	22	198	1			173	4	*13,000 (22,370) 115,000*	17,380±520
282	TD822a (17.6±0.7)(18)										17,850±580

279	WCM-93-25 (18.2±2.9)(19)										18,260±750
266.1 (270)		417	22	194	1	235	1	95	4	*18,000 (31,310) 115,000*	20,030±1660
246.2 (250)		518	29	214	4			126	72	*No age solution*	22,770±2750
195.2 (200)		456	28	200	4	236	2	85	13	*18,000 (32,380) 115,000*	28,450±1890
190	SDMA-5951c-1 (36.4±2.0)(20)										29,010±1830
93.7 (100)		504	9	224	10			90	16	*18,000 (33,840) 115,000*	37,520±950
10	WAIS Tephra C (44.9±0.3)(32)										44,930±320
5 (10)		476	38	209	2			153	14	*18,000 (33,860) 115,000*	45,420±430
-105.7 (-100)		539	3	223	2			82	12	*18,000 (35,600) 115,000*	56,470±2460
-205.6 (-200)		537	7	220	1			76	10	*18,000 (55,550) 115,000*	66,690±4410
-305.7 (-300)		701	7	225	11			519.1	0.1	*No age solution*	76,750±6130

-340	DF1785 (130.7±1.8)(25, 43)								130,310±1740
-345.6 (-341)		612	41	276	2	173	3	*115,000 (120,910) 130,000*	
-361.7 (-356)		648	10	305	3	277	14	*115,000 (128,680) 130,000*	

Table S4 The CQL code for the Bayesian (OxCal) age model used to generate the Patriot Hills BIA chronology presented in Figure 1B and Table S2.

```
Options()
 {
   Resolution=20;
  kIterations=500;
 };
  Plot()
  {
  var(f);
  var(t);
  f=0.3;
  t=2;
    Curve("AntCH4","AntCH4.14c");
    Curve("AntCO2","AntCO2.14c");
    Curve("AntN2O","AntN2O.14c");
  P_Sequence("",1,0.1)
   {
     Boundary(Top_Hat(calBP(133000),
4000))
   {
       z=-362;
   };
   C_Date("Dome Fuji TII tephra",
calBP(130700), 1800)
   {
    z=-340;
   };
   Boundary("Boundary")
   {
       z=-339;
   };
   Boundary("Boundary")
   {
       z=-338.5;
   };
Delta_R("uniform",U(-500,-200));
   Combine()
   {
       Curve("=AntCH4");
 Delta_R(86*f*T(t));
       R_Date(701,7);
       Curve("=AntCO2");
 Delta_R(29*f*T(t));
       R_Date(225,11);
       z=-305.7;
   };
   Combine()
   {
       Curve("=AntCH4");
 Delta_R(86*f*T(t));
       R_Date(537,7);
       Curve("=AntCO2");
 Delta_R(29*f*T(t));
       R_Date(220,1);
       z=-205.6;
   };
   Combine()
   {
       Curve("=AntCH4");
 Delta_R(86*f*T(t));
       R_Date(539,3);
       Curve("=AntCO2");
 Delta_R(29*f*T(t));
```

```
    R_Date(223,2);
    z=-105.7;
 };
   Combine()
 {
    Curve("=AntCH4");
  Delta_R(86*f*T(t));
    R_Date(476,38);
    Curve("=AntCO2");
  Delta_R(29*f*T(t));
    R_Date(209,2);
    z=5;
 };
 C_Date("WAIS Tephra C",
calBP(44865), 313)
 {
  z=10;
 };
   Combine()
 {
    Curve("=AntCH4");
  Delta_R(86*f*T(t));
    R_Date(504,9);
    Curve("=AntCO2");
  Delta_R(29*f*T(t));
    R_Date(224,10);
    z=93.7;
 };
 C_Date("SDMA-5951c-1",
calBP(36430), 2000)
 {
  z=190;
 };
   Combine()
 {
    Curve("=AntCH4");
  Delta_R(86*f*T(t));
    R_Date(456,28);
    Curve("=AntCO2");
  Delta_R(29*f*T(t));
    R_Date(200,4);
    Curve("=AntN2O");
  Delta_R(19*f*T(t));
    R_Date(236,2);
    z=195.2;
 };
   Combine()
 {
    Curve("=AntCH4");
  Delta_R(86*f*T(t));
    R_Date(518,29);
    Curve("=AntCO2");
  Delta_R(29*f*T(t));
    R_Date(214,4);
    z=246.2;
 };
 Boundary("Boundary")
 {
    z=247;
 };
 Delta_R("uniform",U(-500,-200));
   Combine()
 {
    Curve("=AntCH4");
  Delta_R(86*f*T(t));
    R_Date(417,22);
```

```
     Curve("=AntCO2");
   Delta_R(29*f*T(t));
     R_Date(194,1);
     Curve("=AntN2O");
   Delta_R(19*f*T(t));
     R_Date(235,1);
     z=266.1;
  };
  C_Date("WCM-93-25",
calBP(18200), 2900)
  {
   z=279;
  };
  C_Date("TD822a", calBP(17610),
730)
  {
   z=282;
  };
   Combine()
  {
     Curve("=AntCH4");
   Delta_R(86*f*T(t));
     R_Date(405,22);
     Curve("=AntCO2");
   Delta_R(29*f*T(t));
     R_Date(198,1);
     z=286;
  };
   Combine()
  {
     Curve("=AntCH4");
   Delta_R(86*f*T(t));
     R_Date(465,22);
     Curve("=AntCO2");
   Delta_R(29*f*T(t));
     R_Date(210,6);
     z=296;
  };
   Combine()
  {
     Curve("=AntCH4");
   Delta_R(86*f*T(t));
     R_Date(527,51);
     Curve("=AntCO2");
   Delta_R(29*f*T(t));
     R_Date(214,2);
     Curve("=AntN2O");
   Delta_R(19*f*T(t));
     R_Date(257,2);
     z=306;
  };
   Combine()
  {
     Curve("=AntCH4");
   Delta_R(86*f*T(t));
     R_Date(558,22);
     Curve("=AntCO2");
   Delta_R(29*f*T(t));
     R_Date(242,1);
     z=315.5;
  };
   Combine()
  {
     Curve("=AntCH4");
   Delta_R(86*f*T(t));
     R_Date(527,11);
```

```
     Curve("=AntCO2");
   Delta_R(29*f*T(t));
     R_Date(249,1);
     z=336.8;
  };
    Combine()
  {
     Curve("=AntCH4");
   Delta_R(86*f*T(t));
     R_Date(610.6,11);
     Curve("=AntCO2");
   Delta_R(29*f*T(t));
     R_Date(272,1);
     Curve("=AntN2O");
   Delta_R(19*f*T(t));
     R_Date(272,1);
     z=356.8;
  };

Boundary(Top_Hat(calBP(11650),2000)
)
  {
     z=360;
  };
  Boundary("Boundary")
  {
     z=361;
  };
  Delta_R("uniform",U(-300,-150));
    Combine()
  {
     Curve("=AntCH4");
   Delta_R(86*f*T(t));
     R_Date(602,58);
     Curve("=AntCO2");
   Delta_R(29*f*T(t));
     R_Date(271,7);
     Curve("=AntN2O");
   Delta_R(19*f*T(t));
     R_Date(271,1);
     z=366;
  };
    Combine()
  {
     Curve("=AntCH4");
   Delta_R(86*f*T(t));
     R_Date(605,22);
     Curve("=AntCO2");
   Delta_R(29*f*T(t));
     R_Date(274,21);
     Curve("=AntN2O");
   Delta_R(19*f*T(t));
     R_Date(276,2);
     z=376.1;
  };
    Combine()
  {
     Curve("=AntCH4");
   Delta_R(86*f*T(t));
     R_Date(606,18);
     Curve("=AntCO2");
   Delta_R(29*f*T(t));
     R_Date(286,23);
     Curve("=AntN2O");
   Delta_R(19*f*T(t));
     R_Date(273,1);
```

```
    z=385.8;
};
  Combine()
{
    Curve("=AntCH4");
 Delta_R(86*f*T(t));
    R_Date(579,11);
    Curve("=AntCO2");
 Delta_R(29*f*T(t));
    R_Date(271,1);
    Curve("=AntN2O");
 Delta_R(19*f*T(t));
    R_Date(275,1);
    z=394.8;
};
  Combine()
{
    Curve("=AntCH4");
 Delta_R(86*f*T(t));
    R_Date(606,15);
    Curve("=AntCO2");
 Delta_R(29*f*T(t));
    R_Date(276,1);
    Curve("=AntN2O");
 Delta_R(19*f*T(t));
    R_Date(283,2);
    z=495.2;
};
  Combine()
{
    Curve("=AntCH4");
 Delta_R(86*f*T(t));
    R_Date(617,1);
    Curve("=AntCO2");
 Delta_R(29*f*T(t));
    R_Date(275,3);
    Curve("=AntN2O");
 Delta_R(19*f*T(t));
    R_Date(276,4);
    z=593.9;
};
  Combine()
{
    Curve("=AntCH4");
 Delta_R(86*f*T(t));
    R_Date(627,43);
    Curve("=AntCO2");
 Delta_R(29*f*T(t));
    R_Date(297,16);
    Curve("=AntN2O");
 Delta_R(19*f*T(t));
    R_Date(275,4);
    z=692.4;
};
  Combine()
{
    Curve("=AntCH4");
 Delta_R(86*f*T(t));
    R_Date(595,38);
    Curve("=AntCO2");
 Delta_R(29*f*T(t));
    R_Date(275,1);
    Curve("=AntN2O");
 Delta_R(19*f*T(t));
    R_Date(270,1);
    z=792.8;
```

```
};
  Combine()
{
    Curve("=AntCH4");
 Delta_R(86*f*T(t));
    R_Date(637,35);
    Curve("=AntCO2");
 Delta_R(29*f*T(t));
    R_Date(274,35);
    Curve("=AntN2O");
 Delta_R(19*f*T(t));
    R_Date(250,6);
    z=992.8;
};
  Combine()
{
    Curve("=AntCH4");
 Delta_R(86*f*T(t));
    R_Date(676,30);
    Curve("=AntCO2");
 Delta_R(29*f*T(t));
    R_Date(283,1);
    Curve("=AntN2O");
 Delta_R(19*f*T(t));
    R_Date(264,8);
    z=1192.8;
};
  Boundary(Top_Hat(calBP(2000),2000))
{
    z=1200;
};
};
};
```

Table S5 DNA 16S sample information provided for each Patriot Hill blue ice sample at discrete positions along the transect as well as control samples. Total sequences in each sample before and after filtering of contaminant sequences are also included.

	Sequencin	BarcodeSequ	LinkerPrimer	Transect	Position	ExtractionMe	FilterSize	ACADID	SequencesPerS	SequencesP	ProportionSeque	Description
**plus380m**	E12	GATGTGGTG	GTACACACC	BlueIce	plus380m	CTAB	0.45	17057A	151915	15180	0.100	Sample
**plus380m**	Well9E	TGCCGTATG	GTACACACC	BlueIce	plus380m	Powerlyzer	0.45	17067B	64365	25969	0.403	Sample
**plus320m**	H3	GCGACAATT	GTACACACC	BlueIce	plus320m	Powerlyzer	0.45	17070A	46617	10526	0.226	Sample
**plus320m**	H6	GAGAGCAAC	GTACACACC	BlueIce	plus320m	CTAB	0.45	17070B	27330	7687	0.281	Sample
**plus300m**	H2	TCACCTCCT	GTACACACC	BlueIce	plus300m	Powerlyzer	0.45	17056A	97190	46999	0.484	Sample
**plus300m**	H5	AGCTGTCAA	GTACACACC	BlueIce	plus300m	CTAB	0.45	17056B	18730	6327	0.338	Sample
**plus250m**	D4	AGCAACATT	GTACACACC	BlueIce	plus250m	CTAB	0.45	17055A	22694	4470	0.197	Sample
**plus250m**	Well9H	TTAAGACAG	GTACACACC	BlueIce	plus250m	Powerlyzer	0.45	17057B	170769	80339	0.470	Sample
**neg356m**	D2	GCCAACAAC	GTACACACC	BlueIce	neg356m	CTAB	0.45	17048A	32430	7119	0.220	Sample
**neg356m**	Well9B	TGGCAAATC	GTACACACC	BlueIce	neg356m	Powerlyzer	0.45	17048B	119449	54053	0.453	Sample
**neg341m**	E10	ACCCAAGCC	GTACACACC	BlueIce	neg341m	CTAB	0.45	17067A	27594	4168	0.151	Sample
**neg341m**	Well9F	CGTGACAAT	GTACACACC	BlueIce	neg341m	Powerlyzer	0.45	17247B	127108	54803	0.431	Sample
**neg340m**	E9	TCCTCGAGC	GTACACACC	BlueIce	neg340m	CTAB	0.45	17066A	114885	16303	0.142	Sample
**neg340m**	Well9D	TTAACCTTC	GTACACACC	BlueIce	neg340m	Powerlyzer	0.45	17066B	177816	91618	0.515	Sample
**neg300m**	E8	ATCAGTACT	GTACACACC	BlueIce	neg300m	CTAB	0.45	17049A	252580	43304	0.171	Sample
**neg300m**	Well9C	CACCTTACC	GTACACACC	BlueIce	neg300m	Powerlyzer	0.45	17049B	134118	47121	0.351	Sample
**ControlWell9**	Well9A	ACACGCGG	GTACACACC	BlueIce	Control	Powerlyzer	NA	17246B	70083	28711	0.410	EBC
**ControlH1**	H1	ATCTACCGA	GTACACACC	BlueIce	Control	Powerlyzer	NA	17508	18794	5336	0.284	EBC
**ControlG1**	G11	TATGTGCCG	GTACACACC	BlueIce	Control	Powerlyzer	NA	17248B	148467	116	0.001	EBC
**ControlE15**	E15	CGTGATCCG	GTACACACC	BlueIce	Control	CTAB	NA	17248A	251446	30329	0.121	EBC
**ControlE1**	E1	ACAACACTC	GTACACACC	BlueIce	Control	N/A	NA	17227	64193	13660	0.213	EBC
**ControlD1**	D1	GACATTGTC	GTACACACC	BlueIce	Control	CTAB	NA	17246	26469	4336	0.164	EBC

Table S6 Bacterial taxa identified within DNA 16S control samples.

	ContorlD1	ControlE1	ControlE15	ControlG1	ControlH1	ControlWell9
**Porphyromonas**	0	0	0	0	383	0
**Prevotella**	0	0	0	0	0	445
**Sphingobacterium**	0	0	0	0	100	0
**Fusobacterium**	0	0	0	1	0	0
**Brevundimonas**	1	0	0	0	0	0
**Bradyrhizobium**	176	3007	736	3	0	22
**Mycoplana**	0	1	123	0	0	1
**Rhodoplanes**	0	1	0	0	0	0
**Methylobacterium**	0	0	0	1	850	54
**Mesorhizobium**	1	0	0	0	0	314
**Agrobacterium**	0	4	262	0	0	0
**Paracoccus**	0	0	0	2	0	0
**Novosphingobium**	0	55	10	2	0	428
**Sphingobium**	0	0	87	4	0	692
**Sphingomonas**	0	4	566	6	0	43
**Sphingopyxis**	0	0	0	1	0	714
**Azohydromonas**	0	0	2	0	0	0
**Burkholderia**	0	0	0	1	0	0
**Lautropia**	0	0	1	0	0	0
**Ralstonia**	0	1033	991	1	2115	110
**Acidovorax**	1	2	12	23	0	12491
**Comamonas**	5	14143	44899	8	1038	1722
**Delftia**	0	0	0	0	0	10
**Hydrogenophaga**	0	0	1	0	0	0
**Hylemonella**	0	637	0	0	0	0
**Limnohabitans**	0	561	1	11	0	6498
**Ramlibacter**	0	0	0	0	0	1
**Herbaspirillum**	0	0	3	0	0	0
**Janthinobacterium**	604	222	302	5	12	552
**Rubrivivax**	0	0	1	0	0	0
**Tepidimonas**	11	1	10	3	0	346
**Dechloromonas**	1	0	0	0	0	0
**Zoogloea**	0	0	8	0	73	5
**Denitrobacter**	0	0	0	0	784	0
**Citrobacter**	0	8	4	0	0	0
**Klebsiella**	0	1	0	0	0	0
**Legionella**	0	0	0	0	0	1
**Alcanivorax**	0	0	0	0	1	0
**Halomonas**	0	0	0	0	2	172
**Acinetobacter**	7003	23696	156138	20	4003	10537
**Pseudomonas**	14326	7006	15088	19	4038	4318
**Lysobacter**	0	3	0	0	0	0
**Pseudoxanthomonas**	0	0	2	0	0	319
**Stenotrophomonas**	0	0	889	2	0	0
**Thermomonas**	0	1	0	0	0	0
**Actinomycetales**	0	1	972	15	0	1572
**Alicyclobacillus**	0	0	1	0	0	0
**Staphylococcus**	2	0	8	6	0	4
**Streptococcus**	0	0	0	0	59	0
**Total Sequences Per San**	22131	50387	221117	134	13458	41371

Table S7 Selected parameters using the Parallel Ice Sheet Model v.0.6.3.

Parameter	Value	Units
Resolution (*x,y*)	20	km
Ice grid resolution (*z*)	0.024	km
Bedrock grid resolution (*z*)	0.1	km
Air temperature lapse rate (*L*)	-8	˚K km^{-1}
Relative precipitation change with air temperature (f_p)	7.3	% K^{-1}
SIA enhancement (E_{SIA})	1.2	
SSA enhancement (E_{SSA})	0.5	
Maximum water thickness in till (W_{max})	2	m
Eigen calving coefficient	5e+17	
Thickness calving limit (H_{cr})	220	m
Density of lithosphere (ρ)	3300	kg m^{-3}
Viscosity of mantle (η)	1 x 10^{20}	Pa s

References

1. Winter K*, et al.* (2015) Airborne radar evidence for tributary flow switching in Institute Ice Stream, West Antarctica: implications for ice sheet configuration and dynamics. *Journal of Geophysical Research: Earth Surface* 120(9):1611-1625.
2. Bingham RG*, et al.* (2015) Ice-flow structure and ice dynamic changes in the Weddell Sea sector of West Antarctica from radar-imaged internal layering. *Journal of Geophysical Research: Earth Surface* 120(4):655-670.
3. Winter K*, et al.* (2016) Assessing the continuity of the blue ice climate record at Patriot Hills, Horseshoe Valley, West Antarctica. *GRL* 43(5):2019-2026.
4. Rignot E, Mouginot J, & Scheuchl B (2017) MEaSUREs InSAR-Based Antarctica Ice Velocity Map, Version 2. ed NASA National Snow and Ice Data Center Distributed Active Archive Center (Boulder, Colorado, USA).
5. Casassa G*, et al.* (2004) Elevation change and ice flow at Horseshoe Valley, Patriot Hills, West Antarctica. *Annals of Glaciology* 39(1):20-28.
6. Jeofry H*, et al.* (2018) A new bed elevation model for the Weddell Sea sector of the West Antarctic Ice Sheet. *Earth System Science Data* 10(2):711-725.
7. Fogwill CJ*, et al.* (2017) Antarctic ice sheet discharge driven by atmosphere-ocean feedbacks at the Last Glacial Termination. *Sci Rep* 7:39979, doi: 39910.31038/srep39979.
8. Turney C*, et al.* (2013) Late Pleistocene and early Holocene change in the Weddell Sea: a new climate record from the Patriot Hills, Ellsworth Mountains, West Antarctica. *J Quatern Sci* 28(7):697-704.
9. Hein AS*, et al.* (2016) Evidence for the stability of the West Antarctic Ice Sheet divide for 1.4 million years. *Nature Comms* 7:10325.

10. Fogwill CJ*, et al.* (2014) Drivers of abrupt Holocene shifts in West Antarctic ice stream direction determined from combined ice sheet modelling and geologic signatures. *Antarctic Science* 26(Special Issue 06):674-686.

11. Montagnat M*, et al.* (2014) Fabric along the NEEM ice core, Greenland, and its comparison with GRIP and NGRIP ice cores. *The Cryosphere* 8(4):1129-1138.

12. Etheridge DM*, et al.* (1996) Natural and anthropogenic changes in atmospheric CO_2 over the last 1000 years from air in Antarctic ice and firn. *Journal of Geophysical Research: Atmospheres* 101(D2):4115-4128.

13. Rubino M*, et al.* (2013) A revised 1000 year atmospheric $\delta^{13}C$-CO_2 record from Law Dome and South Pole, Antarctica. *Journal of Geophysical Research* 118:1-18.

14. Francey R*, et al.* (2003) The CSIRO (Australia) measurement of greenhouse gases in the global atmosphere. *Baseline Atmospheric Program Australia 1999-2000*, eds Tindale N, Derek N, & Fraser P (Bureau of Meteorology and CSIRO Atmospheric Research, Melbourne), pp 42-53.

15. Köhler P, Nehrbass-Ahles C, Schmitt J, Stocker TF, & Fischer H (2017) A 156 kyr smoothed history of the atmospheric greenhouse gases CO_2, CH_4, and N_2O and their radiative forcing. *Earth Syst. Sci. Data* 9(1):363-387.

16. Wadham JL*, et al.* (2012) Potential methane reservoirs beneath Antarctica. *Nature* 488(7413):633-637.

17. NEEM Community Members (2013) Eemian interglacial reconstructed from a Greenland folded ice core. *Nature* 493(7433):489-494.

18. Narcisi B, Petit JR, & Langone A (2017) Last glacial tephra layers in the Talos Dome ice core (peripheral East Antarctic Plateau), with implications for chronostratigraphic correlations and regional volcanic history. *Quatern Sci Rev* 165:111-126.

19. Wilch TI, McIntosh W, & Dunbar N (1999) Late Quaternary volcanic activity in Marie Byrd Land: Potential ^{40}Ar/^{39}Ar-dated time horizons in West Antarctic ice and marine cores. *Geological Society of America Bulletin* 111(10):1563-1580.

20. Dunbar NW & Kurbatov AV (2011) Tephrochronology of the Siple Dome ice core, West Antarctica: correlations and sources. *Quatern Sci Rev* 30(13–14):1602-1614.

21. Hayward C (2012) High spatial resolution electron probe microanalysis of tephras and melt inclusions without beam-induced chemical modification. *Holocene* 22(1):119-125.

22. Narcisi B, Petit JR, Delmonte B, Basile-Doelsch I, & Maggi V (2005) Characteristics and sources of tephra layers in the EPICA-Dome C ice record (East Antarctica): Implications for past atmospheric circulation and ice core stratigraphic correlations. *EPSL* 239(3–4):253-265.

23. Narcisi B, Petit JR, & Delmonte B (2010) Extended East Antarctic ice-core tephrostratigraphy. *Quaternary Science Reviews* 29(1-2):21-27.

24. Narcisi B, Petit JR, & Tiepolo M (2006) A volcanic marker (92 ka) for dating deep east Antarctic ice cores. *Quaternary Science Reviews* 25(21-22):2682-2687.

25. Kohno M, Fujii Y, & Hirata T (2004) Chemical composition of volcanic glasses in visible tephra layers found in a 2503 m deep ice core from Dome Fuji, Antarctica. *Annals of Glaciology* 39(1):576-584.

26. Dunbar NW, McIntosh WC, & Esser RP (2008) Physical setting and tephrochronology of the summit caldera ice record at Mount Moulton, West Antarctica. *Geological Society of America Bulletin* 120(7-8):796-812.

27. Narcisi B, Petit JR, Langone A, & Stenni B (2016) A new Eemian record of Antarctic tephra layers retrieved from the Talos Dome ice core (Northern Victoria Land). *Global and Planetary Change* 137:69-78.

28. Narcisi B, Petit JR, Delmonte B, Scarchilli C, & Stenni B (2012) A 16,000-yr tephra framework for the Antarctic ice sheet: a contribution from the new Talos Dome core. *Quatern Sci Rev* 49:52-63.

29. Narcisi B, Petit JR, & Chappellaz J (2010) A 70 ka record of explosive eruptions from the TALDICE ice core (Talos Dome, East Antarctic plateau). *Journal of Quaternary Science* 25(6):844-849.

30. Basile I, Petit JR, Touron S, Grousset FE, & Barkov N (2001) Volcanic layers in Antarctic (Vostok) ice cores: Source identification and atmospheric implications. *Journal of Geophysical Research-Atmospheres* 106(D23):31915-31931.

31. Hillenbrand C-D*, et al.* (2008) Volcanic time-markers for Marine Isotopic Stages 6 and 5 in Southern Ocean sediments and Antarctic ice cores: implications for tephra correlations between palaeoclimatic records. *Quatern Sci Rev* 27(5):518-540.

32. Iverson NA*, et al.* (2017) The first physical evidence of subglacial volcanism under the West Antarctic Ice Sheet. *Sci Rep* 7(1):11457.

33. Borchardt G, Aruscavage P, & Millard HJ (1972) Correlation of the Bishop ash, a Pleistocene marker bed, using instrumental neutron activation analysis. *Journal of Sedimentary Petrology* 42:301-306.

34. Kawamura K*, et al.* (2007) Northern Hemisphere forcing of climatic cycles in Antarctica over the past 360,000 years. *Nature* 448:912–916.

35. Uemura R, Yoshida N, Kurita N, Nakawo M, & Watanabe O (2004) An observation-based method for reconstructing ocean surface changes using a 340,000-year deuterium excess record from the Dome Fuji ice core, Antarctica. *GRL* 31(13):n/a-n/a.

36. Veres D*, et al.* (2013) The Antarctic ice core chronology (AICC2012): an optimized multi-parameter and multi-site dating approach for the last 120 thousand years. *Clim Past* 9(4):1733-1748.

37. Bonn WJ, Gingele FX, Grobe H, Mackensen A, & Fütterer DK (1998) Palaeoproductivity at the Antarctic continental margin: opal and barium records for the last 400 ka. *Palaeogeography, Palaeoclimatology, Palaeoecology* 139(3):195-211.

38. Hillenbrand C-D & Cortese G (2006) Polar stratification: a critical view from the Southern Ocean. *Palaeogeography, Palaeoclimatology, Palaeoecology* 242(3-4):240-252.

39. Grobe H & Mackensen A (1992) Late Quaternary climatic cycles as recorded in sediments from the Antarctic continental margin. *The Antarctic paleoenvironment: A perspective on Global Change; Antarctic Research Series* 56:349-376.

40. Müller W, Shelley M, Miller P, & Broude S (2009) Initial performance metrics of a new custom-designed ArF excimer LA-ICPMS system coupled to a two-volume laser-ablation cell. *Journal of Analytical Atomic Spectrometry* 24(2):209-214.

41. Tomlinson E, Thordarson T, Müller W, Thirlwall M, & Menzies M (2010) Microanalysis of tephra by LA-ICP-MS—strategies, advantages and limitations assessed using the Thorsmörk Ignimbrite (Southern Iceland). *Chemical Geology* 279(3):73-89.

42. Jochum KP*, et al.* (2006) MPI-DING reference glasses for *in situ* microanalysis: New reference values for element concentrations and isotope ratios. *Geochemistry, Geophysics, Geosystems* 7(2):doi: 10.1029/2005GC001060.

43. Fujita S, Parrenin F, Severi M, Motoyama H, & Wolff E (2015) Volcanic synchronization of Dome Fuji and Dome C Antarctic deep ice cores over the past 216 kyr. *Clim Past* 11:1395-1416.

44. Bronk Ramsey C & Lee S (2013) Recent and planned developments of the program OxCal. *Radiocarbon* 55(2-3):720-730.

45. Bronk Ramsey C (2007) Deposition models for chronological records. *Quatern Sci Rev* 27:42-60.

46. Bronk Ramsey C (2009) Dealing with outliers and offsets in radiocarbon dating. *Radiocarbon* 51(3):1023-1045.

47. Higham T*, et al.* (2014) The timing and spatiotemporal patterning of Neanderthal disappearance. *Nature* 512(7514):306-309.

48. Bronk Ramsey C (1998) Probability and dating. *Radiocarbon* 40:461-474.

49. Walker M*, et al.* (2009) Formal definition and dating of the GSSP (Global Stratotype Section and Point) for the base of the Holocene using the Greenland NGRIP ice core, and selected auxiliary records. *J Quatern Sci* 24:3-17.

50. Buizert C*, et al.* (2015) The WAIS Divide deep ice core WD2014 chronology; Part 1: Methane synchronization (68–31 ka BP) and the gas age–ice age difference. *Climates of the Past* 11(2):153-173.

51. Munksgaard NC, Wurster CM, & Bird MI (2011) Continuous analysis of $\delta^{18}O$ and δD values of water by diffusion sampling cavity ring-down spectrometry: A novel sampling device for unattended field monitoring of precipitation, ground and surface waters. *Rapid Communications in Mass Spectrometry* 25:3706-3712.

52. Santibáñez PA*, et al.* (2018) Prokaryotes in the WAIS Divide ice core reflect source and transport changes between Last Glacial Maximum and the early Holocene. *Global Change Biol* 24(5):2182-2197.

53. Boetius A, Anesio AM, Deming JW, Mikucki JA, & Rapp JZ (2015) Microbial ecology of the cryosphere: sea ice and glacial habitats. *Nature Reviews Microbiology* 13:677.
54. Adler CJ*, et al.* (2013) Sequencing ancient calcified dental plaque shows changes in oral microbiota with dietary shifts of the Neolithic and Industrial revolutions. *Nature Genetics* 45:450.
55. Turner CR, Miller DJ, Coyne KJ, & Corush J (2014) Improved methods for capture, extraction, and quantitative assay of environmental DNA from Asian bigheaded carp (*Hypophthalmichthys* spp.). *PloS one* 9(12):e114329.
56. Caporaso JG*, et al.* (2012) Ultra-high-throughput microbial community analysis on the Illumina HiSeq and MiSeq platforms. *The ISME Journal* 6:1621.
57. Bulat SA*, et al.* (2004) DNA signature of thermophilic bacteria from the aged accretion ice of Lake Vostok, Antarctica: implications for searching for life in extreme icy environments. *International Journal of Astrobiology* 3(1):1-12.
58. Hansen AA*, et al.* (2007) Viability, diversity and composition of the bacterial community in a high Arctic permafrost soil from Spitsbergen, Northern Norway. *Environmental microbiology* 9(11):2870-2884.
59. Caporaso JG*, et al.* (2010) QIIME allows analysis of high-throughput community sequencing data. *Nat Methods* 7(5):335-336.
60. DeSantis TZ*, et al.* (2006) Greengenes, a chimera-checked 16S rRNA gene database and workbench compatible with ARB. *Applied and Environmental Microbiology* 72(7):5069-5072.
61. Chen T*, et al.* (2010) The Human Oral Microbiome Database: a web accessible resource for investigating oral microbe taxonomic and genomic information. *Database* 2010:baq013-baq013.

62. Salter SJ, *et al.* (2014) Reagent and laboratory contamination can critically impact sequence-based microbiome analyses. *BMC Biology* 12(1):87.

63. Xin J-y, *et al.* (2004) Production of methanol from methane by methanotrophic bacteria. *Biocatalysis and Biotransformation* 22(3):225-229.

64. Hanson RS & Hanson TE (1996) Methanotrophic bacteria. *Microbiological Reviews* 60(2):439-471.

65. Paul BG, *et al.* (2017) Methane-oxidizing bacteria shunt carbon to microbial mats at a marine hydrocarbon seep. *Frontiers in Microbiology* 8(186).

66. Wadham JL, *et al.* (2013) The potential role of the Antarctic Ice Sheet in global biogeochemical cycles. *Earth and Environmental Science Transactions of the Royal Society of Edinburgh* 104(1):55-67.

67. Bueler E, Lingle CS, & Brown J (2007) Fast computation of a viscoelastic deformable Earth model for ice-sheet simulations. *Annals of Glaciology* 46:97-105.

68. Feldmann J, Albrecht T, Khroulev C, Pattyn F, & Levermann A (2014) Resolution-dependent performance of grounding line motion in a shallow model compared with a full-Stokes model according to the MISMIP3d intercomparison. *Journal of Glaciology* 60(220):353-360.

69. Bueler E & Brown J (2009) Shallow shelf approximation as a "sliding law" in a thermomechanically coupled ice sheet model. *Journal of Geophysical Research: Earth Surface* 114(F3):doi: 10.1029/2008JF001179.

70. Albrecht T & Levermann A (2012) Fracture field for large-scale ice dynamics. *Journal of Glaciology* 58(207):165-176.

71. Levermann A, *et al.* (2012) Kinematic first-order calving law implies potential for abrupt ice-shelf retreat. *The Cryosphere* 6:273-286.

72. Seguinot J (2013) Spatial and seasonal effects of temperature variability in a positive degree-day glacier surface mass-balance model. *Journal of Glaciology* 59(218):1202-1204.

73. Rogozhina I & Rau D (2014) Vital role of daily temperature variability in surface mass balance parameterizations of the Greenland Ice Sheet. *The Cryosphere* 8(2):575-585.

74. Golledge NR*, et al.* (2015) The multi-millennial Antarctic commitment to future sea-level rise. *Nature* 526(7573):421-425.

75. Frieler K*, et al.* (2015) Consistent evidence of increasing Antarctic accumulation with warming. *Nature Climate Change* 5(4):348-352.

76. Thompson SL & Pollard D (1997) Greenland and Antarctic mass balances for present and doubled atmospheric CO_2 from the GENESIS version-2 global climate model. *Journal of Climate* 10(5):871-900.

77. Hellmer HH, Jacobs SS, & Jenkins A (1998) Oceanic erosion of a floating Antarctic glacier in the Amundsen Sea. *Ocean, Ice, and Atmosphere: Interactions at the Antarctic Continental Margin,* Antarctic Research Series, eds Jacobs S & Weiss R (AGU, Washington D. C.

), pp 75–319.

78. Holland DM & Jenkins A (1999) Modeling thermodynamic ice–ocean interactions at the base of an ice shelf. *Journal of Physical Oceanography* 29(8):1787-1800.

79. Golledge NR, Levy RH, McKay RM, & Naish TR (2017) East Antarctic ice sheet most vulnerable to Weddell Sea warming. *GRL* 44(5):2343-2351.

80. Mengel M & Levermann A (2014) Ice plug prevents irreversible discharge from East Antarctica. *Nature Climate Change* 4:451-455.

81. Aitken ARA*, et al.* (2016) Repeated large-scale retreat and advance of Totten Glacier indicated by inland bed erosion. *Nature* 533(7603):385-389.

82. Fretwell P*, et al.* (2013) Bedmap2: improved ice bed, surface and thickness datasets for Antarctica. *The Cryosphere* 7(1):375-393.

83. Comiso JC (2000) Variability and trends in Antarctic surface temperatures from in situ and satellite infrared measurements. *Journal of Climate* 13(10):1674-1696.

84. Lenaerts JTM, van den Broeke MR, van de Berg WJ, van Meijgaard E, & Kuipers Munneke P (2012) A new, high-resolution surface mass balance map of Antarctica (1979–2010) based on regional atmospheric climate modeling. *GRL* 39(4):doi: 10.1029/2011GL050713.

85. Wessel P & Smith WH (1998) New, improved version of Generic Mapping Tools released. *Eos, Transactions American Geophysical Union* 79(47):579-579.

86. Le Maitre RW (1989) *A Classification of Igneous Rocks and Glossary of Terms* (Basil Blackwell, Oxford).

87. Golledge NR*, et al.* (2017) Antarctic climate and ice-sheet configuration during the early Pliocene interglacial at 4.23 Ma. *Clim Past* 13(7):959-975.

88. Haran T, Bohlander J, Scambos T, Painter T, & Fahnestock M (2006) MODIS Mosaic Image of Antarctica. in *National Snow and Ice Data Center, Boulder, Colo., Digital Media* (Boulder, Colorado).

89. Rignot E, Mouginot J, & Scheuchl B (2011) Ice flow of the Antarctic Ice Sheet. *Science* 333(6048):1427-1430.

90. Golledge NR, Fogwill CJ, Mackintosh AN, & Buckley KM (2012) Dynamics of the Last Glacial Maximum Antarctic ice-sheet and its response to ocean forcing. *PNAS* 109:16052–16056.

91. Kuehn SC, Froese DH, & Shane PAR (2011) The INTAV intercomparison of electron-beam microanalysis of glass by tephrochronology laboratories: Results and recommendations. *Quaternary International* 246:19-47.

92. Wilson SA (1997) The collection, preparation and testing of USGS reference material BCR-2, Columbia River, Basalt. in *U.S. Geological Survey Open-File Report*.
